# Mode-pairing quantum key distribution

**DOI:** 10.1038/s41467-022-31534-7

**Published:** 2022-07-07

**Authors:** Pei Zeng, Hongyi Zhou, Weijie Wu, Xiongfeng Ma

**Affiliations:** Center for Quantum Information, Institute for Interdisciplinary Information Sciences, Tsinghua University, Beijing, 100084 China

**Keywords:** Quantum information, Quantum optics

## Abstract

Quantum key distribution — the establishment of information-theoretically secure keys based on quantum physics — is mainly limited by its practical performance, which is characterised by the dependence of the key rate on the channel transmittance *R*(*η*). Recently, schemes based on single-photon interference have been proposed to improve the key rate to $R=O(\sqrt{\eta })$ by overcoming the point-to-point secret key capacity bound with interferometers. Unfortunately, all of these schemes require challenging global phase locking to realise a stable long-arm single-photon interferometer with a precision of approximately 100 nm over fibres that are hundreds of kilometres long. Aiming to address this problem, we propose a mode-pairing measurement-device-independent quantum key distribution scheme in which the encoded key bits and bases are determined during data post-processing. Using conventional second-order interference, this scheme can achieve a key rate of $R=O(\sqrt{\eta })$ without global phase locking when the local phase fluctuation is mild. We expect this high-performance scheme to be ready-to-implement with off-the-shelf optical devices.

## Abstract (web summary)

Measurement-device-independent QKD schemes suffer from a trade-off between ease of implementation (avoiding the need for global phase locking) and high rates (quadratic improvement in rate). Here, the authors propose a protocol which offers both simple implementation and strong performances.

## Introduction

Quantum key distribution (QKD)^1,2^ is currently the most successful application of quantum information science and serves as the first stepping stone towards a future quantum communication network^3^. A core advantage of QKD compared to other quantum communication tasks is that it is ready to implement with current commercially available off-the-shelf optical devices. However, two major characteristics of QKD—its practical security and key-rate performance—limit its real-life implementation. The key generation speed suffers heavily from transmission loss in the optical channel. Fundamentally, the asymptotic key rate for point-to-point QKD schemes is upper bounded by the repeaterless rate-transmittance bounds^4,5^, which are approximately linear functions of the transmittance, *R* ≤ *O*(*η*). For example, when *η* is small, the PLOB repeaterless rate-transmittance bound^5^ is about 1.44*η*. Quantum repeaters^6–8^ have been proposed as a radical solution to this problem. Unfortunately, none of the quantum repeater proposals is easy to implement in the near term.

In real-life use, the deviation of the realistic behaviour of physical devices from their ideal ones gives rise to critical issues in practical security. There are many quantum attacks that can take advantage of the loopholes introduced by device imperfections^9^. A typical QKD system can be divided into three parts: source, channel, and measurement. The security of the channel has been well addressed in the security proofs for QKD^10–12^. The source is relatively simple and can be well characterised^13^. In contrast, the measurement device is complicated and difficult to calibrate. Moreover, an adversary could manipulate the measurement device by sending unexpected signals^14,15^. To solve this implementation security problem, measurement-device-independent quantum key distribution (MDI-QKD) schemes have been proposed to close the detection loopholes once and for all^16^. Various experimental systems have been successfully demonstrated^17–20^, with extension to a communication network^21^.

**Fig. 1:**
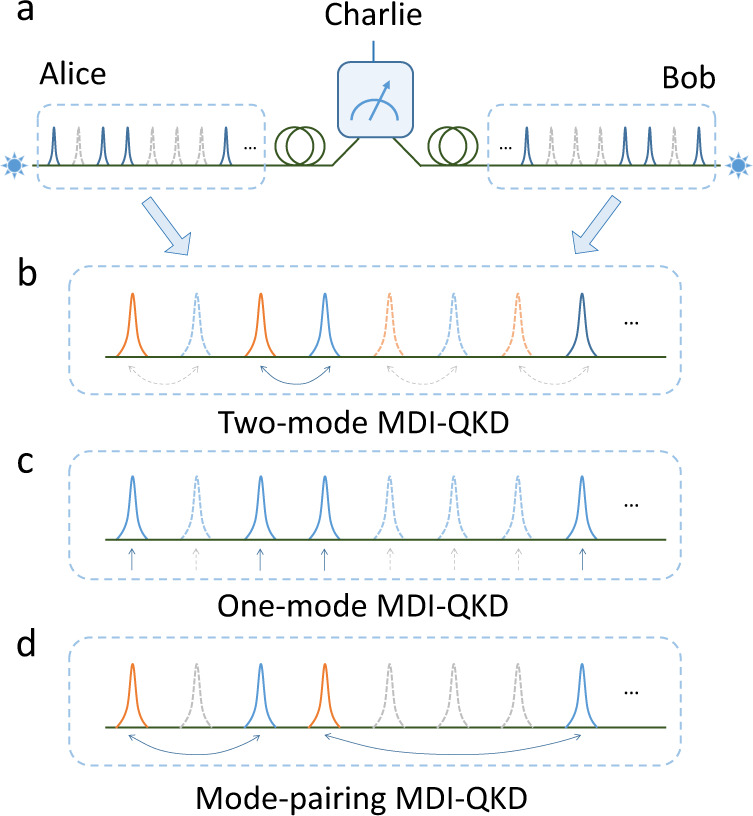
Comparison of two-mode, one-mode and mode-pairing MDI-QKD schemes. **a** Schematic diagram of a generic MDI-QKD scheme. The solid and dashed pulses yield successful and unsuccessful detection, respectively, at the measurement site. For **b**, **c** and **d**, each wave packet in the diagram represents two independent pulses emitted simultaneously by Alice and Bob. **b** In two-mode MDI-QKD schemes, the pairing of the blue pulses (as phase references) and orange pulses (as signals) is predetermined, necessitating coincidence detection. **c** In one-mode MDI-QKD schemes (e.g., twin-field quantum key distribution and its variants), there is no phase reference pulse, necessitating global phase locking. **d** In the mode-pairing MDI-QKD scheme, in accordance with the detection results, Alice and Bob pair the clicked pulses and assign them to be either reference or signal pulses, such that neither coincidence detection nor global phase locking is required.

A generic MDI-QKD setup is shown in Fig. 1a. Each of the two communicating parties, Alice and Bob, holds a quantum light source, encodes random bits into quantum pulses, and sends these pulses to a measurement site through lossy channels. Measurement devices are possessed by an untrusted party, Charlie, who is supposed to correlate Alice’s and Bob’s signals via interference detection. Based on the detection results announced by Charlie, Alice and Bob sift the local random bits encoded in the pulses to generate secure key bits. Note that the security of MDI-QKD schemes does not rely upon the physical implementation of the detection devices. Alice and Bob need to trust only their own locally encoded quantum sources. Since neither Alice nor Bob receives quantum signals from the channel during key distribution, any hacker’s attempt to manipulate the users’ devices becomes extremely difficult compared to regular QKD schemes^14,15^.

Strictly speaking, MDI-QKD is not a point-to-point scheme, as there is an interference site between Alice and Bob. Consequently, it is not necessarily limited by the repeaterless rate-transmittance bound. Nevertheless, the original MDI-QKD scheme^16^, in which Alice and Bob both encode a ‘dual-rail’ qubit into a single-photon subspace on two polarization modes, unfortunately, cannot overcome this bound. Later, alternative schemes were proposed^22,23^ in which the qubit is encoded into two optical time bins. We refer to schemes of this type as two-mode MDI-QKD, in the sense that the single-side key information is encoded in the relative phase of the coherent states in the two orthogonal optical modes, i.e., second-quantized electromagnetic fields. To correlate Alice’s and Bob’s encoded information in a two-mode scheme, a successful two-photon interference measurement is required. If either Alice or Bob’s emitted photon is lost in transmission, there will be no conclusive detection result. For example, in the time-bin encoding scheme^23^ shown in Fig. 1b, Alice and Bob each emit a qubit encoded in two time-bin modes, with Alice emitting *A*_1_ and *A*_2_ and Bob emitting *B*_1_ and *B*_2_. Only when both the interference between modes *A*_1_ and *B*_1_ and that between *A*_2_ and *B*_2_ yield successful detection can Alice restore Bob’s raw key information. Thus, successful interference requires a coincidence detection. Due to this coincidence-detection requirement, rounds with only a single detection are discarded, resulting in a relatively low key generation rate—one that is a linear function of the transmittance, *O*(*η*). From the perspective of practical implementation, however, coincidence detection also has certain merits. This approach can ensure stable optical interference, while Alice and Bob need only to stabilise the relative phases between the two modes.

Coincidence detection is the essential factor that prevents MDI-QKD from overcoming the linear key-rate bound. To eliminate this requirement, a new type of MDI-QKD scheme called twin-field quantum key distribution (TF-QKD) based on encoding information into a single-optical mode have been proposed^24^, illustrated in Fig. 1c. Later on, variants of TF-QKD have been proposed, among which the key information in encoded in either the phase^25,26^ (known as phase-matching QKD) or the intensity^27^ (known as sending-or-not-sending TF-QKD) of coherent states. In this work, we refer to these twin-field-type schemes as one-mode MDI-QKD schemes for a conceptual comparison to the traditional two-mode MDI-QKD schemes, since the single-side information in these schemes is encoded into a single-optical mode in each round. We remark that the single-optical-mode encoding MDI-QKD scheme was first proposed in ref. ^28^ as “MDI-B92” scheme. Similar to the Duan-Lukin-Cirac-Zoller-type repeater design^29^, such one-mode schemes use single-photon interference instead of coincidence detection, hence yielding a quadratic improvement in key rate compared to two-mode schemes^24–26^. As a result, they can overcome the point-to-point linear key-rate bound^4,5^. Unfortunately, one-mode schemes are more challenging to implement due to the unstable optical interference resulting from the lack of global phase references. For example, in the phase-matching QKD (PM-QKD) scheme^25^, the key information is encoded into the global phase of Alice’s and Bob’s coherent states. The phases of the coherent states generated by two remote and independent lasers need to be matched at the measurement site. A small phase drift or fluctuation caused by the lasers and/or channels is hazardous for key generation.

At first glance, it seems that we cannot simultaneously enjoy the advantages of one-mode schemes (i.e., quadratic improvement in successful detection) and two-mode schemes (i.e., stable optical interference), due to an intrinsic trade-off between the information-encoding efficiency and robustness. On the one hand, the relative information among different optical modes is more difficult to retrieve when the channel loss is large. On the other hand, the global phase of a coherent state is not as stable as the relative phase between two coherent states travelling through the same quantum channel. In a typical 200-km fibre with a telecommunication frequency of 1550 nm, the phase of a coherent state is susceptible to small fluctuations in the optical transmission time (~10^−15^ s), optical length (~200 nm) and light frequency (~100 kHz). Recently, experimentalists have made great efforts to demonstrate high-performance in one-mode schemes, utilising high-end technologies to perform a precise control operation to stabilise the global phase by locking the frequency and phase of the coherent states^30–37^. However, this increases the experimental difficulty and undermines the applicability of one-mode schemes in real life.

In this work, we propose a mode-pairing MDI-QKD scheme that aims to offer both—simple implementation and high performance. Hereafter, we refer to this scheme as the mode-pairing scheme for simplicity. By observing that the majority of detection events are single-clicks and are discard in the two-mode MDI-QKD schemes, we try to recycle the discarded single-click in the mode-pairing scheme. To do that, the coherent states in the transmitted modes are initially prepared independently with randomly encoded information. Based on the fact that the two detection events used to read out the encoded information do not need to occur at two predetermined locations, the key is extracted from two paired detection events rather than coincidence detection, as shown in Fig. 1d. This offers a quadratic improvement akin to that of one-mode schemes when the local phases can be stabilized using currently available phase stabilization techniques. Moreover, key information about the mode-pairing scheme is encoded in the relative phases or intensities, whose stability relies only upon the conditions of the local phase references and optical paths. Therefore, the technical complexity is similar to that of two-mode schemes, which have been widely implemented both in the laboratory^17–19,38^ and in the field^21,39^. Notably, to adapt to different hardware conditions, the mode-pairing scheme can be freely tuned between the one-mode and two-mode schemes by adjusting a pulse-interval parameter (as discussed later in Results’ subsection “Pairing strategy”) during data postprocessing to optimise the system performance.

## Results

### Mode-pairing scheme

**Fig. 2:**
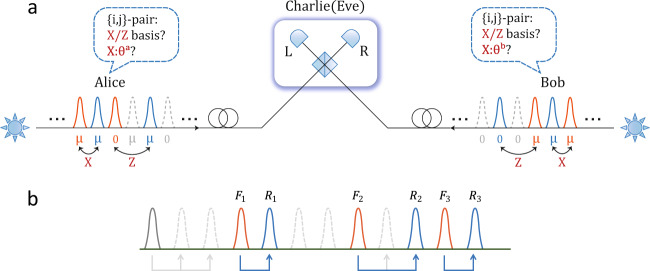
Schematic diagram of the mode-pairing MDI-QKD scheme and the simple pairing strategy with maximal-pairing interval *l*. The solid and dashed pulses are those with and without successful detection, respectively. Orange and blue pulses are, respectively, the front and rear pulses that succeed in pairing within *l* pulses, while grey pulses are the ones fail in pairing. **a** In the mode-pairing MDI-QKD scheme, Alice and Bob, first prepare coherent pulses with random intensities chosen from {0, *μ*} and random phases ${\phi }_{i}^{a(b)}\in [0,2\pi )$ and send them to Charlie. After interference measurement, Charlie announces the detection results, based on which Alice and Bob pair the pulses and determine their encoding bases. For *X*-pairs, they announce the alignment angles *θ*^*a*^ and *θ*^*b*^ and keep data for which *θ*^*a*^ = *θ*^*b*^. They use *Z*-pairs to generate keys and other data for parameter estimation. **b** We set *l* = 2 in the simple pairing strategy for example. The labels *F*_*k*_ and *R*_*k*_ represent the front and rear pulses, respectively, in the *k*-th successful pair.

In the mode-pairing scheme, Alice and Bob first prepare coherent states with independently and randomly chosen intensities and phases in each emitted optical mode. These coherent states are sent to the untrusted measurement site, Charlie. Based on Charlie’s announced measurement results, Alice and Bob pair the optical modes with successful detection and determine the key bits and bases for each mode pair locally. They then sift the bases and generate secure key bits via postprocessing. The scheme is introduced in Box 1 and illustrated in Fig. 2a. For simplicity of the introduction of the main protocol design, we omit the details of the decoy-state method^40^ and discrete phase randomisation here. A complete description of the mode-pairing scheme is given in the Methods’ subsection “Mode-pairing scheme with decoy states”.

In the mode-pairing scheme, we mainly consider the keys generated from the *Z*-pair data, since they have a much lower quantum bit error rate ${E}_{\mu \mu }^{Z}$ than the *X*-pair data. The encoding of the mode-pairing scheme in Box 1 originates from the time-bin encoding MDI-QKD scheme^23^. If Alice’s two paired optical modes {*A*_*i*_, *A*_*j*_} are assigned to the *Z*-basis, then the state of the two optical modes is either ${\big|0\big\rangle }_{{A}_{i}}{\big|\sqrt{\mu }{{{{{{{{\rm{e}}}}}}}}}^{{{{{{{{\rm{i}}}}}}}}{\phi }_{j}^{a}}\big\rangle }_{{A}_{j}}$ or ${\left|\sqrt{\mu }{{{{{{{{\rm{e}}}}}}}}}^{{{{{{{{\rm{i}}}}}}}}{\phi }_{i}^{a}}\right\rangle }_{{A}_{i}}{\left|0\right\rangle }_{{A}_{j}}$, where ${\phi }_{i}^{a}$ and ${\phi }_{j}^{a}$ are two independent random phases. We can write the encoded states in a unified form:

1 $${\left|{\psi }_{Z}^{a}\right\rangle }_{{A}_{i},{A}_{j}}={\left|\sqrt{{\kappa }^{a}\mu }{{{{{{{{\rm{e}}}}}}}}}^{{{{{{{{\rm{i}}}}}}}}{\phi }_{i}^{a}}\right\rangle }_{{A}_{i}}\big|\sqrt{{\bar{\kappa }}^{a}\mu} {{{{{\rm{e}}}}}}^{{{{{{{{\rm{i}}}}}}}}{\phi }_{j}^{a}}\big\rangle _{{A}_{j}},$$

where *κ*^*a*^ is the encoded key information and $\bar{\kappa }:= \kappa \oplus 1$ is the inverse of *κ*. In the other case, in which the two optical modes {*A*_*i*_, *A*_*j*_} are assigned to the *X*-basis, we can rewrite their two independent random phases ${\phi }_{i}^{a}$ and ${\phi }_{j}^{a}$ as

2 $$\begin{array}{l}{\phi }^{a}:= {\phi }_{i}^{a}\in [0,2\pi ),\\ {\phi }_{\delta }^{a}:= {\phi }_{j}^{a}-{\phi }_{i}^{a}\in [0,2\pi ).\end{array}$$

In this way, the phase *ϕ*^*a*^ becomes a global random phase on the pulse pair, while ${\phi }_{\delta }^{a}$ is the relative phase for quantum information ‘encoding’. Due to the independence of ${\phi }_{i}^{a}$ and ${\phi }_{j}^{a}$, the phases *ϕ*^*a*^ and ${\phi }_{\delta }^{a}$ are also independent of each other and uniformly range from [0, 2*π*). By definition, we have ${\phi }_{\delta }^{a}={\theta }^{a}+\pi {\kappa }^{a}$. Then, the *X*-pair state can be written as,

3 $${\left|{\psi }_{X}^{a}\right\rangle }_{{A}_{i},{A}_{j}}={\left|\sqrt{{\mu }^{a}}{{{{{{{{\rm{e}}}}}}}}}^{{{{{{{{\rm{i}}}}}}}}{\phi }^{a}}\right\rangle }_{{A}_{i}}{\big|\sqrt{{\mu }^{a}}{{{{{{{{\rm{e}}}}}}}}}^{{{{{{{{\rm{i}}}}}}}}({\phi }^{a}+{\theta }^{a}+{\kappa }^{a}\pi )}\big\rangle }_{{A}_{j}},$$

where *μ*^*a*^ ∈ {0, *μ*}. When *θ* = 0 or *π*/2, Alice emits *X*-basis or *Y*-basis states, respectively, as used in the time-bin encoding MDI-QKD scheme^23^.

We remark that in either the *Z*-pair state in Eq. (1) or the *X*-pair state in Eq. (3), there is a global random phase *ϕ*^*a*^, which will not be revealed publicly. With this (global coherent state) phase randomisation, the emitted *Z*- and *X*-pair states can be regarded as a mixture of photon number states^40^. Then, Alice and Bob can estimate the detections caused by the pairs where they both emit single photons and use them to generate secure keys, in a manner similar to traditional two-mode schemes. Therefore, the security of the mode-pairing scheme is similar to that of two-mode schemes. Nevertheless, the mode-pairing scheme in Box 1 has the following unique features.

1. The emitted states in different optical modes {*A*_*i*_} are independent and identically distributed (i.i.d.). Therefore, the information encoded in different optical modes is completely decoupled.
2. Based on the postselection of clicked signals, different optical modes are paired afterwards. The relative information between the two modes is then converted into raw key data.

In the mode-pairing scheme, the key information is determined not in the state preparation step, but by the detection location, sharing some similarities with the differential-phase-shifting QKD scheme^41,42^. It is the untrusted measurement site that determines the location of successful detection and thereby affects the pairing setting. The ‘dual-rail’ qubits encoded on the single photons are ‘postselected’ on the basis of this detection. By virtual of the independence of the optical modes, the information encoded in the ‘postselected’ qubits cannot be revealed from other optical pulses.

For another comparison, the sending-or-not-sending (SNS) TF-QKD scheme^27^ also uses a *Z*-basis time-bin encoding, whereby either Alice or Bob emits an optical mode to generate key bits. The state preparation of the mode-pairing scheme shares similarities with the SNS-TFQKD scheme. However, the information of the mode-pairing scheme is encoded into the relative information between the two optical modes. As a result, the basis-sifting and key mapping of the mode-pairing scheme follow different logic originated from the time-bin encoding MDI-QKD scheme^23^. Note that in the SNS scheme, bits 0 and 1 are highly biased in the *Z* basis, whereas in the mode-pairing scheme, they are evenly distributed.

A critical issue in the security analysis of the mode-pairing scheme is to maintain the flexibility to determine in which two optical modes to perform the overall photon number measurement until Charlie announces the detection results. Note that, in the original two-mode QKD schemes, the encoders can always be assumed to perform an overall photon number measurement and post-select the single-photon components as good ‘dual-rail’ qubits before they emit their signals to Charlie. In the mode-pairing scheme, however, this is not viable because the optical pulse pair, for which the single-photon component is defined, is postselected based on Charlie’s detection announcement. To solve this problem, we introduce source replacement for the random phases in the coherent states to purify them as ancillary qudits and define an indirect overall photon number measurement on them. The source-replacement procedure can be found in the Methods’ subsection “Source replacement of the encoding state”. Conditioned on the indirect overall photon number measurement result to be single-photon states, the *X*-basis error rate fairly estimates the *Z*-basis phase error rate for the signals for which Alice and Bob both emit single photons.

In Supplementary Note 2, we provide a detailed security proof based on entanglement distillation. The main idea is to introduce a ‘fixed-pairing’ scheme, in which the pairing setting, i.e., which locations are paired together, is predetermined and hence independent of Charlie’s announcement. We first prove that, with any given pairing setting, the fixed-pairing scheme is secure, as it can be reduced to a two-mode MDI-QKD scheme. Afterwards, we examine the private state generated by the mode-pairing scheme and prove that it is the same as that of a fixed-pairing scheme under all possible measurements that Charlie could perform and announcement methods. In this way, we prove the equivalence of the mode-pairing scheme to a group of fixed-pairing schemes with different pairing settings.

Box 1 Mode-pairing scheme

1. **State preparation**: In the *i*-th round (*i* = 1, 2, . . . , *N*), Alice prepares a coherent state $\left|\sqrt{{\mu }_{i}^{a}}{{{{{{{{\rm{e}}}}}}}}}^{{{{{\rm{i}}}{\phi }_{i}^{a}}}}\right\rangle$ in optical mode *A*_*i*_ with an intensity ${\mu }_{i}^{a}$ randomly chosen from {0, *μ*} and a phase ${\phi }_{i}^{a}$ uniformly chosen from [0, 2*π*). Similarly, Bob randomly chooses ${\mu }_{i}^{b}$ and ${\phi }_{i}^{b}$ and prepares $\big|\sqrt{{\mu }_{i}^{b}}{{{{{{{{\rm{e}}}}}}}}}^{{{{{{{{\rm{i}}}}}}}}{\phi }_{i}^{b}}\big\rangle$ in mode *B*_*i*_.
2. **Measurement**: Alice and Bob send modes *A*_*i*_ and *B*_*i*_ to Charlie, who performs single-photon interference measurements. Charlie announces the click patterns for both detectors *L* and *R*.
  Alice and Bob repeat the above two steps for *N* rounds. Then, they postprocess the data as follows.
3. **Mode pairing**: For all rounds with successful detection, in which one and only one of the two detectors clicks, Alice and Bob apply a strategy of grouping two clicked rounds as a pair. The encoded phases and intensities in these two rounds form a data pair. A simple pairing strategy is introduced in Box 2.
4. **Basis sifting**: Based on the intensities of the two grouped rounds indexed by *i* and *j*, Alice labels the ‘basis’ of the data pair as *Z* if the intensities are (0, *μ*) or (*μ*, 0), as *X* if the intensities are (*μ*, *μ*), or as ‘0’ if the intensities are (0, 0). Bob sets the basis using the same method. Alice and Bob announce the basis of each data pair; if they both announce the basis *X* or *Z*, they maintain the data pairs, whereas otherwise, the data pairs are discarded.
5. **Key mapping**: For each *Z*-basis pair (*Z*-pair for simplicity) at locations *i* and *j*, Alice sets her key as *κ*^*a*^ = 0 if $({\mu }_{i}^{a},{\mu }_{j}^{a})=(0,\mu )$ and *κ*^*a*^ = 1 if $({\mu }_{i}^{a},{\mu }_{j}^{a})=(\mu ,0)$. For each *X*-basis pair (*X*-pair for simplicity) at locations *i* and *j*, the key is extracted from the relative phase $({\phi }_{j}^{a}-{\phi }_{i}^{a})={\theta }^{a}+\pi {\kappa }^{a}$, where the raw key bit is ${\kappa }^{a}=\left\lfloor (({\phi }_{j}^{a}-{\phi }_{i}^{a})/\pi \,{{\mbox{mod}}}\,2)\right\rfloor$ and the alignment angle is ${\theta }^{a}:=({\phi }_{j}^{a}-{\phi }_{i}^{a})\,{{\mbox{mod}}}\,\pi$. In a similar way, Bob assigns his raw key bit *κ*^*b*^ and determines *θ*^*b*^. The difference in the key mapping for *Z*-pairs is that, Bob sets the raw key bit *κ*^*b*^ as 0 if $({\mu }_{i}^{b},{\mu }_{j}^{b})=(\mu ,0)$ and *κ*^*b*^ = 1 if $({\mu }_{i}^{b},{\mu }_{j}^{b})=(0,\mu )$. As an extra step on the *X*-pairs, if Charlie’s detection announcement is (*L*, *L*) or (*R*, *R*), Bob keeps the bit *κ*^*b*^; otherwise, if Charlie’s announcement is (*L*, *R*) or (*R*, *L*), Bob flips *κ*^*b*^. For the *X*-pairs, Alice and Bob announce the alignment angles *θ*^*a*^ and *θ*^*b*^. If *θ*^*a*^ = *θ*^*b*^, then the data pairs are kept; otherwise, the data pairs are discarded.
6. **Parameter estimation**: Alice and Bob estimate the fraction of clicked signals *q*_(1, 1)_ and the corresponding phase error rate ${e}_{(1,1)}^{X}$ of *Z*-pairs where Alice and Bob both emit a single photon at locations *i* and *j*, using the data of the *Z*-pairs and *X*-pairs. They also estimate the quantum bit error rate *E*^(*μ*, *μ*),*Z*^ of the *Z*-pairs.
7. **Key distillation**: Alice and Bob use the *Z*-pairs to generate a key. They perform error correction and privacy amplification on the basis of *q*_(1, 1)_, *E*^(*μ*, *μ*),*Z*^ and ${e}_{11}^{X}$.

### Pairing strategy

The pairing strategy mentioned in Step 3 lies at the core of the mode-pairing scheme in Box 1, which correlates two independent signals and determines their bases and key bits. Note that the relative phase between two paired quantum signals determines the key information on the *X* basis. When the time interval between these two pulses becomes too large, the key information suffers from phase fluctuation, which is characterised by the laser coherence time. Therefore, Alice and Bob should establish a maximal pairing interval *l*, such that the number of pulses between the two paired signals should not exceed *l*. In practice, *l* can be estimated by multiplying the laser coherence time by the system repetition rate.

Here, we consider a simple pairing strategy in which Alice pairs adjacent detection pulses together if the time interval between them is not too large (≤*l*). The details are shown in the simple pairing strategy in Box 2 and illustrated in Fig. 2b. Charlie’s announcement in the *i*-th round is denoted by a Boolean variable *C*_*i*_ that indicates whether the detection is successful. That is, *C*_*i*_ = 1 implies that either the detector *L* or *R* clicks. Otherwise, there is no click or double clicks.

To check the efficiency of this pairing strategy, let us calculate the pairing rate *r*_*p*_ (i.e. the average number of pairs generated per pulse). We assume that Alice and Bob choose intensities 0 and *μ* with equal probability, maximising the number of successful pairs in the *Z* basis. With a typical QKD channel model, the pairing rate *r*_*p*_ is calculated as shown in the Methods’ subsection “Mode-pairing-efficiency calculation”,

4 $${r}_{p}(p,l)={\left[\frac{1}{p[1-{(1-p)}^{l}]}+\frac{1}{p}\right]}^{-1},$$

where *p* is the probability that the emitted pulses result in a click event, given approximately by *η*_*s*_*μ*. Here, *η*_*s*_ and *η* denote the channel transmittance from Alice to Charlie and the total transmittance from Alice to Bob, respectively. When the channel is symmetric for Alice and Bob, we have $\eta ={\eta }_{s}^{2}$. An explicit simulation formula for *p* in a pure-loss channel is given in Supplementary Note 4. Note that both the pairing ratio *r*_*p*_ and the detection probability *p* can be directly obtained by experimentation.

The raw key rate mainly depends on the pairing rate *r*_*p*_. Now, let us check the scaling of *r*_*p*_ with the channel transmittance in the symmetric-channel case. If the local phase reference is sufficiently stable, then the maximal interval can be set to *l* → +*∞*. In this case,

5 $${r}_{p}=\frac{p}{2}\approx \frac{{\eta }_{s}\mu }{2}=O(\sqrt{\eta }),$$

where the optimal intensity is *μ* = *O*(1), as evaluated in Supplementary Note 5. On the other hand, if the local phase reference is not at all stable, one must set *l* = 1; then,

6 $${r}_{p}=\frac{{p}^{2}}{1+p}\approx \frac{{\eta }_{s}^{2}{\mu }^{2}}{1+{\eta }_{s}\mu }=O(\eta ).$$

In this case, the experimental requirements for the mode-pairing scheme are close to those of the existing time-bin MDI-QKD scheme^23^. Now, if we consider a finite value of *l*, the dependence of *r*_*p*_(*p*, *l*) on *η* will be decided by how the denominator of the first term in Eq. (4), *p*[1 − (1−*p*)^*l*^], depends on *p* ≈ *η*_*s*_*μ*. When *pl* ≫ 1, *r*_*p*_(*p*, *l*) scales with *p* linearly, hence ${r}_{p}=O(\sqrt{\eta })$; when *pl* ≪ 1, it scales with *p*^2^, resulting in *r*_*p*_ = *O*(*η*). Around *pl* = 1, there will be a performance transition from ${r}_{p}=O(\sqrt{\eta })$ to *r*_*p*_ = *O*(*η*).

In practice, *l* can be adjusted in accordance with the laser quality and quantum-channel fluctuations. Note that *l* can also be adjusted during data postprocessing, offering flexibility for various environmental changes in real time. Generally, the whole pairing strategy can be adjusted through different realisations.

Box 2 Simple pairing strategy

### Practical issues and simulation

The key rate of the mode-pairing scheme, as rigorously analysed in the Supplementary Note 2, has a decoy-state MDI-QKD form:

7 $$R={r}_{p}{r}_{s}\left\{{q}_{(1,1)}\left[1-H({e}_{(1,1)}^{X})\right]-fH({E}^{(\mu ,\mu ),Z})\right\},$$

where *r*_*p*_ is the pairing rate contributed by each block, *r*_*s*_ is the proportion of *Z*-pairs among all the generated location pairs (~1/8), *q*_(1, 1)_ is the fraction of *Z*-pairs caused by single-photon-pair states *ρ*^(1, 1)^ in which both Alice and Bob send single-photon states in the two paired modes, ${e}_{(1,1)}^{X}$ is the phase error rate of the detection caused by *ρ*^(1, 1)^, *f* is the error-correction efficiency, and *E*^(*μ*, *μ*),*Z*^ is the bit error rate of the sifted raw data. The fraction *q*_(1, 1)_ and the phase error ${e}_{(1,1)}^{X}$ can be estimated using the decoy-state method^40,43,44^. A detailed estimation procedure for *q*_(1, 1)_ and ${e}_{(1,1)}^{X}$ with the vacuum + weak decoy-state method is introduced in Supplementary Note 3.

During the key mapping step in Box 1, the *X*-pair sifting condition *θ*^*a*^ = *θ*^*b*^ is impossible to fulfil exactly. This results in insufficient data for *X*-basis error rate estimation. To solve this problem, one can apply discrete phase randomisation^45^ such that *θ*^*a*^ and *θ*^*b*^ are chosen from a discrete set. We expect the discretisation effect to be negligible when the number of discrete phases is reasonably large, such as *D* = 16, similar to the situation in previous works on one-mode MDI-QKD^46^.

**Fig. 3:**
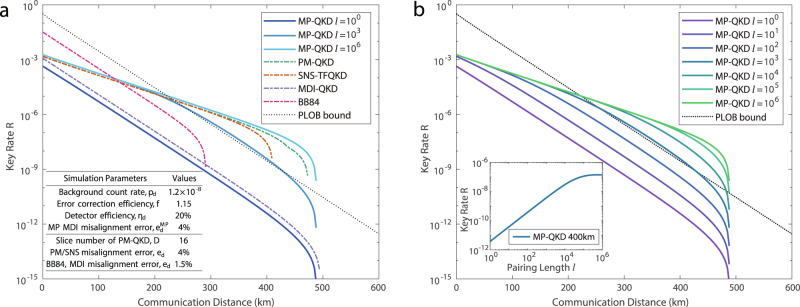
Asymptotic key-rate performance of the mode-pairing scheme. The horizontal axis representing the total communication distance with a fibre loss of 0.2 dB/km and the vertical axis representing the key generation rate. **a** Main Panel: Performance comparison of the mode-pairing scheme (denoted by MP-QKD in the plot) with the decoy-state Bennett-Brassard 1984 (BB84)^1,13,40^, MDI-QKD^16^, PM-QKD^25,46^, SNS-TFQKD^27,47^ schemes and the repeaterless rate-transmittance bound (PLOB bound)^5^. Inset: The simulation parameters used in the key-rate plot, which are mainly from ref. ^32^. **b** Main Panel: The rate-distance dependence of the mode-pairing scheme with different maximal-pairing intervals *l*. Inset: The key rate with respect to the pairing interval *l* for a communication distance of 400 km.

Based on the above analysis, we simulate the asymptotic performance of the mode-pairing scheme under a typical symmetric quantum-channel model, using practical experimental parameter settings. We assign the maximal pairing interval *l* of the mode-pairing scheme as a value between 1 and 1 × 10^6^, aiming to illustrate the dependence of the key rate on *l*. We also compare the key rate of the mode-pairing scheme with those of a typical two-mode scheme, time-bin encoding MDI-QKD^23^, and two one-mode schemes — PM-QKD^46^ and SNS-TFQKD^47^. The simulation results are shown in Fig. 3. We set the misalignment error rate of the mode-pairing scheme to be the same as the one-mode schemes for a fair comparison. In Supplementary Note 5, we show that the key-rate performance of the mode-pairing scheme is robust against misalignment errors. Even with a misalignment error rate of 15%, the mode-pairing scheme is able to surpass the repeaterless rate-transmittance bound with *l* = 2000. Here, we compare the asymptotic key-rate performance of all the schemes under the scenario of one-way local-operation and classical communication. The simulation formulas for these schemes are listed in Supplementary Note 4. Recently, researches^48,49^ show that the key-rate performance of SNS-TFQKD can be further improved by introducing the two-way classical communication^50,51^. We will leave the advanced key distillation for future studies.

As shown in Fig. 3a, the mode-pairing scheme with only neighbour pairing, *l* = 1, show a performance comparable to that of the original two-mode scheme. These two schemes have the same scaling property, i.e., *R* = *O*(*η*). The deviation is caused by an extra sifting factor in the mode-pairing scheme as a result of independent encoding. When the maximal pairing interval *l* is increased to 1 × 10^3^, the key rate is significantly enhanced by 3 orders of magnitude compared to the *l* = 1 case, making it able to surpass the linear key-rate bound. If we further increase *l* above 1 × 10^5^, then the mode-pairing scheme has a similar key rate to PM-QKD and SNS-TFQKD and a scaling property given by $R=O(\sqrt{\eta })$. In Fig. 3b, we further compare the key-rate performance of the mode-pairing scheme under different settings for *l*. When *l* falls within the range of 1 to 1 × 10^6^, the key rate of the mode-pairing scheme lies between the two extreme cases of *O*(*η*) and $O(\sqrt{\eta })$. The key-rate behaviour is dominated by the pairing rate given in Eq. (4).

In typical optical experiments, the typical line width of a common commercial laser is 3 kHz (see for example, ref. ^32^). Hence, the coherence time of the laser is around 333 μs. In practice, the frequency fluctuation of the lasers will affect the stabilization of the phase. To test the feasibility of the mode-pairing scheme, we perform an interference experiment using a commercial optical communication system with a repetition rate of 625 MHz. The experiment detail is shown in Supplementary Note 6. Based on the experimental data, we find that the phase coherence can be maintained well in a time interval of 5 μs, corresponding to *l* = 3000 ~ 4000. If we apply the state-of-the-art optical communication system with the repetition rate of 4 GHz^37^, we can realize a pairing interval over *l* = 20000. As an extra remark, our current discussion on the implementation of the mode-pairing scheme is based on the multiplexing of optical time-bin modes. Nonetheless, the proposed mode-pairing design is generic for the multiplexing of other optical degrees of freedom. For example, we can introduce frequency multiplexing. The optical modes with different frequencies are first prepared and interfered independently, i.e., only the pulses with the same frequency will be interfered. After the announcement of detection results, Alice and Bob then pair the locations with different frequencies during the post-processing. This can be used to increase the effective maximal pairing interval to an even larger value without the global phase locking. From Fig. 3b we can see that the key rate of the mode-pairing scheme with *l* = 1 × 10^4^ remains $R \sim O(\sqrt{\eta })$ when *η* is smaller than 30 dB, corresponding to a communication distance of 300 km. The asymptotic key rate of the mode-pairing scheme is 3 to 5 orders of magnitude higher than that of the two-mode scheme. We remark that the decoherence effect caused by the optical-fibre channel is negligible compared to the laser coherence time. When the fibre length is around 500 km, the velocity of phase drift in the fibre is <10 rad/ms^32^, which can be calibrated using strong laser pulses without the need for real-time feedback control. As a result, the value of *l* depends only upon the local phase reference and not the communication distance.

One advantage of the mode-pairing scheme is that it can be adapted to specific hardware conditions. In practice, optical systems may be unstable, causing the local phase reference to fluctuate rapidly. In this case, we can reduce the maximal pairing interval *l* and search for the optimal pairing strategy during the postprocessing procedure. As shown in the inset plot of Fig. 3b, the key rate of the mode-pairing scheme first increases linearly with increasing *l* before saturating when *l* is larger than ${p}^{-1}={(\mu \sqrt{\eta })}^{-1}$. In this case, Alice and Bob find successful detection within *l* locations with a high probability. Even when the optical system is unstable, the key rate can be nearly *l* times higher than that of the original time-bin MDI-QKD scheme when the value of *l* does not exceed ${p}^{-1}={(\mu \sqrt{\eta })}^{-1}$. We remark that, with the original experimental apparatus used in time-bin MDI-QKD, one can directly enhance the key rate by a factor of ~100 using the mode-pairing scheme. On the other hand, we note that for a given communication distance, *l* does not need to be very large to reach the maximal key-rate performance. For example, when the distance reaches 200 km, a maximal pairing interval of *l* = 1000 is sufficient to achieve the optimal key-rate performance. We leave a detailed evaluation for future research.

## Discussion

Based on a re-examination of the conventional two-mode MDI-QKD schemes and the recently proposed one-mode MDI-QKD schemes, we have developed a mode-pairing MDI-QKD scheme that retains the advantages of both, namely, achieving a high key rate with easy implementation. Since MDI-QKD schemes have the highest practical security level among the currently feasible QKD schemes, we expect the mode-pairing scheme paves the way for an optimal design for QKD, simultaneously enjoying high practicality, implementation security, and performance.

There remain several interesting directions for future work. Natural follow-up questions lie in the statistical analysis of the mode-pairing scheme in the finite-data-size regime and efficient parameter estimation. Due to the photon-number-based property of the mode-pairing scheme, previous studies of the statistical analysis of two-mode MDI-QKD schemes^52–54^ can be readily extended to analyse the mode-pairing scheme. To improve the efficiency of data usage, Alice and Bob may perform parameter estimation before basis sifting in order to use all signals that were originally discarded. On the other hand, one could design a mode-pairing scheme using the *X*-basis for key generation and the *Z*-basis for parameter estimation.

In this work, we employ a simple mode-pairing strategy based on pairing adjacent detection pulses. A more sophisticated pairing method might make bit and basis sifting more efficient. To improve the pairing strategy, Alice and Bob could reveal parts of the encoded intensity and phase information. For example, in the simple pairing strategy introduced in Box 2, Alice and Bob reveal the bases of the generated data pairs immediately after locations *i* and *j* are paired. If their basis choices differ, Alice and Bob ‘unpair’ locations *i* and *j*, and seek the next good pairing location for location *i* until the basis choices match.

To further enhance the performance, we could extend the mode-pairing design to other optical degrees of freedom, such as angular momentum and spectrum mode. Meanwhile, we could multiplex the usage of different degrees of freedom to enhance the repetition rate and extend the pairing interval *l*. Such multiplexing techniques would have additional benefits for the mode-pairing scheme. Suppose that we multiplex *m* quantum channels for a QKD task. In a normal setting, the key generation speed would be improved by a factor of *m*. For the mode-pairing scheme, in addition to this *m*-fold improvement, multiplexing would also introduce a larger pairing interval *ml*, since Alice and Bob would be able to pair quantum signals from different channels. A larger pairing interval *ml* would result in more paired signals and, hence, more key bits. Especially in the high-channel-loss regime where the distance between two clicked signals is large, the number of successful pairs becomes proportional to the maximum pairing interval *ml*. Thus, the key generation rate is proportional to *m*^2^ in the high-channel-loss regime.

Meanwhile, entanglement-based MDI-QKD schemes are essentially based on entanglement-swapping, which is the core design feature of quantum repeaters. The mode-pairing technique may help design a robust quantum repeater against a lossy channel. Note that our work shares similarities with the memory-assisted MDI-QKD protocol^55^ with quantum memories in the middle and with the all-photonic intercity MDI-QKD protocol^56^ with adaptive Bell-state measurement on the postselected photons. It is interesting to discuss the possibility of combining the mode-pairing design with an adaptive Bell-state measurement to tolerate more losses.

Moreover, the mode-pairing scheme has a unique feature in that the key bits are determined not in the encoding or measurement steps but upon postprocessing, which is an approach that can be further explored in other quantum communication tasks, including continuous-variable schemes.

## Methods

### Source replacement of the encoding state

The main idea of the security proof for the mode-pairing scheme is to introduce an entanglement-based scheme and reduce the security of the scheme to that of a traditional two-mode MDI-QKD scheme. To realise this, we perform a systematic source-replacement procedure^57,58^. Without loss of generality, in this subsection, we always assume the paired locations (*i*, *j*) to be (1, 2) to simplify the notations.

For convenience in the security proof, we slightly modify the scheme described in Box 1. First, we assume that the random phase of each mode is discretely chosen from a set of *D* phases, evenly distributed in [0, 2*π*). We expect the corresponding correction term in the security analysis due to the discretisation effect to be negligible^45,46^. Second, in the security proof, we modify the phase encoding and postprocessing procedures, as shown in Table 1. In the original scheme, Alice modulates *A*_1_ and *A*_2_ based on two random phases ${\phi }_{1}^{a}$ and ${\phi }_{2}^{a}$, respectively. During the *X*-basis processing, she calculates the relative phase difference ${\phi }_{\delta }^{a}:= {\phi }_{2}^{a}-{\phi }_{1}^{a}$ and splits it into an alignment angle *θ*^*a*^ in the range of [0, *π*) and a raw key bit *κ*^*a*^. We modify these procedures as follows: in addition to the two random phases ${\phi }_{1}^{a}$ and ${\phi }_{2}^{a}$, Alice also generates two bits ${z}_{1}^{^{\prime\prime} }$ and ${z}_{2}^{^{\prime\prime} }$ and applies extra phase modulations of ${z}_{1}^{^{\prime\prime} }\pi$ and ${z}_{2}^{^{\prime\prime} }\pi$ to *A*_1_ and *A*_2_, respectively. During the *X*-basis processing, she calculates the relative phase difference ${\phi }_{\delta }^{a}:= {\phi }_{2}^{a}-{\phi }_{1}^{a}$ and directly announces it for alignment-angle sifting. In the Supplementary Information, we prove the equivalence of these two encoding methods.

**Table 1:** Comparison of the phase encoding and postprocessing procedures of the mode-pairing scheme presented in the main text and the modified scheme considered in the security proof.

	Modulated phase	*X*-basis postprocessing	Sifting condition
Original scheme	${A}_{1}:{\phi }_{1}^{a},{A}_{2}:{\phi }_{2}^{a}$	${\theta }^{a}=({\phi }_{2}^{a}-{\phi }_{1}^{a}){{{{{{{\rm{mod}}}}}}}}\pi ,{\kappa }^{a}=\lfloor \frac{1}{\pi }({\phi }_{2}^{a}-{\phi }_{1}^{a}){{{{{{{\rm{mod}}}}}}}}2\rfloor$	*θ*^*a*^ = *θ*^*b*^
Modified scheme	${A}_{1}:{\phi }_{1}^{a}+{z}_{1}^{^{\prime\prime} }\pi ,{A}_{2}:{\phi }_{2}^{a}+{z}_{2}^{^{\prime\prime} }\pi$	${\theta }^{a}={\phi }_{2}^{a}-{\phi }_{1}^{a},{\kappa }^{a}={z}_{1}^{^{\prime\prime} }\oplus {z}_{2}^{^{\prime\prime} }$	*θ*^*a*^ − *θ*^*b*^ = 0 or *π*

In the modified scheme, Alice introduces an extra *π*-phase modulation for the storage of a bit ${z}_{1}^{^{\prime\prime} }$. This helps to decouple the phase randomisation and phase encoding analysis.

**Fig. 4:**
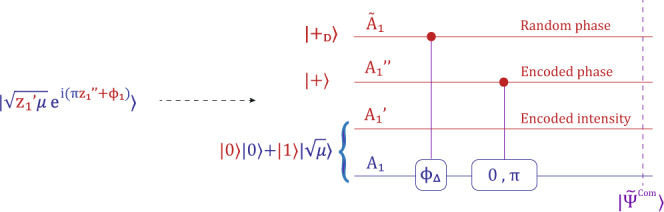
Source-replacement procedure for the mode-pairing scheme. We substitute the encoding of all random encoded information into the introduction of purified ancillary systems.

With the modification above, Alice further generates a random bit ${z}_{1}^{\prime}$ and a random dit (*d* = *D*) *j*_1_ in the first round. Based on the values of ${z}_{1}^{\prime}$, ${z}_{1}^{^{\prime\prime} }$ and ${j}_{1}^{a}$, she prepares the state

8 $$\big|{\psi}^{Com}\big\rangle=\big|\sqrt{{z}_{1}^{\prime}{\mu}}e^{{{{{{\rm{i}}}}}}(\pi{z}_{1}^{{\prime\prime}}+{\phi }_{1}^{a})}\big\rangle ,$$

with ${\phi }_{1}={j}_{1}\frac{2\pi }{D}$. As shown in Fig. 4, we substitute the encoding of random encoded information into the introduction of extra ancillary qubit and qudit systems labelled as ${\tilde{A}}_{1}$, ${A}_{1}^{^{\prime\prime} }$ and ${A}_{1}^{\prime}$. The purified encoding state is

9 $${\left|{\tilde{{{\Psi }}}}^{Com}\right\rangle }_{{\tilde{A}}_{1},{A}_{1}^{\prime},{A}_{1}^{^{\prime\prime} },{A}_{1}}=\frac{1}{2\sqrt{D}}\mathop{\sum }\limits_{{j}_{1}=0}^{D-1}{\left|{j}_{1}\right\rangle }_{{\tilde{A}}_{1}}{\left(\left|00\right\rangle \left|0\right\rangle +\left|01\right\rangle \left|0\right\rangle +\left|10\right\rangle \left|\sqrt{\mu }{e}^{i{\phi }_{1}^{a}}\right\rangle +\left|11\right\rangle \left|\sqrt{\mu }{e}^{i({\phi }_{1}^{a}+\pi )}\right\rangle \right)}_{{A}_{1}^{\prime},{A}_{1}^{^{\prime\prime} };{A}_{1}}.$$

In Fig. 4, we provide a specific state preparation procedure. The initial state is

10 $$\left|{+}_{D}\right\rangle 	:= \frac{1}{\sqrt{D}}\mathop{\sum }\limits_{j=0}^{D-1}\left|j\right\rangle ,\\ \left|+\right\rangle 	:= \left|{+}_{2}\right\rangle .$$

Here Alice applies a controlled-phase gate ${C}_{D}-\hat{U}({\phi }_{{{\Delta }}})$ with ${\phi }_{{{\Delta }}}:= \frac{2\pi }{D}$ from the qudit ${\tilde{A}}_{1}$ to optical mode *A*_1_. The controlled-phase gate is defined as

11 $${C}_{D}-\hat{U}{(\phi )}_{\tilde{A}A}:= \mathop{\sum }\limits_{j=0}^{D-1}{\left|j\right\rangle }_{\tilde{A}}\left\langle j\right|\otimes {e}^{{{{{{{{\rm{i}}}}}}}}\phi j{a}^{{{{\dagger}}} }a},$$

where *a*^†^ and *a* are the creation and annihilation operators, respectively, of mode *A*_1_. Alice also applies a controlled-phase gate $C-\hat{U}(\pi )$ from ${A}_{1}^{^{\prime\prime} }$ to *A*_1_.

**Fig. 5:**
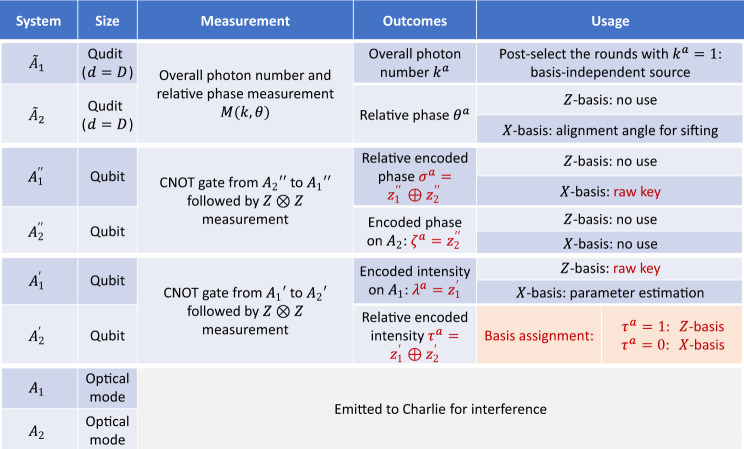
The quantum operations and usage of Alice’s encoding states on two paired locations (1, 2). There are 8 systems based on Alice's two paired locations. Among them, the two qudits ${\tilde{A}}_{1}$ and ${\tilde{A}}_{2}$ are measured to obtain the overall photon number *k*^*a*^ and the relative phase *θ*^*a*^ of two optical modes *A*_1_ and *A*_2_. The two qubits ${A}_{1}^{^{\prime\prime} }$ and ${A}_{2}^{^{\prime\prime} }$ are measured to obtain the relative phase, which is the raw key bit in the *X*-basis. Another two qubits ${A}_{1}^{\prime}$ and ${A}_{2}^{\prime}$ are measured to obtain the encoded intensity in *A*_1_ and the relative encoded intensity, which are used for the key mapping on the *Z*-basis and the basis assignment, respectively.

In the entanglement-based mode-pairing scheme, Alice and Bob generate the composite encoding state $\big|{\tilde{{{\Psi }}}}^{Com}\big\rangle$ defined in Eq. (9) in each round. They emit the optical modes to Charlie for interference. Based on Charlie’s announcement, they pair the locations and perform global operations on the corresponding ancillaries to generate raw key bits and useful parameters. In Fig. 5, we list the global operations performed on Alice’s paired locations. Among them, the relative encoded intensity ${\tau }^{a}:= {z}_{1}^{\prime}\oplus {z}_{2}^{\prime}$ is used to determine the basis choice. The encoded intensity ${\lambda }^{a}:= {z}_{1}^{\prime}$ and the relative encoded phase ${\sigma }^{a}={z}_{1}^{^{\prime\prime} }\oplus {z}_{2}^{^{\prime\prime} }$ are the raw key bits in the *Z*-basis and *X*-basis postprocessing, respectively.

A key point in our security proof is that we replace the random phases and register them into purified systems ${\tilde{A}}_{1}$ and ${\tilde{A}}_{2}$. This enables us to define a global measurement *M*(*k*, *θ*) on ${\tilde{A}}_{1}$ and ${\tilde{A}}_{2}$ to simultaneously obtain the overall photon number and the relative phase information encoded in optical modes *A*_1_ and *A*_2_. The construction of *M*(*k*, *θ*) is described in Supplementary Note 1. With the introduction of the purified systems ${\tilde{A}}_{1}$ and ${\tilde{A}}_{2}$ and the existence of the global measurement *M*(*k*, *θ*), Alice (same for Bob) is able to determine at which two locations to perform the global photon number measurement after Charlie’s announcement. With this measurement, Alice and Bob can further reduce the encoding state to a two-mode scheme. The detailed security proof is provided in Supplementary Note 2.

### Mode-pairing scheme with decoy states

Here, we present the mode-pairing scheme with an extra decoy intensity *ν* to estimate the parameters *q*_11_ and ${e}_{11}^{X}$. Of course, more decoy intensities can be applied in a similar manner.

1. **State preparation**: In the *i*-th round (*i* = 1, 2, . . . , *N*), Alice prepares a coherent state $\left|\sqrt{{\mu }_{i}^{a}}\exp ({{{{{{{\rm{i}}}}}}}}{\phi }_{i}^{a})\right\rangle$ in optical mode *A*_*i*_ with an intensity ${\mu }_{i}^{a}$ randomly chosen from {0, *ν*, *μ*} (0 < *ν* < *μ* < 1) and a phase ${\phi }_{i}^{a}$ uniformly chosen from the set ${\{\frac{2\pi }{D}k\}}_{k = 0}^{D-1}$. She records ${\mu }_{i}^{a}$ and ${\phi }_{i}^{a}$ for later use. Likewise, Bob chooses ${\mu }_{i}^{b}$ and ${\phi }_{i}^{b}$ randomly and prepares $\big|\sqrt{{\mu }_{i}^{b}}\exp ({{{{{{{\rm{i}}}}}}}}{\phi }_{i}^{b})\big\rangle$ in mode *B*_*i*_.
2. **Measurement**: (Same as Step 2 in Box 1.) Alice and Bob send modes *A*_*i*_ and *B*_*i*_ to Charlie, who performs the single-photon interference measurement. Charlie announces the clicks of the detectors *L* and/or *R*. Alice and Bob repeat the above two steps *N* times; then, they perform the following data postprocessing procedures:
3. **Mode pairing**: (Same as Step 3 in Box 1.) For all rounds with successful detection (*L* or *R* clicks), Alice and Bob establish a strategy for grouping two clicked rounds as a pair. A specific pairing strategy is introduced in Box 2.
4. **Basis sifting**: Based on the intensities of two grouped rounds, Alice labels the ‘basis’ of the data pair as:
  - *Z* if one of the intensities is 0 and the other is nonzero;
  - *X* if both of the intensities are the same and nonzero; or
  - ‘0’ if the intensities are (0, 0), which will be reserved for decoy estimation of both the *Z* and *X* bases; or
  - ‘discard’ when both intensities are nonzero and not equal.
  See also Table 2 for the basis assignment. Alice and Bob announce the basis (*X*, *Z*, ‘0’, or ‘discard’) and the sum of the intensities $({\mu }_{i,j}^{a},{\mu }_{i,j}^{b})$ for each location pair *i*, *j*. If the announced bases are the same and no ‘discard’ state occurs, they record the pair basis and maintain the data pairs; if one of the announced bases is ‘0’ and the other one is *X*(*Z*), they record the pair basis as *X*(*Z*) and keep the data pairs; if both of the announced bases are ‘0’, they record the pair basis as ‘0’ and maintain the data pairs; and otherwise, they discard the data. See also Table 3 for the basis-sifting strategy.

**Table 2:** Alice’s (or Bob’s) basis assignment on the paired locations *i* and *j*.

	*μ*_*i*_			
		0	*ν*	*μ*
*μ*_*j*_				
0		‘0’	*Z*	*Z*
*ν*		*Z*	*X*	‘discard’
*μ*		*Z*	‘discard’	*X*

Based on the intensities *μ*_*i*_ and *μ*_*j*_ on the *i*-th and *j*-th location, Alice (or Bob) assign the basis to be either *X*, *Z*, ‘0’, or ‘discard’.

**Table 3:** Alice and Bob’s basis sifting procedure on the paired locations *i* and *j*.

	Alice			
		‘0’	*X*	*Z*
Bob				
‘0’		‘0’	*X*	*Z*
*X*		*X*	*X*	‘discard’
*Z*		*Z*	‘discard’	*Z* (key generation)

Based on the assigned basis, Alice and Bob decide whether to keep the data for *Z*-basis key generation, *Z*(*X*)-basis parameter estimation, or discard the data.
5. **Key mapping**: (Same as Step 5 in Box 1) For each *Z*-pair at locations *i* and *j*, Alice sets her key to *κ*^*a*^ = 0 if the intensity of the *i*-th pulse is ${\mu }_{i}^{a}=0$ and to *κ*^*a*^ = 1 if ${\mu }_{j}^{a}=0$. For each *X*-pair at locations *i* and *j*, the key is extracted from the relative phase $({\phi }_{j}^{a}-{\phi }_{i}^{a})={\theta }^{a}+\pi {\kappa }^{a}$, where the raw key bit is ${\kappa }^{a}=\big\lfloor (({\phi }_{j}^{a}-{\phi }_{i}^{a})/\pi {{{{{{{\rm{mod}}}}}}}}2)\big\rfloor$ and the alignment angle is ${\theta }^{a}:= ({\phi }_{j}^{a}-{\phi }_{i}^{a}){{{{{{{\rm{mod}}}}}}}}\pi$. Similarly, Bob also assigns his raw key bit *κ*^*b*^ and determines *θ*^*b*^. For the *X*-pairs, Alice and Bob announce the alignment angles *θ*^*a*^ and *θ*^*b*^. If *θ*^*a*^ = *θ*^*b*^, they keep the data pairs; otherwise, they discard them.
6. **Parameter estimation**: Alice and Bob estimate the quantum bit error rate ${E}_{\mu \mu }^{Z}$ of the raw key data in *Z*-pairs with overall intensities of $({\mu }_{i,j}^{a},{\mu }_{i,j}^{b})=(\mu ,\mu )$. They use *Z*-pairs with different intensity settings to estimate the clicked single-photon fraction *q*_11_ using the decoy-state method, and the *X*-pairs are used to estimate the single-photon phase error rate ${e}_{11}^{X}$. Specially, *q*_11_ and ${e}_{11}^{X}$ are estimated via the decoy-state method introduced in Supplementary Note 3.
7. **Key distillation**: (Same as Step 7 in Box 1.) Alice and Bob use the *Z*-pairs to generate a key. They perform error correction and privacy amplification in accordance with *q*_11_, ${E}_{\mu \mu }^{Z}$ and ${e}_{11}^{X}$.

### Mode-pairing-efficiency calculation

We calculate the expected pairing number *r*_*p*_(*p*, *l*) that corresponds to the simple mode-pairing strategy in Box 2, which is related to the average click probability *p* during each round, and the maximal pairing interval *l*.

For calculation convenience, we assume that in addition to the front and rear locations (*F*_*k*_, *R*_*k*_) of the *k*-th pair, Alice and Bob also record the starting location *S*_*k*_, which indicates the location at which the first successful detection signal occurs during the pairing procedure for the *k*-th pair. If the second successful detection signal *R*_*k*_ is found within the next *l* locations, then *F*_*k*_ = *S*_*k*_; otherwise, *F*_*k*_ will be larger than *S*_*k*_. Let *G*_*k*_ ≔ *S*_*k*+1_ − *S*_*k*_ denote a random variable that reflects the location gap between the *k*-th and (*k* + 1)-th starting pulses. Then the expected pairing number per pulse is given by

12 $${r}_{p}=\frac{1}{{\mathbb{E}}({G}_{k})}.$$

Hence, we need to calculate only the expectation value of *G*_*k*_. First, we split it into two parts,

13 $${G}_{k}=({R}_{k}-{S}_{k})+({S}_{k+1}-{R}_{k})={H}_{k}+{G}_{k}^{(b)},$$

where *H*_*k*_: = *R*_*k*_ − *S*_*k*_ and ${G}_{k}^{(b)}:= {S}_{k+1}-{R}_{k}$. Hence,

14 $${\mathbb{E}}({G}_{k})={\mathbb{E}}({H}_{k})+{\mathbb{E}}({G}_{k}^{(b)}).$$

It is easy to show that ${G}_{k}^{(b)}$ obeys a geometric distribution,

15 $$\Pr ({G}_{k}^{(b)}=d)={(1-p)}^{d-1}p,\,\,\,d=1,2,...$$

Then, the expectation value is ${\mathbb{E}}({G}_{k}^{(b)})=1/p$.

The calculation of the pulse interval *H*_*k*_ is more complex. Suppose that we already know the expectation value ${\mathbb{E}}({H}_{k})$; now we calculate the expectation value ${\mathbb{E}}({H}_{k}| d)$ conditioned on the distance between the starting point and the following click. We have

16 $${\mathbb{E}}({H}_{k}| d)=\left\{\begin{array}{ll}d, \hfill &d\le l,\\ {\mathbb{E}}({H}_{k})+d,&d \; > \; l.\end{array}\right.$$

Therefore,

17 $${\mathbb{E}}({H}_{k})=	\mathop{\sum }\limits_{d=1}^{+\infty }\Pr (d){\mathbb{E}}({H}_{k}| d)\\ =	\mathop{\sum }\limits_{d=1}^{l}{(1-p)}^{d-1}pd+\mathop{\sum}\limits_{d > l}{(1-p)}^{d-1}p[{\mathbb{E}}({H}_{k})+d]\\ =	\mathop{\sum }\limits_{d=1}^{+\infty }{(1-p)}^{d-1}pd+{\mathbb{E}}({H}_{k})\mathop{\sum}\limits_{d > l}{(1-p)}^{d-1}p\\ =	\frac{1}{p}+{\mathbb{E}}({H}_{k}){(1-p)}^{l}$$

We have

18 $${\mathbb{E}}({H}_{k})=\frac{1}{p[1-{(1-p)}^{l}]};$$

therefore,

19 $${\mathbb{E}}({G}_{k}) =\frac{1}{p[1-{(1-p)}^{l}]}+\frac{1}{p},\\ \Rightarrow {r}_{p} ={\left[\frac{1}{p[1-{(1-p)}^{l}]}+\frac{1}{p}\right]}^{-1}.$$

### Note added to proof

After we submitted our work for reviewing, we became aware of a relevant work by Xie et al.^59^, who consider a similar MDI-QKD protocol that match the clicked data to generate key information. Under the assumption that the single-photon distributions in all the Charlie’s successful detection events are independent and identically distributed, the authors simulate the performance of the protocol and show its ability to break the repeaterless rate-transmittance bound.

## Supplementary information

Supplementary Information

## Data Availability

The methods to generate the data in the plots are provided in Supplementary Information. The data that support the plots within this paper and other findings of this study are available from the corresponding authors upon reasonable request.

## Code availability

The detailed simulation methods for the plots are provided in Supplementary Information. The specific code that support the plots within this paper and other findings of this study are available from the corresponding authors upon reasonable request.
