# Supplementary material for: Mode-pairing quantum key distribution
Source: Nat Commun. 2022 Jul 7;13:3903. doi: 10.1038/s41467-022-31534-7 (PMC9262923; doi:10.1038/s41467-022-31534-7)
Supplement: Supplementary file 1 — Supplementary Information [file 41467_2022_31534_MOESM1_ESM.pdf]

Supplementary Information: Mode-pairing quantum key distribution

We present a full security proof of the mode-pairing measurement-device-independent quantum key distribution scheme (MP scheme for simplicity), the details of the numerical simulation and a proof-of-principle experimental demonstration to show the feasibility of the MP scheme. In Supplementary Note 1, we introduce a source-replacement procedure of the random phases and photon numbers in the coherent states as a preliminary. In Supplementary Note 2, we provide the security proof of the MP scheme. In Supplementary Note 3, we introduce the decoy-state estimation method for the mode-pairing scheme introduce in Methods "Mode-pairing scheme with decoy states". In Supplementary Note 4, we list the simulation formulas of the MP scheme, as well as the two-mode measurement-device-independent quantum key distribution, decoy-state BB84, phase-matching quantum key distribution and sending-or-not-sending twin-field quantum key distribution schemes. In Supplementary Note 5, we present numerical results related to the optical pulse intensities of different schemes. In Supplementary Note 6, we present a proof-of-principle demonstration of the mode-pairing scheme without global phase locking.

CONTENTS

Supplementary Note 1. Source replacement of the random phase systems 2
A. Single-optical-mode case 2
B. Two-optical-mode case 3

Supplementary Note 2. Security of mode-pairing scheme 6
A. Single-photon two-mode MDI-QKD 8
B. Coherent-state two-mode MDI-QKD 10
C. Fixed-pairing MP scheme 16
D. Free-pairing MP scheme 23
E. Security analysis with decoy-state estimation 27
F. Prepare-and-measure MP scheme 28

Supplementary Note 3. Decoy-state estimation 31

Supplementary Note 4. Simulation formulas 35
A. Mode-pairing scheme 35
B. Time-bin MDI-QKD 38
C. Other QKD schemes 39

Supplementary Note 5. Additional numerical results 40

Supplementary Note 6. Proof-of principle experimental demonstration 42

References 45

Supplementary Note 1. SOURCE REPLACEMENT OF THE RANDOM PHASE SYSTEMS

In the security proof of the coherent-state QKD schemes, we will frequently come across the photon-number measurement on the coherent states with randomised phases. Using the overall photon-number measurement on two optical modes, one can post-select the single-photon component as an ideal encoding qubit. A unique feature of the MP scheme is that, Alice and Bob do not want to decide which two optical modes to perform the overall photon-number measurement before obtaining the detection result from the untrusted party, Charlie. To analyze the security of the MP scheme in this scenario, we perform an extra source-replacement procedure by introducing extra ancillary systems to record the random phase or photon number information. In this way, we can realize the overall photon-number measurement of the coherent states *indirectly* based on the measurement on the ancillary systems.

For the convenience of later discussion, here, we introduce the source-replacement procedure of the random phases in detail. First, we consider the source replacement of the encoded random phase in a single optical mode in Supplementary Note 1 A. With the Fourier transform on the ancillary basis, we then show the complementarity between encoded phases and photon numbers. After that, we describe the source replacement of the encoded random phases in the two-optical-mode case in Supplementary Note 1 B. We will show the compatibility of the relative-phase measurement and the overall photon-number measurement.

A. Single-optical-mode case

Consider a coherent state $|\sqrt{\mu}e^{i\phi}\rangle$ on system A with a random phase ϕ. For the convenience of later discussion, we assume that the phase ϕ is discretely randomised from the set $\{\phi_j = \frac{2\pi}{D}j\}_{j\in[D]}$. Here, D is the number of discrete phases and $[D] := \{0, 1, ..., D-1\}$. In the source replacement, we introduce a qudit with $d = D$ to record the random phase information. The joint purified system can be written as

$$|\psi\rangle_{\tilde{A}A} = \frac{1}{\sqrt{D}}\sum_{j=0}^{D-1} |j\rangle_{\tilde{A}} |\sqrt{\mu}e^{i\phi_j}\rangle_A . \tag{1}$$

This purified state can be prepared by first preparing a coherent state $|\sqrt{\mu}\rangle_A$ and a maximal coherent qudit state

$$|+_D\rangle_{\tilde{A}} = \frac{1}{\sqrt{D}}\sum_{j=0}^{D-1} |j\rangle_{\tilde{A}} , \tag{2}$$

and then applying a controlled-phase gate C_D-$\hat{U}(\phi_\Delta)$ with $\phi_\Delta := \frac{2\pi}{D}$ from the qudit $\tilde{A}$ to the optical mode A. The controlled-phase gate is defined as

$$C_D\text{-}\hat{U}(\phi)_{\tilde{A}A} := \sum_{j=0}^{D-1} |j\rangle_{\tilde{A}} \langle j| \otimes e^{i\phi j a^\dagger a}, \tag{3}$$

where $a^\dagger$ and a are the creation and annihilation operators of the mode A, respectively.

To define the measurement on the ancillary $\tilde{A}$ that informs the photon number of A, we introduce a complementary basis $\{|\tilde{k}\rangle_{\tilde{A}}\}_{k=0}^{D-1}$ on the qudit system $\tilde{A}$ via the Fourier transform, $\forall k, j \in [D]$,

$$\begin{aligned} |\tilde{k}\rangle_{\tilde{A}} &:= \frac{1}{\sqrt{D}}\sum_{j=0}^{D-1} e^{i\frac{2\pi}{D}jk} |j\rangle_{\tilde{A}} , \\ |j\rangle_{\tilde{A}} &= \frac{1}{\sqrt{D}}\sum_{k=0}^{D-1} e^{-i\frac{2\pi}{D}jk} |\tilde{k}\rangle_{\tilde{A}} . \end{aligned} \tag{4}$$

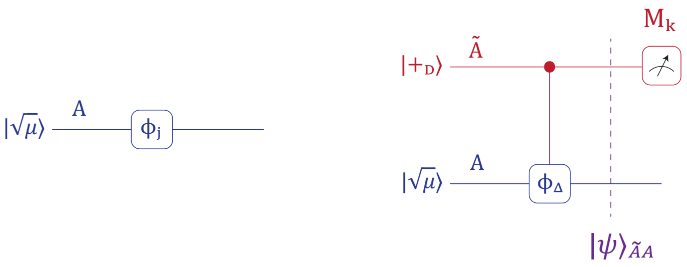

(a) (b)

Supplementary Figure 1. (a) A coherent state with a discrete random phase ϕ_j. (b) A possible way to produce the entangled state generated by the source replacement on the coherent states with random phase encoding. Alice first prepares a maximal coherent qudit state $|+_D\rangle$ on the system $\tilde{A}$, and then performs the control gate $C_D - \hat{U}(\phi_\Delta)$ from $\tilde{A}$ to A to generate the state $|\psi\rangle$. Alice can then choose to measure on $\tilde{A}$ on $\{|j\rangle\}_j$ or $\{|\tilde{k}\rangle\}_k$ basis to read out either the phase or the photon number information of the system A. Here, $\phi_\Delta = \frac{2\pi}{D}$.

In this way, the state in Eq. (1) can be reformulated as

$$\begin{aligned} |\psi\rangle_{\tilde{A}A} &= \frac{1}{\sqrt{D}} \sum_{j=0}^{D-1} |j\rangle_{\tilde{A}} |\sqrt{\mu}e^{i\phi_j}\rangle_A \\ &= \frac{1}{D} \sum_{j=0}^{D-1} \sum_{k=0}^{D-1} e^{-i\frac{2\pi}{D}jk} |\tilde{k}\rangle_{\tilde{A}} |\sqrt{\mu}e^{i\phi_j}\rangle_A \\ &= \frac{1}{D} \sum_{k=0}^{D-1} |\tilde{k}\rangle_{\tilde{A}} \left(\sum_{j=0}^{D-1} e^{-i\frac{2\pi}{D}jk} |\sqrt{\mu}e^{i\phi_j}\rangle_A \right) \\ &= \sum_{k=0}^{D-1} \sqrt{P_k} |\tilde{k}\rangle_{\tilde{A}} |\lambda^\mu(k)\rangle_A , \end{aligned} \tag{5}$$

where the normalized pseudo-Fock state is given by,

$$\begin{aligned} |\lambda^\mu(k)\rangle_A &:= \frac{1}{D\sqrt{P_k^\mu}} \sum_{j=0}^{D-1} e^{-i\frac{2\pi}{D}jk} |\sqrt{\mu}e^{i\phi_j}\rangle_A \\ &= \frac{1}{\sqrt{P_k^\mu}} e^{-\frac{\mu}{2}} \sum_{m=0}^{\infty} \frac{\sqrt{\mu}^{(mD+k)}}{\sqrt{(mD+k)!}} |mD+k\rangle_A , \end{aligned} \tag{6}$$

and the corresponding probability distribution is,

$$P_k^\mu := e^{-\mu} \sum_{m=0}^{\infty} \frac{\mu^{(mD+k)}}{(mD+k)!}. \tag{7}$$

From Eq. (5) we can see that, the global phase ϕ_j and the (pesudo) photon number k are two complementary observables, which cannot be determined simultaneously. Furthermore, since the normalized pesudo-Fock states $\{|\lambda^\mu(k)\rangle_A\}_k$ are orthogonal with each other, if we measure the ancillary system $\tilde{A}$ on the basis $\{|\tilde{k}\rangle\}$, it is equivalent to perform the projective (pesudo) photon-number measurement on the system A.

When $D \to \infty$, Eq. (6) becomes Fock states and Eq. (7) becomes a Poisson distribution, $P^\mu(k) = e^{-\mu}\frac{\mu^k}{k!}$, and then the discrete phase randomisation approaches the continuous phase randomisation. In practice, it suffices to choose $D \geq 12$ to make the discretisation effect ignorable [1, 2].

B. Two-optical-mode case

In the coherent-state MDI-QKD scheme, usually we encode the information into the single-photon subspace on two orthogonal optical modes. To see how this works, we consider the two-optical-mode encoding and show the

compatibility between the global photon number and the relative-phase measurement.

Consider two coherent states $|\sqrt{\mu}e^{i\phi_1}\rangle_{A_1}$ and $|\sqrt{\mu}e^{i\phi_2}\rangle_{A_2}$ on two orthogonal optical modes with random phases ϕ_1 and ϕ_2. Similarly, we assume the random phases are discretely randomised from the sets $\{\phi_{j_1} = \frac{2\pi}{D} j_1\}_{j_1=0}^{D-1}$ and $\{\phi_{j_2} = \frac{2\pi}{D} j_2\}_{j_2=0}^{D-1}$, respectively. The joint purified system can be written as,

$$\begin{aligned} |\psi_{1,2}\rangle_{\tilde{A}_1\tilde{A}_2,A_1A_2} &= \frac{1}{D}\sum_{j_1=0}^{D-1}\sum_{j_2=0}^{D-1} |j_1\rangle_{\tilde{A}_1} |j_2\rangle_{\tilde{A}_2} |\sqrt{\mu}e^{i\phi_{j_1}}\rangle_{A_1} |\sqrt{\mu}e^{i\phi_{j_2}}\rangle_{A_2} \\ &= \frac{1}{D}\sum_{j_2=0}^{D-1}\sum_{j_\theta=0}^{D-1} |j_2+j_\theta\rangle_{\tilde{A}_1} |j_2\rangle_{\tilde{A}_2} |\sqrt{\mu}e^{i\phi_{(j_2+j_\theta)}}\rangle_{A_1} |\sqrt{\mu}e^{i\phi_{j_2}}\rangle_{A_2}, \end{aligned} \tag{8}$$

where we substitute the variable $j_1 := j_2 + j_\theta$. The addition $+$ for all the discrete indices (j_1, j_2 and j_θ) is defined on the ring $\mathbb{Z}_D$ (taking modulus of D).

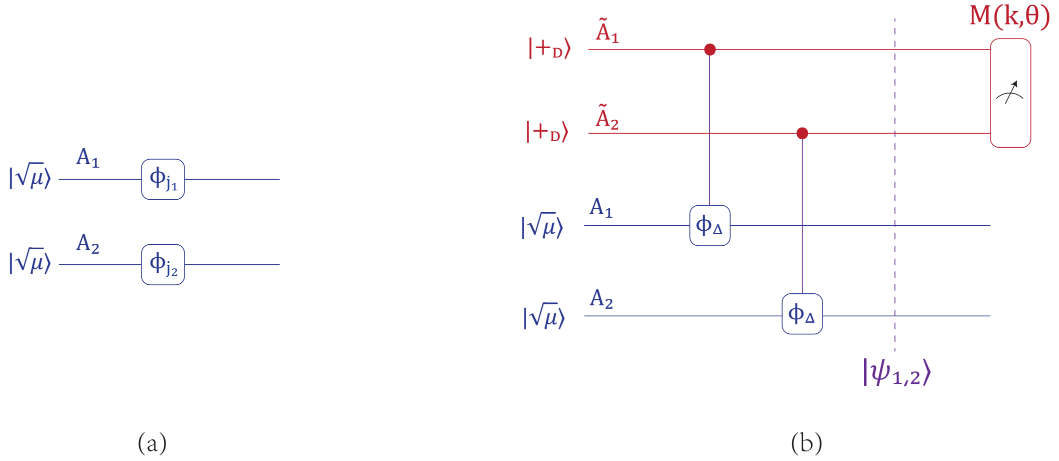

Supplementary Figure 2. (a) Two coherent states with independent random phases ϕ_{j_1} and ϕ_{j_2}. (b) A possible way to produce the entangled state generated by the source replacement on the coherent states with random phase encoding. Alice first prepares two maximal coherent qudit state $|+_D\rangle$ on the systems $\tilde{A}_1$ and $\tilde{A}_2$, then performs the control gates $C_D - \hat{U}(\phi_\Delta)$ from $\tilde{A}_1$ and $\tilde{A}_2$ to A_1 and A_2, respectively, to generate the state $|\psi_{1,2}\rangle$. When Alice performs the joint measurement $M(k,\theta)$ defined in Eq. (13) on $\tilde{A}_1$ and $\tilde{A}_2$, she can obtain the global photon number k as well as the relative phase information θ on systems A_1 and A_2.

For each given θ, the basis vectors $\{|j_2+j_\theta\rangle_{\tilde{A}_1} |j_2\rangle_{\tilde{A}_2}\}_{j_2=0}^{D-1}$ defines a subspace on the joint Hilbert space of two qudits $\tilde{A}_1$ and $\tilde{A}_2$. Similar to Eq. (4), we introduce a partial Fourier transform on each subspace, $\forall k, j_2 \in [D]$,

$$\begin{aligned} |\tilde{k}, j_\theta\rangle_{\tilde{A}_1,\tilde{A}_2} &:= \frac{1}{\sqrt{D}}\sum_{j_2=0}^{D-1} e^{i\frac{2\pi}{D}j_2 k} |j_2+j_\theta\rangle_{\tilde{A}_1} |j_2\rangle_{\tilde{A}_2}, \\ |j_2+j_\theta\rangle_{\tilde{A}_1} |j_2\rangle_{\tilde{A}_2} &= \frac{1}{\sqrt{D}}\sum_{k=0}^{D-1} e^{-i\frac{2\pi}{D}j_2 k} |\tilde{k}, j_\theta\rangle_{\tilde{A}_1\tilde{A}_2}. \end{aligned} \tag{9}$$

In this way, the state in Eq. (8) can be reformulated as

$$
\begin{aligned}
\left|\psi_{1,2}\right\rangle_{\tilde{A}_1\tilde{A}_2,A_1A_2} &= \frac{1}{D}\sum_{j_2=0}^{D-1}\sum_{j_\theta=0}^{D-1}\left|j_2+j_\theta\right\rangle_{\tilde{A}_1}\left|j_2\right\rangle_{\tilde{A}_2}\left|\sqrt{\mu}e^{i\phi_{(j_2+j_\theta)}}\right\rangle_{A_1}\left|\sqrt{\mu}e^{i\phi_{j_2}}\right\rangle_{A_2} \\
&= \frac{1}{D\sqrt{D}}\sum_{j_2=0}^{D-1}\sum_{j_\theta=0}^{D-1}\sum_{k=0}^{D-1}e^{-i\frac{2\pi}{D}j_2k}\left|\tilde{k},j_\theta\right\rangle_{\tilde{A}_1,\tilde{A}_2}\left|\sqrt{\mu}e^{i\phi_{(j_2+j_\theta)}}\right\rangle_{A_1}\left|\sqrt{\mu}e^{i\phi_{j_2}}\right\rangle_{A_2} \\
&= \frac{1}{D\sqrt{D}}\sum_{j_\theta=0}^{D-1}\sum_{k=0}^{D-1}\left|\tilde{k},j_\theta\right\rangle_{\tilde{A}_1\tilde{A}_2}\left(\sum_{j_2=0}^{D-1}e^{-i\frac{2\pi}{D}j_2k}\left|\sqrt{\mu}e^{i\phi_{(j_2+j_\theta)}}\right\rangle_{A_1}\left|\sqrt{\mu}e^{i\phi_{j_2}}\right\rangle_{A_2}\right) \\
&= \frac{1}{D\sqrt{D}}\sum_{j_\theta=0}^{D-1}\sum_{k=0}^{D-1}\left|\tilde{k},j_\theta\right\rangle_{\tilde{A}_1\tilde{A}_2}\ \hat{U}_{A_1}(\frac{2\pi}{D}j_\theta)\circ\hat{BS}\left(\sum_{j_2=0}^{D-1}e^{-i\frac{2\pi}{D}j_2k}\left|\sqrt{2\mu}e^{i\phi_{j_2}}\right\rangle_{A_1}\left|0\right\rangle_{A_2}\right) \\
&= \sum_{j_\theta=0}^{D-1}\frac{1}{\sqrt{D}}\sum_{k=0}^{D-1}\sqrt{P_k^{2\mu}}\left|\tilde{k},j_\theta\right\rangle_{\tilde{A}_1\tilde{A}_2}\ \left|\lambda_{1,2}^{2\mu}(k),j_\theta\right\rangle_{A_1A_2},
\end{aligned}
\tag{10}
$$

where in the second equality, we use the partial Fourier transform in Eq. (9). In the fourth equality, we simplify the expression by a phase-gate $\hat{U}_{A_1}(\phi) := e^{i\phi a_1^\dagger a_1}$ and the 50:50 beam-splitter $\hat{BS}$ with the following transformation relationship,

$$
\begin{pmatrix} a_1' \\ a_2' \end{pmatrix} = \frac{1}{\sqrt{2}}\begin{pmatrix} 1 & 1 \\ 1 & -1 \end{pmatrix}\begin{pmatrix} a_1 \\ a_2 \end{pmatrix},
\tag{11}
$$

where a_1 and a_2 denote the annihilation operators of the two input modes, while a_1' and a_2' denote the annihilation operators of the two output modes. In the fifth equality, we define

$$
\begin{aligned}
\left|\lambda_{1,2}^{2\mu}(k),j_\theta\right\rangle_{A_1A_2} :&= \frac{1}{D\sqrt{P_k^{2\mu}}}\hat{U}_{A_1}(\frac{2\pi}{D}j_\theta)\circ\hat{BS}\left(\sum_{j_2=0}^{D-1}e^{-i\frac{2\pi}{D}j_2k}\left|\sqrt{2\mu}e^{i\phi_{j_2}}\right\rangle_{A_1}\left|0\right\rangle_{A_2}\right) \\
&= \hat{U}_{A_1}(\frac{2\pi}{D}j_\theta)\circ\hat{BS}\left(\left|\lambda^{2\mu}(k)\right\rangle_{A_1}\left|0\right\rangle_{A_2}\right) \\
&= \hat{U}_{A_1}(\frac{2\pi}{D}j_\theta)\circ\hat{BS}\left(\frac{e^{-\mu}}{\sqrt{P_k^{2\mu}}}\sum_{m=0}^{\infty}\frac{\sqrt{2\mu}^{(mD+k)}}{\sqrt{(mD+k)!}}\left|mD+k\right\rangle_{A_1}\left|0\right\rangle_{A_2}\right),
\end{aligned}
\tag{12}
$$

to be a normalized state with pseudo-Fock number k and relative phase $\theta = \frac{2\pi}{D}j_\theta$.

From Eq. (10) we can see that, the overall (pseudo) photon number k and the (discrete) relative phase θ are two *compatible* observables for two coherent states with random phases. Denote the projective measurement on the basis $\{\left|\tilde{k},j_\theta\right\rangle_{\tilde{A}_1,\tilde{A}_2}\}$ to be a global photon-number and encoded relative-phase measurement $M(k,\theta)$,

$$
M(k,\theta) := \left\{\Pi_{k,j_\theta} := \left|\tilde{k},j_\theta\right\rangle_{\tilde{A}_1,\tilde{A}_2}\left\langle\tilde{k},j_\theta\right|\right\},
\tag{13}
$$

which will be frequently used in the following security proof.

It is easy to check that, the conditional states $\{\left|\lambda_{1,2}^{2\mu}(k),j_\theta\right\rangle\}_{k,j_\theta}$ with different photon numbers k are orthogonal with each other. We now consider the following two encoding procedures,

1. Alice first prepares an entangled state $\left|\psi_{1,2}\right\rangle_{\tilde{A}_1\tilde{A}_2,A_1A_2}$. She them performs the measurement $M(k,\theta)$ defined in Eq. (13) on $\tilde{A}_1$ and $\tilde{A}_2$ to determine the global photon number k and the encoded relative phase $\theta = j_\theta\frac{2\pi}{D}$ on the emitted optical modes A_1 and A_2;

2. Alice generates two coherent states on A_1 and A_2 with random phases ϕ_{j_1} and ϕ_{j_2}, respectively. She records the relative phase $\theta = j_\theta\frac{2\pi}{D}$ and then performs direct global photon-number measurement on the two coherent states to determine the global photon number k.

In Ref. [1], it has been shown that the resultant states in the second procedure are also $\{|\lambda_{1,2}^{2\mu}(k), j_\theta\rangle\}_{k,j_\theta}$. As a result, these two encoding procedures are equivalent.

In the extreme case when $D \to \infty$, the state $|\lambda_{1,2}^{2\mu}(k), j_\theta\rangle_{A_1A_2}$ becomes

$$|\lambda_{1,2}(k), j_\theta\rangle = \hat{U}_{A_1}(\frac{2\pi}{D} j_\theta) \circ \hat{BS}\left(|k\rangle_{A_1}|0\rangle_{A_2}\right), \tag{14}$$

which is independent of the intensity μ. Especially, the state with global photon number $k = 1$ and the relative phase ϕ_{j_θ} becomes

$$|\lambda_{1,2}(1), j_\theta\rangle = \frac{1}{\sqrt{2}}(|01\rangle + e^{i\frac{2\pi}{D}j_\theta}|10\rangle)_{A_1A_2}, \tag{15}$$

which forms a qubit subspace widely used in QKD.

Supplementary Note 2. SECURITY OF MODE-PAIRING SCHEME

In this section, we prove the security of the MP scheme via proving the security of its equivalent entanglement version. The major tool we use in the security proof is the QKD equivalence argument.

For a generic MDI-QKD setting, Alice and Bob can view Charlie's site as a joint measurement, M_c, on emitted optical pulses. Here, measurement M_c contains all Charlie's operations, including measurement on emitted optical pulses, data processing, and the announcement strategy. The measurement-device-independent property makes measurement M_c a black box to Alice and Bob. Based on the measurement result, $\vec{C}$, Alice and Bob perform a key-generation operation, M_{ab}, to their ancillary states to get the final private states, where the key-generation operation M_{ab} includes state measurement, key mapping, parameter estimation, and data post-processing. The whole procedure is depicted in Supplementary Figure 3.

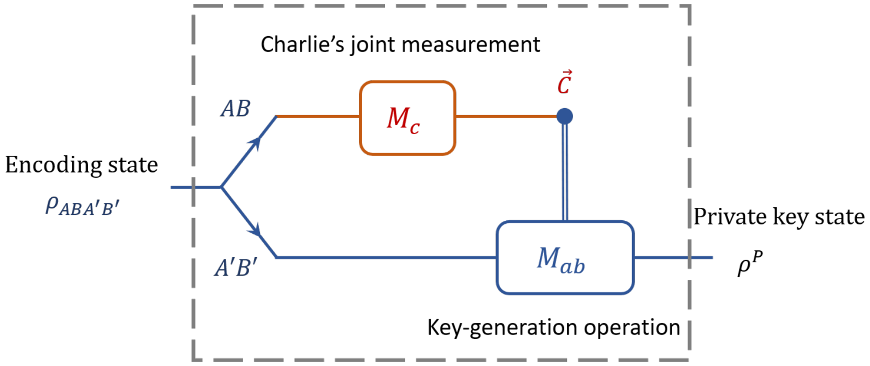

Supplementary Figure 3. Generic MDI-QKD. The red part owned by Charlie is uncharacterized and untrusted. The blue part is well characterized in Alice and Bob's hands. In general, Charlie's joint measurement outcome, $\vec{C}$, controls Alice and Bob's key-generation operation, M_{ab}. This (classical) control operation is fixed by the protocol. That is, given a measurement result, $\vec{C}$, Alice and Bob know exactly how to operate on their ancillary states to extract the final key. We can treat the whole circuit in the gray dashed box as a gigantic operation on the initial state, $\rho_{ABA'B'}$, to output a private key state, ρ^P.

Lemma 1 (Equivalent MDI-QKD)**.** *Two MDI-QKD schemes, in the form of Supplementary Figure 3, generate the same private state given the same attack and hence are equivalent in security, if the following items are the same,*

1. *Alice and Bob's initial states, including emitted quantum states of system AB and ancillary states of system $A'B'$;*

2. *the (classical) control operation for key generation, $\vec{C}$-M_{ab}.*

Here, item 2 implies the dependence of Alice and Bob's key-generation operation M_{ab} shown in Supplementary Figure 3 are the same in the two schemes if Charlie's announcements $\vec{C}$ are the same.

Proof. Since Alice and Bob's initial states in both schemes are the same, the spaces of possible operations M_c Charlie can perform on system AB are the same. Then, we only need to compare the resultant private key states, ρ^P, when Charlie performs the same operations M_c on the two schemes.

In Supplementary Figure 3, we can treat the whole circuit as one gigantic operation, as shown in the gray dashed box. If Charlie's operations M_c are the same in the two schemes, the final private states are the same, because they both come from the same operation on the same state. In MDI-QKD, an eavesdropper's attack is reflected in M_c. Given any attack, the two schemes would render the same private key states. Therefore, the security of the two schemes are equivalent in security. □

Corollary 1 (Equivalent MDI-QKD under post-selection)**.** *Two MDI-QKD schemes, in the form of Supplementary Figure 3, are equivalent in security, if the following items are the same,*

1. *Alice and Bob's initial states, including emitted quantum states of system AB and ancillary states of system $A'B'$, after a post-selection procedure that is independent of Charlie's announcement $\vec{C}$;*

2. *the (classical) control operation for key generation $\vec{C}$-M_{ab}.*

The Corollary 1 is a direct result of Lemma 1. Based on Corollary 1, we can introduce extra encoding redundancy in MDI-QKD. If the encoding state of the new MDI-QKD scheme is the same as the original one with proper post-selection independent of Charlie's announcement $\vec{C}$, then the security of the new scheme is equivalent to the original one.

The main procedure to prove the security of the mode-pairing scheme is to reduce the entanglement-based MP scheme to the traditional single-photon MDI-QKD scheme through a few source-replacement steps, as sketched out in Supplementary Figure 4. For the security proof, we work our way backward from the final stage to the first one. For the completeness of the analysis, we will also review the well-established security proof of the single-photon two-mode MDI-QKD and the coherent-state two-mode MDI-QKD schemes with the source-replacement language.

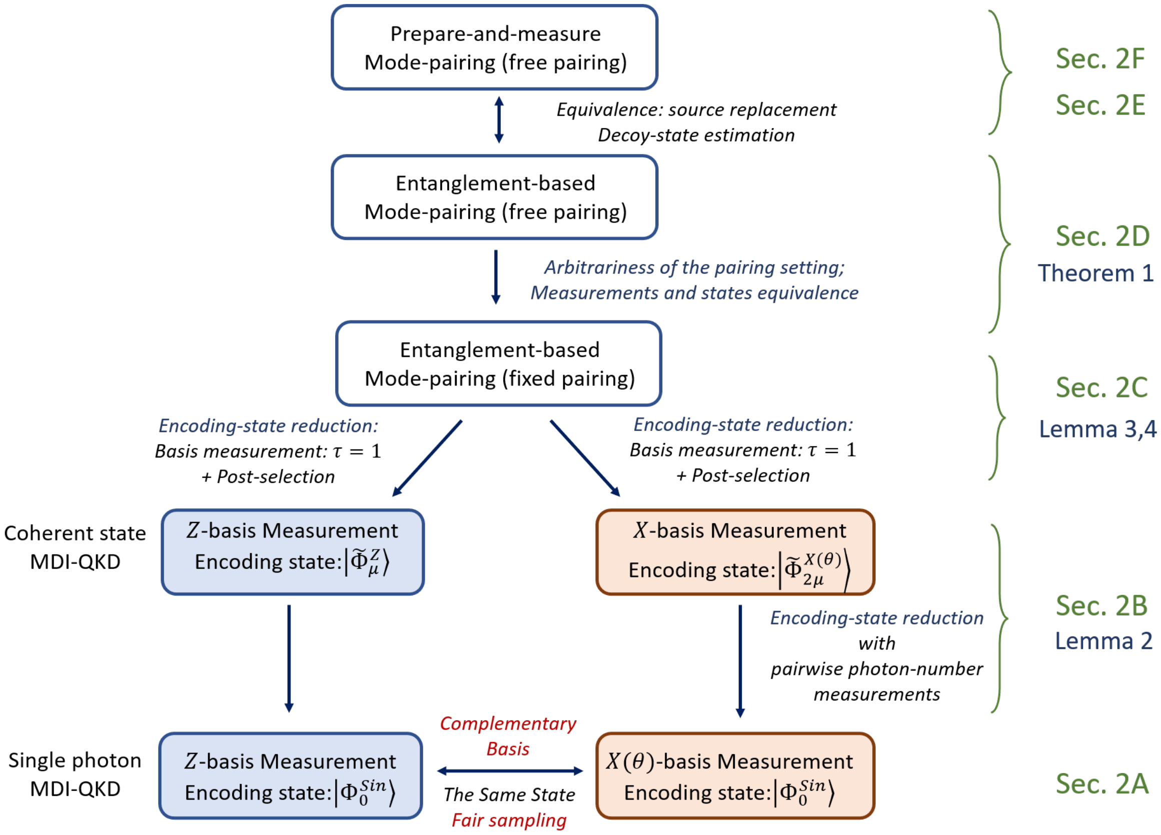

Supplementary Figure 4. Sketch of the security proof of the MP scheme. The main idea is to introduce source replacement and then reduce the security of the MP scheme to that of single-photon MDI-QKD, where the X-basis error rate can be used to fairly estimate the Z-basis phase-error rate.

In Supplementary Note 2 A, we review an single-photon two-mode MDI-QKD scheme whose security can be easily verified by the Lo-Chau security proof based on entanglement distillation [3].

In Supplementary Note 2 B, we review coherent-state two-mode MDI-QKD. Based on the methods in Supplementary Note 1, we replace the random phase systems with ancillary qudits. By introducing overall photon-number measurement, we can reduce the encoding state to the single-photon MDI-QKD case reviewed in Supplementary Note 2 A.

In Supplementary Note 2 C, we introduce a mode-pairing scheme with a fixed pairing setting, where the prepared states in different rounds are identical and independently distributed (i.i.d.). We then reduce the MP scheme to the two-mode MDI-QKD scheme. To do so, we perform global control gates on the ancillary qubits and measure them to assign the bases, denoted by τ, and perform post-selection. We show that the encoding states of the MP scheme with proper post-selection will be the same as those of the two-mode MDI-QKD scheme.

In Supplementary Note 2 D, we consider the MP scheme in which the pairing setting is not predetermined, but rather is determined by Charlie's announcements. We will show that when choosing a pairing setting based on Charlie's announcements, the free-pairing MP scheme is equivalent to the fixed-pairing MP scheme with the same pairing setting. The arbitrariness of the pairing setting gives us the freedom to choose pairing strategies, which can even be determined by Charlie.

In Supplementary Note 2 E, we discuss the security of the MP schemes without actual photon-number measurement, which is the case for the prepare-and-measure one. We will see that, following similar proof methods in Supplementary Note 2 C and Supplementary Note 2 D, we introduce decoy-state estimation in the MP schemes and prove the security.

Finally, in Supplementary Note 2 F, we reduce the entanglement-based scheme to the prepare-and-measure one in the main text via the Shor-Preskill argument [4].

A. Single-photon two-mode MDI-QKD

We start with the case where Alice and Bob both hold single-photon sources. The diagram of a two-mode MDI-QKD scheme is shown in Supplementary Figure 5 [5, 6]. Alice holds an ancillary qubit system A' that interacts with two optical modes, A_1 and A_2. The single-photon subspace of the two modes forms a qubit. Bob's encoding and post-selection procedures are the same as those of Alice unless otherwise stated. The encoding process is shown in Supplementary Figure 6. Note that the encoding methods (a) and (b) in Supplementary Figure 6 produce the same state ρ_0.

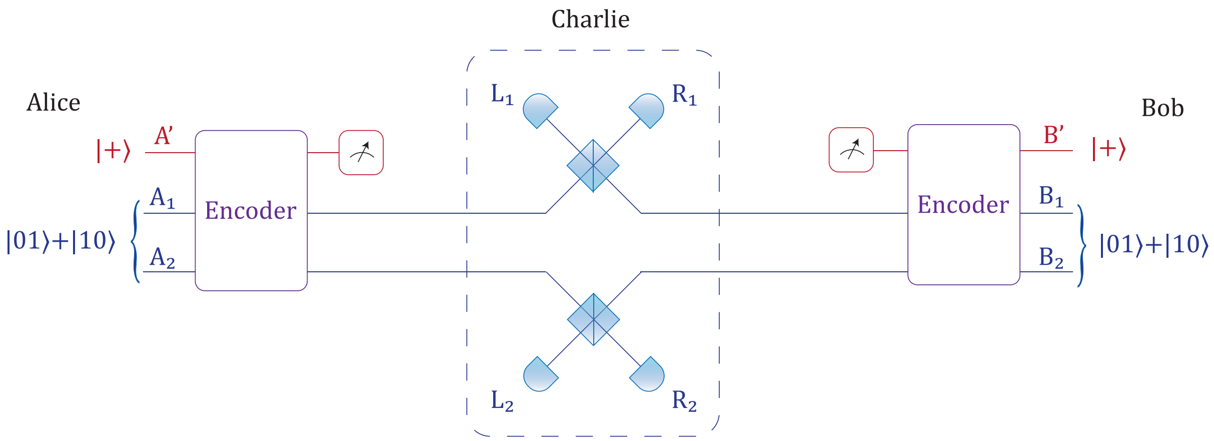

Supplementary Figure 5. Diagram of the entanglement version of the two-mode MDI-QKD scheme when Alice and Bob hold single photon sources. The red lines represent the ancillary qubits used by Alice and Bob to store key information and distill keys, while the blue lines represent the optical modes transmitted to Charlie.

In the two-mode MDI-QKD scheme, Alice and Bob each emit encoded signals to a measurement device controlled by Charlie in the middle, who is supposed to correlate their emitted signals. Alice (Bob) uses a single photon on two orthogonal modes $A_1(B_1)$ and $A_2(B_2)$ as a qubit. The Z basis of the qubit is naturally defined as

$$
\begin{aligned}
|\psi_z(0)\rangle &= |1\rangle_{A_1}|0\rangle_{A_2}, \\
|\psi_z(1)\rangle &= |0\rangle_{A_1}|1\rangle_{A_2}.
\end{aligned}
\tag{16}
$$

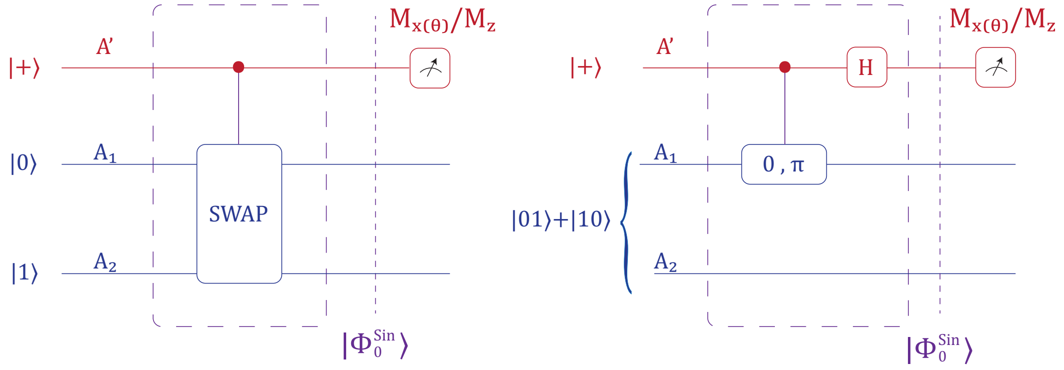

(a) (b)

Supplementary Figure 6. Alice's encoding process in the entanglement version of the two-mode MDI-QKD scheme. (a) Alice uses an ancillary state $|+\rangle$ to perform a control-swap operation on two modes A_1 and A_2. (b) Alice first generates a single-photon superposition state $\frac{1}{\sqrt{2}}(|01\rangle + |10\rangle)$ on two modes, A_1 and A_2; then, she uses an ancillary qubit A' to control the phase of A_1. Note that the encoding processes described in (a) and (b) produce exactly the same state, $|\Phi_0^{Sin}\rangle$. Hereafter, in all the figures, the red line denotes the qubit ancillaries, while the blue line denotes the optical modes.

For simplification, we omit the tensor notation $\otimes$ between different modes unless any ambiguity occurs. The $\{X(\theta)\}_\theta$-basis is defined as

$$
\begin{aligned}
|+(\theta)\rangle &= \frac{1}{\sqrt{2}}(|\psi_z(0)\rangle + e^{-i\theta}|\psi_z(1)\rangle) = \frac{1}{\sqrt{2}}(|10\rangle + e^{-i\theta}|01\rangle)_{A_1,A_2}, \\
|-(\theta)\rangle &= \frac{1}{\sqrt{2}}(|\psi_z(0)\rangle - e^{-i\theta}|\psi_z(1)\rangle) = \frac{1}{\sqrt{2}}(|10\rangle - e^{-i\theta}|01\rangle)_{A_1,A_2},
\end{aligned}
\tag{17}
$$

where $\theta \in [0, \pi)$. When $\theta = 0$ and $\pi/2$, these become the eigenstates of the X and Y bases, respectively. A state on the X-Y plane can then be denoted by

$$
|\psi_x^\theta(\kappa)\rangle = \frac{1}{\sqrt{2}}(|\psi_z(0)\rangle + e^{-i(\theta+\pi\kappa)}|\psi_z(1)\rangle), \tag{18}
$$

where θ denotes the basis and κ denotes the sign of the state. The basis defined by $\{|\psi_x^\theta(\kappa)\rangle\}_{\kappa=0,1}$ is called the $X(\theta)$-basis.

In Supplementary Figure 6, the generated encoding state is

$$
|\Phi_0^{Sin}\rangle = \frac{1}{\sqrt{2}}(|0\rangle|01\rangle + |1\rangle|10\rangle)_{A';A_1,A_2}, \tag{19}
$$

where the superscript Sin indicates that the state is of a single photon. If we regard the single-photon as a qubit, then $|\Phi_0^{Sin}\rangle$ is the Bell state.

For the qubit system A', we can similarly define the $X(\theta)$-basis using Eq. (18). To realise an $X(\theta)$-basis measurement on A', one first performs a Z-axis rotation $R_Z(\theta)$ on A' before performing X-basis measurement,

$$
|\Phi_\theta^{Sin}\rangle := R_Z(\theta)|\Phi_0^{Sin}\rangle = \frac{1}{\sqrt{2}}(|0\rangle|01\rangle + e^{i\theta}|1\rangle|10\rangle)_{A';A_1,A_2}. \tag{20}
$$

Therefore, as shown in Supplementary Figure 7(c), an $X(\theta)$-basis measurement on A' can be equivalently realised by first modulating the phase of A_1 by θ and then measuring A' on the X-basis. We also remark that a Z-basis measurement on $|\Phi_\theta^{Sin}\rangle$ is equivalent to one on $|\Phi_0^{Sin}\rangle$.

Hereafter, we establish the Z basis as for key generation and the $X(\theta)$ basis only for parameter estimation. The single-photon two-mode MDI-QKD with a fixed θ runs as shown in Box 1.

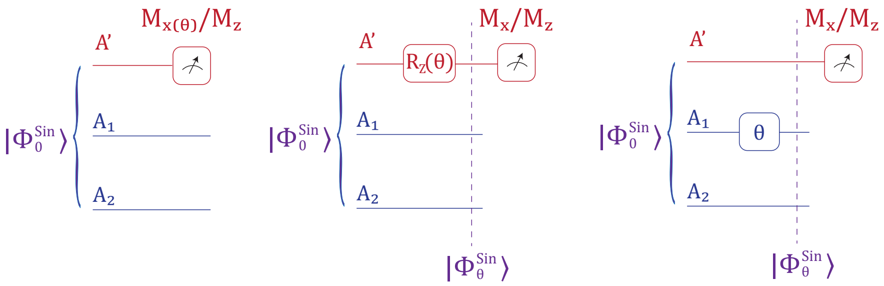

(a) (b) (c)

Supplementary Figure 7. Equivalent realisation of the encoding process and measurement in Supplementary Figure 6. The Z-axis rotation on qubit A' can be moved to a phase modulation on mode A_1. The resultant state $|\Phi_\theta^{Sin}\rangle$ remains invariant. Here, the red gate $R_Z(\theta)$ denotes a Z-axis rotation by an angle θ, while the blue gate θ denotes phase modulation on mode A_1.

Box 1: Single-photon two-mode MDI-QKD

1. State preparation: Alice prepares an entangled state $|\Phi_0^{Sin}\rangle$ defined in Eq. (19) with devices (a) or (b) described in Supplementary Figure 6, containing a qubit system A' and two optical modes, A_1 and A_2. Similarly, Bob prepares ρ_0 on B', B_1 and B_2.

2. Measurement: Alice and Bob send their optical modes A_1, A_2, B_1 and B_2 to an untrusted party, Charlie, who is supposed to perform coincident interference measurement, as shown in Supplementary Figure 5.

3. Announcement: Charlie announces the L_1, R_1, L_2 and R_2 detection results. If one of L_1 and R_1 clicks and one of L_2 and R_2 clicks, then Alice and Bob keep their signals. If it is (L_1, R_2)-click or (L_2, R_1)-click, then Bob applies Z gate on his qubit B'.

   Alice and Bob perform the above steps over many rounds and end up with a joint $2n$-qubit state $\rho_{A'B'} \in (\mathcal{H}_{A'} \otimes \mathcal{H}_{B'})^{\otimes n}$.

4. Parameter estimation: Alice decides at random whether to perform measurements in the Z or $X(\theta)$ basis and announces her basis choice to Bob. They then measure A', B' in the same basis. They announce the $X(\theta)$-basis measurement results and estimate the (phase) error rate. The Z-basis measurement results on $\rho_{A'B'}$ are denoted by the raw-data string κ^a and κ^b.

5. Classical post-processing: Alice and Bob reconcile the key string to κ^a via an classical channel by consuming l_{ec} key bits. They then perform privacy amplification using universal-2 hashing matrices. The sizes of the hashing matrices are determined by the estimated phase-error rate of κ^a from the $X(\theta)$-basis error rate.

The security of this single-photon MDI-QKD can be reduced to that of the BBM92 scheme [7]. Following the security proof based on complementarity [3, 4, 8], we must estimate the phase-error rate, i.e., the information disturbance in the complementary basis of the Z basis for key generation. Since the single-photon source is basis-independent, one can fairly estimate the phase-error rate of the Z basis using the $X(\theta)$ basis.

In the classical post-processing, the information reconciliation is conducted by an encrypted classical channel with the consummation of a preshared l_{ec}-bit key. This is for the convenience of the description of the security analysis. In practice, one can apply different ways of one-way information reconciliation without encryption. The key rate of the single-photon MDI-QKD under the one-way classical communication will be the same.

B. Coherent-state two-mode MDI-QKD

In practice, the users can replace single-photon sources with weak coherent-state sources. The security of coherent-state MDI-QKD schemes have already been well studied in previous MDI-QKD works [5, 6], where a photon-number-channel model is assumed. That is, the untrusted Charlie first measures the global photon number k^a and k^b on Alice's and Bob's emitted optical modes, respectively. Based on the measurement outcomes, Charlie then decides the

follow-up measurement and announcement strategy.

Here, we prove the security of coherent-state two-mode MDI-QKD from a new perspective where the random phases are purified and stored locally, as mentioned in Supplementary Note 1 B. In this way, Alice and Bob can decide and perform the photon-number measurement on the optical modes after emitting the signals to Charlie.

In the original coherent-state two-mode MDI-QKD scheme, Alice first generates two random phases, ϕ_1^a and ϕ_2^a, which are independently and uniformly chosen from $[0, 2\pi)$, and then performs the encoding process shown in Supplementary Figure 8. The Z-basis encoding state is

$$|\Phi_\mu^Z(\phi_1^a, \phi_2^a)\rangle = \frac{1}{\sqrt{2}}\left(|0\rangle|0\rangle|\sqrt{\mu}e^{i\phi_2^a}\rangle + |1\rangle|\sqrt{\mu}e^{i\phi_1^a}\rangle|0\rangle\right)_{A';A_1;A_2}. \tag{21}$$

We use the notation $\theta^a := \phi_1^a - \phi_2^a$. The state can also be written as

$$|\Phi_\mu^Z(\theta^a, \phi_2^a)\rangle = \frac{1}{\sqrt{2}}\left(|0\rangle|0\rangle|\sqrt{\mu}e^{i\phi_2^a}\rangle + |1\rangle|\sqrt{\mu}e^{i(\phi_2^a+\theta^a)}\rangle|0\rangle\right)_{A';A_1;A_2}. \tag{22}$$

If we randomise the phase ϕ_2^a uniformly in $[0, 2\pi)$ and keep θ^a fixed, then the single-photon part of the resultant state is $|\Phi_{\theta^a}^{Sin}\rangle$ in Eq. (20),

$$|\Phi_{\theta^a}^{Sin}\rangle = R_Z(\theta^a)|\Phi_0^{Sin}\rangle = \frac{1}{\sqrt{2}}(|0\rangle|01\rangle + e^{i\theta^a}|1\rangle|10\rangle)_{A';A_1,A_2}. \tag{23}$$

In what follows, the coherent state with complex amplitude $\sqrt{\mu}e^{i\phi}$ will always be written in this form $|\sqrt{\mu}e^{i\phi}\rangle$ to avoid the ambiguity to the Fock states $|0\rangle, |1\rangle, ..., |k\rangle$.

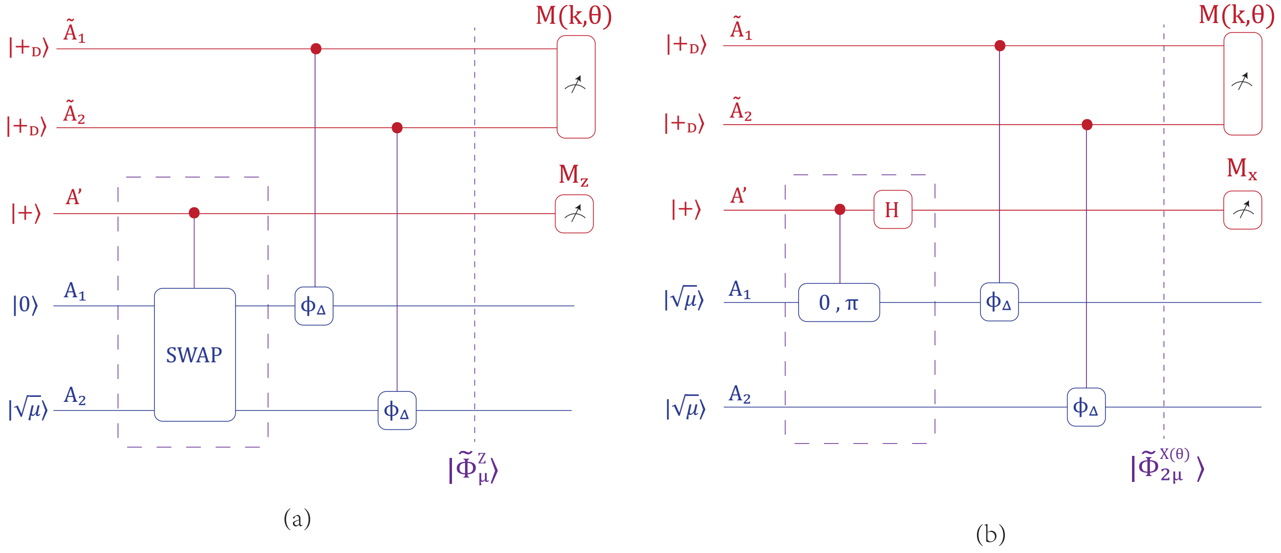

Supplementary Figure 8. Alice's encoding in two-mode MDI-QKD with a coherent-state source of intensity μ. Alice generates the Z-basis state $|\tilde{\Phi}_\mu^Z\rangle$ or $X(\theta)$-basis encoding state $|\tilde{\Phi}_0^{X(\theta^a)}\rangle$ with two qudit systems $\tilde{A}_1$ and $\tilde{A}_2$ to store the random phase information ϕ_1^a and ϕ_2^a, respecively. The qubit system A' is used for the final key generation. Here, $\theta^a := \phi_1^a - \phi_2^a$. (a) and (b) present the Z- and $X(\theta)$-basis encoding processes, respectively. The state $|+_D\rangle := \frac{1}{\sqrt{D}}\sum_{m=0}^{D-1}|m\rangle$. When Alice performs the global measurements $M(k, \theta)$ defined in Eq. (13) on the systems $\tilde{A}_1$ and $\tilde{A}_2$ and the photon number $k = 1$ and relative phase is θ^a, the post-selected state on the systems A', A_1 and A_2 is approximately the single-photon-encoding state $|\Phi_{\theta^a}^{Sin}\rangle$ for both Z-basis and $X(\theta)$-basis case. In this case, the encoding processes in (a) and (b) will be reduced to those in (a) and (b) of Supplementary Figure 6.

Recall that the Z-basis measurement result on the state $|\Phi_{\theta^a}^{Sin}\rangle$ is independent of the relative phase θ^a. For any fixed θ^a, an $X(\theta)$-basis measurement on $|\Phi_\mu^Z(\theta^a, \phi_2^a)\rangle$ is equivalent to an $X(\theta + \theta^a)$-basis measurement on $|\Phi_\mu^Z(0, \phi_2^a)\rangle$. Hereafter, we do not discriminate between encoded states with different θ^a for the Z basis.

Now, we further perform the source replacement for the encoded random phases ϕ_1^a and ϕ_2^a, following the methods used in Supplementary Note 1. To do this, we first assume the random phases ϕ_1^a and ϕ_2^a are *discretely and uniformly randomised* from the set $\{\frac{2\pi}{D}j\}_{j\in[D]}$. Here D is the number of discrete phases. Note that picking up a $D \geq 10$ would

make the discrete phase randomization very close to the continuous one [1]. Then, we introduce two ancillary qudit systems $\tilde{A}_1$ and $\tilde{A}_2$ with $d = D$ to store the random phase information, as shown in Supplementary Figure 2(b).

The whole Z-basis encoding state with the purified random phase system is

$$|\tilde{\Phi}_\mu^Z\rangle = \frac{1}{\sqrt{2}D}\sum_{j_1=0}^{D-1}\sum_{j_2=0}^{D-1}|j_1\rangle_{\tilde{A}_1}|j_2\rangle_{\tilde{A}_2}\left(|0\rangle|0\rangle|\sqrt{\mu}e^{i\phi_{j_2}}\rangle + |1\rangle|\sqrt{\mu}e^{i\phi_{j_1}}\rangle|0\rangle\right)_{A',A_1A_2}, \tag{24}$$

where $\phi_{j_1} := \frac{2\pi}{D}j_1$ and $\phi_{j_2} := \frac{2\pi}{D}j_2$ are the two random phases with indices j_1 and j_2, respectively.

The $X(\theta)$-basis encoding state is

$$|\Phi_{2\mu}^{X(\theta)}(\phi_1^a,\phi_2^a)\rangle = \frac{1}{\sqrt{2}}\left(|0\rangle|\sqrt{\mu}e^{i(\phi_1^a)}\rangle|\sqrt{\mu}e^{i\phi_2^a}\rangle + |1\rangle|\sqrt{\mu}e^{i(\phi_1^a+\pi)}\rangle|\sqrt{\mu}e^{i\phi_2^a}\rangle\right)_{A';A_1A_2}. \tag{25}$$

Similar to the Z-basis case, we introduce purified systems $\tilde{A}_1$ and $\tilde{A}_2$ to register the random phase information,

$$|\tilde{\Phi}_{2\mu}^{X(\theta)}\rangle = \frac{1}{\sqrt{2}D}\sum_{j_1=0}^{D-1}\sum_{j_2=0}^{D-1}|j_1\rangle_{\tilde{A}_1}|j_2\rangle_{\tilde{A}_2}\left(|+\rangle|\sqrt{\mu}e^{i\phi_{j_1}}\rangle|\sqrt{\mu}e^{i\phi_{j_2}}\rangle + |-\rangle|\sqrt{\mu}e^{i(\phi_{j_1}+\pi)}\rangle|\sqrt{\mu}e^{i\phi_{j_2}}\rangle\right)_{A';A_1A_2}. \tag{26}$$

To reduce the Z-basis and $X(\theta)$-basis encoding state $|\tilde{\Phi}_\mu^Z\rangle$ and $|\tilde{\Phi}_{2\mu}^{X(\theta)}\rangle$ to the single-photon encoding state $|\Phi_\theta^{Sin}\rangle$ in Eq. (20), we have the following lemma.

Lemma 2 (Encoding-state reduction from coherent-state to single-photon two-mode MDI-QKD)**.** *In coherent-state two-mode MDI-QKD, Alice generates the state* $|\tilde{\Phi}_\mu^Z\rangle$ *defined in Eq.* (24) *or* $|\tilde{\Phi}_{2\mu}^{X(\theta)}\rangle$ *defined in Eq.* (26) *for* Z*-basis and* $X(\theta)$*-basis encoding, respectively. She then performs the global measurement* $M(k,\theta)$ *defined in Eq.* (13) *on the local ancillary qudits* $\tilde{A}_1$ *and* $\tilde{A}_2$*. When the discrete phase number* $D \to \infty$*, we have,*

1. *(Poisson photon-number distribution) For the* Z*-basis state* $|\tilde{\Phi}_\mu^Z\rangle$ *with overall intensity* μ*, the probability of the photon-number measurement result* k *is* $\Pr(k) = e^{-\mu}\frac{\mu^k}{k!}$*; for the* $X(\theta)$*-basis state* $|\tilde{\Phi}_{2\mu}^{X(\theta)}\rangle$ *with overall intensity* 2μ*, the probability of the photon-number measurement result* k *is* $\Pr(k) = e^{-2\mu}\frac{(2\mu)^k}{k!}$*;*

2. *(Independence of the photon-number states to the intensity) The resultant state after the pairwise measurement* $M(k,\theta)$ *is independent of the intensity value* μ*.*

3. *(Basis-independence of the single-photon state) If the measurement result on* $|\tilde{\Phi}_\mu^Z\rangle$ *or* $|\tilde{\Phi}_{2\mu}^{X(\theta)}\rangle$ *is* $k = 1$ *and* $\theta = \frac{2\pi}{D}j_\theta$*, then the conditional state on* A'*,* A_1 *and* A_2 *will be reduced to* $|\Phi_\theta^{Sin}\rangle$ *defined in Eq.* (20)*.*

Proof. In Section Supplementary Note 1 B, we introduce a global basis transformation on the ancillary systems $\tilde{A}_1$ and $\tilde{A}_2$. Assuming all the phases are discretely chosen from the set $\{\frac{2\pi}{D}j\}_{j\in[D]}$, we can express a purified encoded

state Eq. (24) and transform the basis on it,

$$
\begin{aligned}
|\tilde{\Phi}_{\mu}^{Z}\rangle &= \frac{1}{\sqrt{2D}}\sum_{j_1=0}^{D-1}\sum_{j_2=0}^{D-1}|j_1\rangle_{\tilde{A}_1}|j_2\rangle_{\tilde{A}_2}\left(|0\rangle|0\rangle|\sqrt{\mu}e^{i\phi_{j_2}}\rangle+|1\rangle|\sqrt{\mu}e^{i\phi_{j_1}}\rangle|0\rangle\right)_{A',A_1A_2}\\
&= \frac{1}{\sqrt{2D}}\sum_{j_2=0}^{D-1}\sum_{j_\theta=0}^{D-1}|j_2+j_\theta\rangle_{\tilde{A}_1}|j_2\rangle_{\tilde{A}_2}\left(|0\rangle|0\rangle|\sqrt{\mu}e^{i\phi_{j_2}}\rangle+|1\rangle|\sqrt{\mu}e^{i\phi_{j_2+j_\theta}}\rangle|0\rangle\right)_{A',A_1A_2}\\
&= \frac{1}{\sqrt{2D}D}\sum_{j_2=0}^{D-1}\sum_{j_\theta=0}^{D-1}\sum_{k=0}^{D-1}e^{-i\frac{2\pi}{D}j_2k}|\tilde{k},j_\theta\rangle_{\tilde{A}_1,\tilde{A}_2}\left(|0\rangle|0\rangle|\sqrt{\mu}e^{i\phi_{j_2}}\rangle+|1\rangle|\sqrt{\mu}e^{i\phi_{j_2+j_\theta}}\rangle|0\rangle\right)_{A',A_1A_2}\\
&= \frac{1}{\sqrt{2D}D}\sum_{j_\theta=0}^{D-1}\sum_{k=0}^{D-1}|\tilde{k},j_\theta\rangle_{\tilde{A}_1,\tilde{A}_2}\sum_{j_2=0}^{D-1}\left[e^{-i\frac{2\pi}{D}j_2k}\left(|0\rangle|0\rangle|\sqrt{\mu}e^{i\phi_{j_2}}\rangle+|1\rangle|\sqrt{\mu}e^{i\phi_{j_2+j_\theta}}\rangle|0\rangle\right)_{A',A_1A_2}\right]\\
&= \frac{1}{\sqrt{D}D}\sum_{j_\theta=0}^{D-1}\sum_{k=0}^{D-1}|\tilde{k},j_\theta\rangle_{\tilde{A}_1,\tilde{A}_2}\hat{U}_{A_1}(\frac{2\pi}{D}j_\theta)\circ C\text{-}S\hat{W}AP\left(\sum_{j_2=0}^{D-1}e^{-i\frac{2\pi}{D}j_2k}|+\rangle_{A'}|0\rangle_{A_1}|\sqrt{\mu}e^{i\phi_{j_2}}\rangle_{A_2}\right)\\
&= \sum_{j_\theta=0}^{D-1}\frac{1}{\sqrt{D}}\sum_{k=0}^{D-1}\sqrt{P_k^\mu}|\tilde{k},j_\theta\rangle_{\tilde{A}_1,\tilde{A}_2}\hat{U}_{A_1}(\frac{2\pi}{D}j_\theta)\circ C\text{-}S\hat{W}AP\left(|+\rangle_{A'}|0\rangle_{A_1}|\lambda^\mu(k)\rangle_{A_2}\right)\\
&= \sum_{j_\theta=0}^{D-1}\frac{1}{\sqrt{D}}\sum_{k=0}^{D-1}\sqrt{P_k^\mu}|\tilde{k},j_\theta\rangle_{\tilde{A}_1,\tilde{A}_2}|\lambda_Z^\mu(k),j_\theta\rangle_{A',A_1A_2}.
\end{aligned}
\tag{27}
$$

Here, in the third equality, we introduce a partial Fourier transform defined in Eq. (9). In the fifth equality, we simplify the expression by a phase-gate $\hat{U}_{A_1}(\phi) := e^{i\phi a_1^\dagger a_1}$ and a controlled-swap gate $C\text{-}S\hat{W}AP$ defined on a qubit A' and two optical modes,

$$
\begin{aligned}
C\text{-}S\hat{W}AP|0\rangle_{A'}|\psi(a_1^\dagger,a_2^\dagger)\rangle &= |0\rangle_{A'}|\psi(a_1^\dagger,a_2^\dagger)\rangle,\\
C\text{-}S\hat{W}AP|1\rangle_{A'}|\psi(a_1^\dagger,a_2^\dagger)\rangle &= |1\rangle_{A'}|\psi(a_2^\dagger,a_1^\dagger)\rangle.
\end{aligned}
\tag{28}
$$

In the sixth equality, we use the pseudo-Fock state definition in Eq. (6). The probability

$$
P_k^\mu := e^{-\mu}\sum_{m=0}^{\infty}\frac{\mu^{(mD+k)}}{(mD+k)!},
\tag{29}
$$

is a mixture of the Poisson distribution probability $P^\mu(k) := e^{-\mu}\frac{\mu^k}{k!}$. In the seventh equality, the state $|\lambda_Z^\mu(k),j_\theta\rangle_{A,A_1A_2}$ is

$$
\begin{aligned}
|\lambda_Z^\mu(k),j_\theta\rangle_{A,A_1A_2} :&= \hat{U}_{A_1}(\frac{2\pi}{D}j_\theta)\circ C\text{-}S\hat{W}AP\left(|+\rangle_{A'}|0\rangle_{A_1}|\lambda^\mu(k)\rangle_{A_2}\right)\\
&= \hat{U}_{A_1}(\frac{2\pi}{D}j_\theta)\circ C\text{-}S\hat{W}AP\left(|+\rangle_{A'}\frac{e^{-\frac{\mu}{2}}}{\sqrt{P_k^\mu}}\sum_{m=0}^{\infty}\frac{\sqrt{\mu}^{(mD+k)}}{\sqrt{(mD+k)!}}|0\rangle_{A_1}|mD+k\rangle_{A_2}\right),
\end{aligned}
\tag{30}
$$

is a normalized state with pseudo-Fock number k and relative phase $\theta = \frac{2\pi}{D}j_\theta$.

When $D\to\infty$, the probability P_k^μ becomes the Poisson distribution. The conditional encoding state $|\lambda_Z^\mu(k),j_\theta\rangle_{A',A_1A_2}$ becomes

$$
|\lambda_Z^\mu(k),j_\theta\rangle_{A,A_1A_2} = \hat{U}_{A_1}(\frac{2\pi}{D}j_\theta)\circ C\text{-}S\hat{W}AP\left(|+\rangle_{A'}|0\rangle_{A_1}|k\rangle_{A_2}\right),
\tag{31}
$$

which is irrelavant to the intensity μ. Especially, when $k=1$, the conditinal state becomes

$$
|\lambda_Z^\mu(1),j_\theta\rangle_{A,A_1A_2} = \frac{1}{\sqrt{2}}(|0\rangle|01\rangle + e^{i\theta}|1\rangle|10\rangle)_{A,A_1A_2} = |\Phi_\theta^{Sin}\rangle,
\tag{32}
$$

Here, $|\Phi_\theta^{Sin}\rangle$ is defined in Eq. (20). When D is finite, the equality of Eq. (32) becomes approximation due to the discrete phase randomisation effect. It has been shown in the literature [1, 2] that when $D\geq 12$, the discrete phase randomization is very close to the continuous one.

If we perform the same basis transformation on $\tilde{A}_1$ and $\tilde{A}_2$ as the one for the Z-basis state in Eq. (27), the $X(\theta)$-basis encoding state will become

$$\begin{aligned}
|\tilde{\Phi}_{2\mu}^{X(\theta)}\rangle &= \frac{1}{\sqrt{2}D}\sum_{j_\theta=0}^{D-1}\sum_{j_2=0}^{D-1}|j_2+j_\theta\rangle_{\tilde{A}_1}|j_2\rangle_{\tilde{A}_2}\left(|+\rangle|\sqrt{\mu}e^{i\phi_{j_2+j_\theta}}\rangle|\sqrt{\mu}e^{i\phi_{j_2}}\rangle+|-\rangle|\sqrt{\mu}e^{i(\phi_{j_2+j_\theta}+\pi)}\rangle|\sqrt{\mu}e^{i\phi_{j_2}}\rangle\right)_{A',A_1A_2} \\
&= \frac{1}{\sqrt{2}DD}\sum_{j_\theta=0}^{D-1}\sum_{j_2=0}^{D-1}\sum_{k=0}^{D-1}e^{-i\frac{2\pi}{D}j_2k}|\tilde{k},j_\theta\rangle_{\tilde{A}_1,\tilde{A}_2}\left(|+\rangle|\sqrt{\mu}e^{i\phi_{j_2+j_\theta}}\rangle|\sqrt{\mu}e^{i\phi_{j_2}}\rangle+|-\rangle|\sqrt{\mu}e^{i(\phi_{j_2+j_\theta}+\pi)}\rangle|\sqrt{\mu}e^{i\phi_{j_2}}\rangle\right)_{A',A_1A_2} \\
&= \frac{1}{\sqrt{2}DD}\sum_{j_\theta=0}^{D-1}\sum_{k=0}^{D-1}|\tilde{k},j_\theta\rangle_{\tilde{A}_1,\tilde{A}_2}\sum_{j_2=0}^{D-1}\left[e^{-i\frac{2\pi}{D}j_2k}\left(|+\rangle|\sqrt{\mu}e^{i\phi_{j_2+j_\theta}}\rangle|\sqrt{\mu}e^{i\phi_{j_2}}\rangle+|-\rangle|\sqrt{\mu}e^{i(\phi_{j_2+j_\theta}+\pi)}\rangle|\sqrt{\mu}e^{i\phi_{j_2}}\rangle\right)_{A',A_1A_2}\right] \\
&= \frac{1}{\sqrt{D}}\sum_{j_\theta=0}^{D-1}\sum_{k=0}^{D-1}\sqrt{P_k^{2\mu}}|\tilde{k},j_\theta\rangle_{\tilde{A}_1,\tilde{A}_2}\hat{H}_{A'}C\text{-}\hat{U}(\pi)_{A',A_1}\left(|+\rangle_{A'}|\lambda_{1,2}^{2\mu}(k),j_\theta\rangle_{A_1A_2}\right) \\
&= \frac{1}{\sqrt{D}}\sum_{j_\theta=0}^{D-1}\sum_{k=0}^{D-1}\sqrt{P_k^{2\mu}}|\tilde{k},j_\theta\rangle_{\tilde{A}_1,\tilde{A}_2}|\lambda_{X(\theta)}^{2\mu}(k)\rangle_{A',A_1A_2}.
\end{aligned} \tag{33}$$

Here, in the second equality, we use the partial Fourier transform in Eq. (9). In the fourth equality, we simplify the expression with a Hadamard gate $\hat{H}_{A'}$ on the qubit A' and the controlled phase gate $C\text{-}\hat{U}(\phi)_{A',A_1}$,

$$C\text{-}\hat{U}(\phi)_{A',A_1} = |0\rangle_A'\langle 0|\otimes I_{A_1} + |1\rangle_A'\langle 1|\otimes U(\phi)_{A_1}. \tag{34}$$

The state $|\lambda_{1,2}^{2\mu}(k),j_\theta\rangle_{A',A_1A_2}$ is defined in Eq. (12). In the fifth equality, we define

$$|\lambda_{X(\theta)}^{2\mu}(k)\rangle_{A',A_1A_2} := \hat{H}_{A'}C\text{-}\hat{U}(\pi)_{A',A_1}\left(|+\rangle_{A'}|\lambda_{1,2}^{2\mu}(k),j_\theta\rangle_{A_1A_2}\right). \tag{35}$$

If Alice performs the measurement $M(k,\theta)$ defined in Eq. (13) on the ancillary systems $\tilde{A}_1$ and $\tilde{A}_2$ and obtains k and $\theta = \frac{2\pi}{D}j_\theta$, then the conditional emitted state is $|\lambda_{X(\theta)}^{2\mu}(k)\rangle_{A,A_1A_2}$. When $D\to\infty$, the probability $P_k^{2\mu}$ to get the photon-number result k becomes the Poisson distribution. As is shown in Eq. (14), the state $|\lambda_{1,2}^{2\mu}(k),j_\theta\rangle_{A',A_1A_2}$ (and hence the state $|\lambda_{X(\theta)}^{2\mu}(k)\rangle_{A',A_1A_2}$) becomes independent of the intensity μ. Especially, when $k=1$, the conditional state $|\lambda_{X(\theta)}^{2\mu}(k)\rangle_{A',A_1A_2}$ becomes

$$|\lambda_{X(\theta)}^{2\mu}(1)\rangle_{A,A_1A_2} = \frac{1}{2}\left[|+\rangle(|01\rangle+e^{i\theta}|10\rangle)+|-\rangle(|01\rangle-e^{i\theta}|10\rangle)\right]_{A,A_1A_2} = |\Phi_\theta^{Sin}\rangle, \tag{36}$$

where $|\Phi_\theta^{Sin}\rangle$ is defined in Eq. (20). Again, when $D\geq 12$, the approximation caused by discrete (instead of continuous) phase randomization can be ignored. □

As is shown in Eqs. (27) and (33), Alice can obtain the information of the overall photon number k of A_1 and A_2 indirectly based on the collective measurement on the ancillary systems $\tilde{A}_1$ and $\tilde{A}_2$. When the measurement result is $k=1$, both of the Z-basis and $X(\theta)$-basis encoding state will be reduced to the single-photon encoding state $|\Phi_\theta^{Sin}\rangle$ defined in Eq. (20). In this way, the security of the coherent-state MDI-QKD will be reduced to the one of single-photon MDI-QKD.

In the coherent-state two-mode MDI-QKD scheme, Alice and Bob use Z-basis data to generate a secure key and $X(\theta)$-basis data to estimate information leakage, reflected in the $X(\theta)$-basis single-photon error rate $e_{11}^{X(\theta)}$. Here, the relative phase $\theta^a(\theta^b)$ is not fixed, but determined by $\phi_1^a-\phi_2^a$ $(\phi_1^b-\phi_2^b)$, as randomly chosen from $[0,2\pi)$. During the basis-sifting process, Alice and Bob sift the data with $\theta^a-\theta^b=0$ or π to estimate the privacy of raw keys generated in the Z-basis. Here, we mix all $X(\theta)$ data with different θ to estimate the average phase-error rate $\bar{e}_{11}^X$,

$$\bar{e}_{11}^X := \sum_\theta P(\theta)e_{(1,1)}^{X(\theta)}, \tag{37}$$

where $P(\theta)$ is the conditional probability of choosing the alignment angle θ in all of the sifted X-basis data with $\theta^a=\theta^b$ and $e_{(1,1)}^{X(\theta)}$ is the phase-error rate when the alignment angle is $\theta^a=\theta^b=\theta$. This averaged phase-error rate can still faithfully characterise the privacy of Z-basis key generation due to the concavity of the binary entropy function.

From Lemma 2 we can see that, in the coherent-state two-mode MDI-QKD scheme, if Alice and Bob post-select the signals with the overall photon number $k^a = k^b = 1$, then the remaining signals can be used to extract key information similar to the single-photon MDI-QKD scheme in Supplementary Note 2 A. In practice, Alice and Bob do not need to keep track of the photon number data and perform the photon-number post-selection. Instead, they may apply the "tagging" idea [9]: they first keep all the raw-key data generated by different photon numbers k^a and k^b and treat the photon number as a "tag" on the emitted signal; they then estimate the fraction $q_{(1,1)}$ of the single-photon-pair signals with $k^a = k^b = 1$ among all the raw-key data and the $X(\theta)$-basis error rate of the single-photon-pair signals $e^{X(\theta)}_{(1,1)}$. Based on the estimation of $q_{(1,1)}$ and $e^{X(\theta)}_{(1,1)}$, they can also extract the same amount of secure keys by designing proper privacy amplification procedures. The key-rate formula is given by [9]

$$r = q_{(1,1)} \left[1 - H(\bar{e}^{X}_{11})\right] - f Q_{\mu\mu} H(E^{Z}_{(\mu,\mu)}). \tag{38}$$

where $q_{(1,1)}$ is the estimated detected fraction of single-photon components, $H(x)$ is the binary entropy function, and f is a factor that reflects the efficiency of information reconciliation. $Q_{\mu\mu}$ and $E_{\mu\mu}$ are the overall detected fraction and bit-error rate when Alice and Bob emit coherent states with intensity μ. Furthermore, Alice and Bob do not have to measure the photon-number k^a and k^b in each round. Thanks to the photon-number Poisson statistics and the independence of the photon-number state to the intensity setting in Lemma 2, Alice and Bob can vary the intensity μ to estimate the single-photon component in the final detected rounds using decoy-state methods [10].

To summarize, we list the coherent-state two-mode MDI-QKD in Box 2. Here, we asuume that Alice and Bob perform the photon-number measurement, but perform the data post-processing based on the tagging key rate formula in Eq. (38).

Box 2: Coherent-state two-mode MDI-QKD

1. State preparation: Alice decides at random whether to prepare the Z- or $X(\theta)$-basis states. For the Z-basis, Alice prepares an entangled state $|\tilde{\Phi}^{Z}_{\mu}\rangle$ defined in Eq. (24) using the device in Supplementary Figure 8(a), which contains a qubit system A', two ancillary qudit systems $\tilde{A}_1$ and $\tilde{A}_2$, and two optical modes A_1 and A_2. For the $X(\theta)$-basis, she prepares $|\tilde{\Phi}^{X(\theta)}_{2\mu}\rangle$ defined in Eq. (26) by means of Supplementary Figure 8(b). Similarly, Bob prepares $|\tilde{\Phi}^{Z}_{\mu}\rangle$ or $|\tilde{\Phi}^{X(\theta)}_{2\mu}\rangle$ randomly on systems B', $\tilde{B}_1$, $\tilde{B}_2$, B_1 and B_2.

2. Measurement: Alice and Bob send their optical modes A_1, A_2, B_1 and B_2 to an untrusted party, Charlie, who is supposed to perform coincident interference measurement, as shown in Supplementary Figure 5.

3. Announcement: Charlie announces the L_1, R_1, L_2 and R_2 detection results. If one of L_1 and R_1 clicks and one of L_2 and R_2 clicks, Alice and Bob keep the signals. Alice performs $M(k,\theta)$ measurement defined in Eq. (13) on qudits $\tilde{A}_1$ and $\tilde{A}_2$ to obtain the photon number k^a and the relative phase θ^a. Bob operates similarly to obatin k^b and θ^b. If there is an (L_1, R_2)-click or (L_2, R_1)-click, then Bob applies the Z gate to his qubit B'. They announce the measured photon number k^a and k^b.

4. Basis sifting: Alice and Bob announce that the chosen basis is either Z or $X(\theta)$. If the chosen basis is $X(\theta)$, they further announce the relative phases θ^a and θ^b. If they both choose the Z-basis or the $X(\theta)$-basis with $\theta^a - \theta^b = 0/\pi$, they keep their data.

   Alice and Bob perform the above steps over many rounds and end up with a joint $2n$-qubit state $\rho_{A'B'} \in (\mathcal{H}_{A'} \otimes \mathcal{H}_{B'})^{\otimes n}$.

5. Key mapping: Alice and Bob measure the qubit systems A' and B' in the Z or X bases when the predetermined bases are Z or $X(\theta)$, respectively. They record the Z-basis measurement results as the raw-data strings κ^a and κ^b.

6. Parameter estimation: Alice and Bob announce the $X(\theta)$-basis measurement results. Based on the announced information, they then estimate the $X(\theta)$-basis error rate $e^{X(\theta)}_{(1,1)}$ for all the signals with $k^a = k^b = 1$ and the fraction of signals with $k^a = k^b = 1$ in the remained signals $q_{(1,1)}$.

7. Classical post-processing: Alice and Bob reconcile the key string to κ^a and perform privacy amplification using universal-2 hashing matrices. The sizes of hashing matrices are determined by the estimated single-photon-pair fraction $q_{(1,1)}$ and $X(\theta)$-basis error rate $e^{X(\theta)}_{(1,1)}$ according to Eq. (38).

C. Fixed-pairing MP scheme

In this subsection, starting from the coherent-state MDI-QKD scheme, we introduce extra encoding redundancies to construct the mode-pairing (MP) scheme. The major differences of the MP scheme and the coherent-state two-mode MDI-QKD are

1. All the optical modes are decoupled during the encoding process; the correlation among two different rounds i and j are built after the generation of encoding states;
2. Instead of being determined at the beginning, the basis choice for each pair of location is determined by a pairwise measurement on the ancillary systems afterwards.

Unlike the coherent-state MDI-QKD scheme where Alice emits two correlated optical modes in each round, in the MP scheme, Alice first emit optical modes in an i.i.d. manner, then group each two of the sending locations together as a "pair". She then perform collective quantum operations on each pair to extract the correlation information. The concept of "pairing" is formalized below. For the convenience of later discussion, we also define Charlie's announcement.

Definition 1 (Pairing Setting $\vec{\chi}$). *For N location indexes $i = 1, 2, ..., N$ where N is an even number, a pairing setting $\vec{\chi}$ is a set composed of $N/2$ location pairs (i, j) such that all the location indexes appear once and only once in $\vec{\chi}$. The possible pairing settings $\{\vec{\chi}\}$ form a finite set $\mathcal{X}$.*

Definition 2 (Charlie's Announcement $\vec{C}$). *For location indexes $i = 1, 2, ..., N$, denote Charlie's announcement for the i-th round to be $C_i = (L_i, R_i)$, where L_i and R_i are two binary variables indicating the detection results of detectors L and R, respectively. The detection announcement on all the locations can be denoted by a vector $\vec{C}$. The possible Charlie's annoucements $\{\vec{C}\}$ form a finite set $\mathcal{C}$.*

Once the N optical modes are paired, we can denote the location of the k-th pair as (F_k, R_k), where F_k and R_k are the front and rear pulse locations of the k-th pair, respectively. The pairing setting $\vec{\chi}$ can also be viewed as a vector formed by (F_k, R_k). Without loss of generality, we focus on two specific paired locations 1 and 2 in the following discussions.

In the security proof, we first consider a simple MP scheme, where the pairing setting $\vec{\chi}$ is not related to the untrusted Charlie's announcement. We call this MP scheme the fixed-pairing MP scheme.

Definition 3 ($\vec{\chi}$-Fixed-Pairing MP). *In a MP MDI-QKD scheme, if the determination of pairing setting $\vec{\chi}$ is independent of the measurement announcement by the untrusted interferometer, Charlie, then we call such MP scheme the fixed-pairing MP scheme. The fixed-pairing MP scheme with the pairing setting $\vec{\chi}$ is called the $\vec{\chi}$-fixed-pairing MP scheme.*

In the MP scheme, Alice sends out a coherent state with independent intensity and phase modulation in each round,

$$|\psi^{Com}\rangle_{A_1} = |\sqrt{z_1'\mu} e^{i(z_1''\pi + \tilde{z}_1 \frac{2\pi}{D})}\rangle , \tag{39}$$

where z_1' and z_1'' are two random bit values indicating the encoded intensity and $0/\pi$-phase, respectively; $\tilde{z}_1$ is a random dit indicating the discrete random phase. Here, we take the encoding state of the first round, with subscript 1, as the example.

To introduce the entanglement version of the scheme, we perform the source replacement to introduce two ancillary qubits A_1' and A_2'' and an ancillary qudit $\tilde{A}_1$. In this case, Alice will generate the same composite state $|\tilde{\Psi}^{Com}\rangle$ for every round,

$$\begin{aligned} |\tilde{\Psi}^{Com}\rangle_{\tilde{A}_1, A_1', A_1'', A_1} &= \frac{1}{\sqrt{2}} \left(|0\rangle_{A_1'} |\tilde{\Psi}_0^{X(\theta)}\rangle_{\tilde{A}_1, A_1'', A_1} + |1\rangle_{A_1'} |\tilde{\Psi}_\mu^{X(\theta)}\rangle_{\tilde{A}_1, A_1'', A_1} \right) \\ &= \frac{1}{2\sqrt{D}} \sum_{j_1=0}^{D-1} |j_1\rangle_{\tilde{A}_1} \left(|00\rangle |0\rangle + |01\rangle |0\rangle + |10\rangle |\sqrt{\mu} e^{i\phi_{j_1}^a}\rangle + |11\rangle |\sqrt{\mu} e^{i(\phi_{j_1}^a + \pi)}\rangle \right)_{A_1', A_1''; A_1} , \end{aligned} \tag{40}$$

which is composed of an ancillary qudit $\tilde{A}_1$, two ancillary qubits A_1' and A_1'', and an optical mode A_1. In the second equality, we use the following definition of the state $|\tilde{\Psi}_\mu^{X(\theta)}\rangle_{\tilde{A}_1, A_1'', A_1}$

$$|\tilde{\Psi}_\mu^{X(\theta)}\rangle_{\tilde{A}_1, A_1'', A_1} = \frac{1}{\sqrt{2D}} \sum_{j_1=0}^{D-1} |j_1\rangle_{\tilde{A}_1} \left(|0\rangle |\sqrt{\mu} e^{i\phi_{j_1}^a}\rangle + |1\rangle |\sqrt{\mu} e^{i(\phi_{j_1}^a + \pi)}\rangle \right)_{A_1'' A_1} . \tag{41}$$

Interestingly, Alice can post-select the encoding state of coherent-state MDI-QKD by performing some collective operations on two location pairs, as is stated in the following lemma.

Lemma 3 (Encoding-state reduction from the fixed-pairing MP scheme to coherent-state MDI-QKD). *Alice generates two composite states,* $|\tilde{\Psi}^{Com}\rangle_{\tilde{A}_1,A_1',A_1'',A_1}$ *and* $|\tilde{\Psi}^{Com}\rangle_{\tilde{A}_2,A_2',A_2'',A_2}$*, as defined in Eq. (40), on two locations 1 and 2, respectively. Locally, Alice applies a CNOT gate from* A_1' *to* A_2'*, measures* A_2' *on the Z basis, and obtains a result* τ^a*.*

If $\tau^a = 1$*, Alice assigns the Z basis. She then measures* A_1'' *and* A_2'' *on the Z-basis to obtain results* z_1'' *and* z_2''*, respectively. Then, the conditional state in the systems* $\tilde{A}_1$*,* $\tilde{A}_2$*,* A_1'*,* A_1 *and* A_2 *can be reduced to the Z-basis encoding state* $|\tilde{\Phi}_\mu^Z\rangle$ *of Eq. (24) used in the coherent-state MDI-QKD scheme in Supplementary Note 2 A with possible π-phase modulations on the optical modes* A_1 *and* A_2 *when* $z_1'' = 1$ *and* $z_2'' = 1$*, respectively.*

If $\tau^a = 0$*, Alice assigns the* $X(\theta)$ *basis. She first measures* A_1' *on the Z basis to obtain the result* λ^a*. If* $\lambda^a = 0$*, the state will become* $|\tilde{\Phi}_0^{X(\theta)}\rangle$*, which is used for the decoy state estimation. If* $\lambda^a = 1$*, Alice applies a CNOT gate from* A_2'' *to* A_1''*. After that, Alice measures* A_2'' *on the Z basis to obtain the result* ζ^a*. Then the conditional state in the systems* $\tilde{A}_1$*,* $\tilde{A}_2$*,* A_1''*,* A_1 *and* A_2 *can be reduced to the* $X(\theta)$*-basis encoding state* $|\tilde{\Phi}_{2\mu}^{X(\theta)}\rangle$ *of Eq. (26) used in the coherent-state MDI-QKD scheme in Supplementary Note 2 A with a possible simultaneous π-phase modulation on the optical modes* A_1 *and* A_2 *when* $\zeta^a = 1$*.*

Proof. We first summarize the introduced reduction procedure in Supplementary Figure 9, which is independent of Charlie's operation.

To perform the basis-assignment measurement, Alice first performs a CNOT gate on qubits A_1' and A_2' and then measure A_2'. After the CNOT gate, the joint state on $A_1''A_1'A_1$ and $A_2''A_2'A_2$ becomes

$$\begin{aligned}|\tilde{\Psi}_{1,2}^{Com}\rangle =& \frac{1}{2}\big(|00\rangle |\tilde{\Psi}_0^{X(\theta)}\rangle |\tilde{\Psi}_0^{X(\theta)}\rangle + |01\rangle |\tilde{\Psi}_0^{X(\theta)}\rangle |\tilde{\Psi}_\mu^{X(\theta)}\rangle \\ &+ |11\rangle |\tilde{\Psi}_\mu^{X(\theta)}\rangle |\tilde{\Psi}_0^{X(\theta)}\rangle + |10\rangle |\tilde{\Psi}_\mu^{X(\theta)}\rangle |\tilde{\Psi}_\mu^{X(\theta)}\rangle \big)_{A_1',A_2';\tilde{A}_1,A_1'',A_1;\tilde{A}_2,A_2'',A_2},\end{aligned} \tag{42}$$

where $|\tilde{\Psi}_\mu^{X(\theta)}\rangle_{\tilde{A}_1A_1''A_1}$ is defined in Eq. (41). Now, Alice performs Z-basis measurement on the system A_2' to determine the encoding basis.

When the outcome is $\tau^a = 1$, the post-selected state is

$$\begin{aligned}&\frac{1}{\sqrt{2}}\big(|0\rangle |\tilde{\Psi}_0^{X(\theta)}\rangle |\tilde{\Psi}_\mu^{X(\theta)}\rangle + |1\rangle |\tilde{\Psi}_\mu^{X(\theta)}\rangle |\tilde{\Psi}_0^{X(\theta)}\rangle \big)_{A_1';\tilde{A}_1,A_1'',A_1;\tilde{A}_2,A_2'',A_2} \\ =&\frac{1}{2\sqrt{2}D}\sum_{j_1,j_2=0}^{D-1} |j_1\rangle_{\tilde{A}_1} |j_2\rangle_{\tilde{A}_2} \Big[|0\rangle (|0\rangle |0\rangle + |1\rangle |0\rangle) \Big(|0\rangle |\sqrt{\mu}e^{i\phi_{j_2}^a}\rangle + |1\rangle |\sqrt{\mu}e^{i(\phi_{j_2}^a+\pi)}\rangle \Big) \\ &+ |1\rangle \Big(|0\rangle |\sqrt{\mu}e^{i\phi_{j_1}^a}\rangle + |1\rangle |\sqrt{\mu}e^{i(\phi_{j_1}^a+\pi)}\rangle \Big) (|0\rangle |0\rangle + |1\rangle |0\rangle) \Big]_{A_1';A_1'',A_1;A_2'',A_2} \\ =&\frac{1}{2}\Big[|00\rangle |\tilde{\Phi}_\mu^Z\rangle + |01\rangle \hat{U}(\pi)_{A_2} |\tilde{\Phi}_\mu^Z\rangle + |10\rangle \hat{U}(\pi)_{A_1} |\tilde{\Phi}_\mu^Z\rangle + |11\rangle (\hat{U}(\pi)\otimes\hat{U}(\pi))_{A_1,A_2} |\tilde{\Phi}_\mu^Z\rangle \Big]_{A_1';A_1'',A_1;A_2'',A_2},\end{aligned} \tag{43}$$

where $|\tilde{\Phi}_\mu^Z\rangle_{\tilde{A}_1\tilde{A}_2,A_1',A_1A_2}$ is defined in Eq. (24). Now, suppose Alice measures the systems A_1'' and A_2'' on Z-basis and obtains the results z_1'' and z_2''. The post-selected state on the systems A_1', A_1, A_2 will become the Z-basis encoding state $|\tilde{\Phi}_\mu^Z\rangle$ of the coherent-state MDI-QKD scheme with a possible π-phase modulation on the optical modes A_1 and A_2, depending on the values z_1'' and z_2''. This reduction process is illustrated in Supplementary Figure 10.

We remark that, for the single-photon component, a π-phase modulation on the optical modes $\tilde{A}_1$ and $\tilde{A}_2$ can be regarded as a Z-axis rotation on the ancillary qubit A' shown in Supplementary Figure 7. This rotation will not affect the Z-basis measurement result. Therefore, the π-phase modulation on A_1 and A_2 will not affect the security of the Z-basis key generation.

On the other hand, when $\tau^a = 0$, the post-selected state is

$$\frac{1}{\sqrt{2}}\big(|0\rangle |\tilde{\Psi}_0^{X(\theta)}\rangle |\tilde{\Psi}_0^{X}\rangle + |1\rangle |\tilde{\Psi}_\mu^{X(\theta)}\rangle |\tilde{\Psi}_\mu^{X(\theta)}\rangle \big)_{A_1';A_1'',A_1;A_2'',A_2}. \tag{44}$$

In this case, Alice further measures A_1' on the Z basis and keeps her signals if the result is 1. The post-selected state is then $|\tilde{\Psi}_\mu^{X(\theta)}\rangle\otimes|\tilde{\Psi}_\mu^{X(\theta)}\rangle_{\tilde{A}_1A_1''A_1;\tilde{A}_2A_2''A_2}$, where $|\tilde{\Psi}_\mu^{X(\theta)}\rangle$ is defined in Eq. (41). We remark that, if the measurement result on A_1' is 0, then the state will not be used for key generation; instead, it can be used for the parameter estimation.

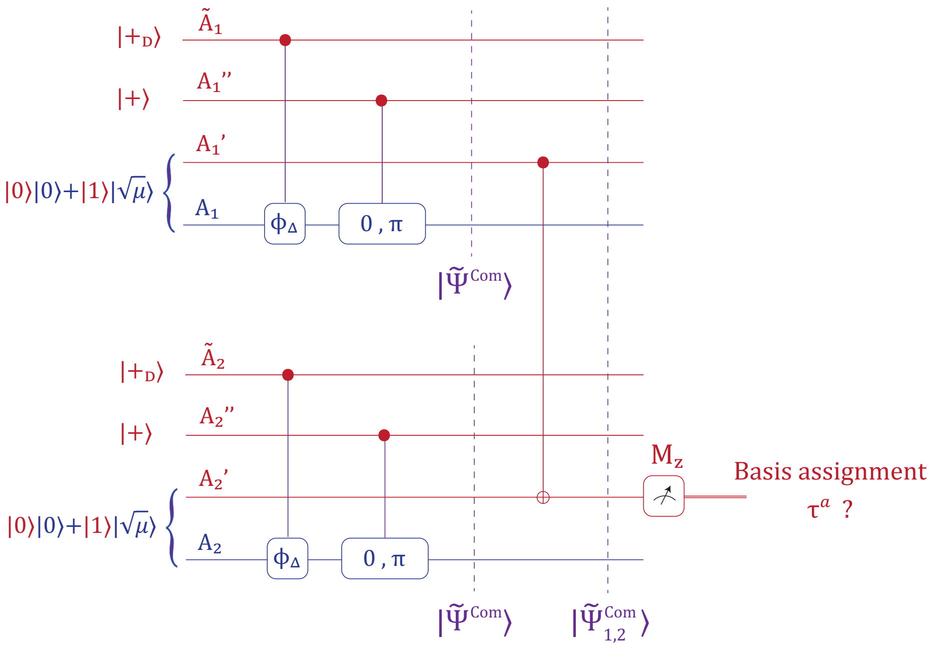

(a)

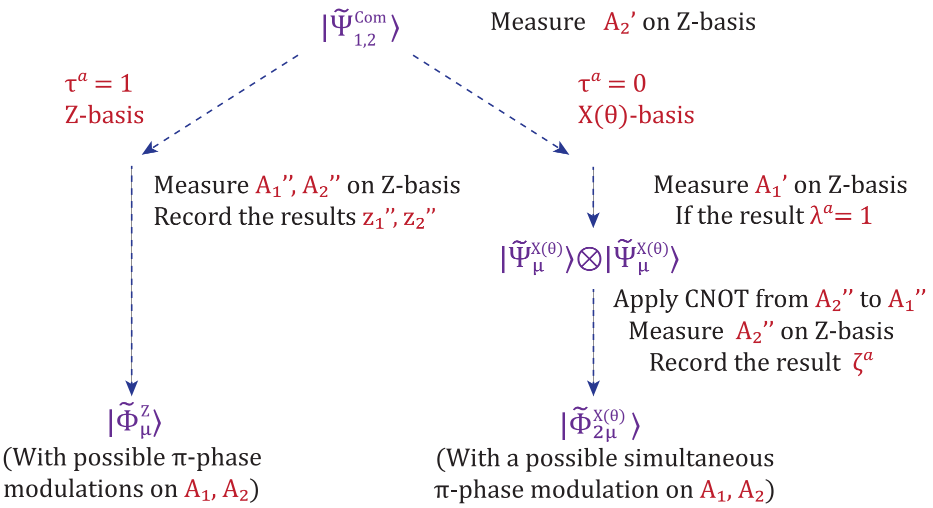

(b)

Supplementary Figure 9. (a) Alice's composite encoding process in the MP scheme. In each round, Alice generates a state $|\tilde{\Psi}^{Com}\rangle$ on the one optical mode A_i, two qubit ancillaries A_i' and A_i'' and a qudit $\tilde{A}_i$ defined in Eq. (40). (here $i=1$ or 2.) She transmits the system A_i to Charlie. Based on the detection, she pairs the ancillary systems based on a pairing setting $\vec{\chi}$. For a pair on locations 1 and 2, she first performs collective detection on systems A_1' and A_2' to determine the basis. (b) The reduction of Alice's state to the Z-basis encoding state $|\tilde{\Phi}^Z_\mu\rangle$ defined in Eq. (24) or the $X(\theta)$-basis encoding state $|\tilde{\Phi}^{X(\theta)}_{2\mu}\rangle$ defined in Eq. (26) of coherent-state MDI-QKD. If the measurement result on A_2' is $\tau^a=1$, Alice measures the modes A_1'' and A_2'' on Z-basis and records the results z_1'' and z_2''. The final state on A_1', A_1, A_2, $\tilde{A}_1$ and $\tilde{A}_2$ will be the Z-basis encoding state $|\tilde{\Phi}^Z_\mu\rangle$ with two possible extra π-phase modulations on A_1 and A_2, based on the values of z_1'' and z_2'', respectively. On the other hand, if $\tau^a=0$, Alice measures A_1' on the Z-basis. If the result is 1, Alice obtains $|\tilde{\Psi}^{X(\theta)}_\mu\rangle\otimes|\tilde{\Psi}^{X(\theta)}_\mu\rangle$ defined in Eq. (41). She applies a CNOT gate from A_2'' to A_1'', measure A_2'' on Z-basis and records the result ζ^a. In this way, she generates $|\tilde{\Phi}^{X(\theta)}_{2\mu}\rangle$ with a possible simultaneous π-phase modulation on A_1 and A_2, based on the value of ζ^a.

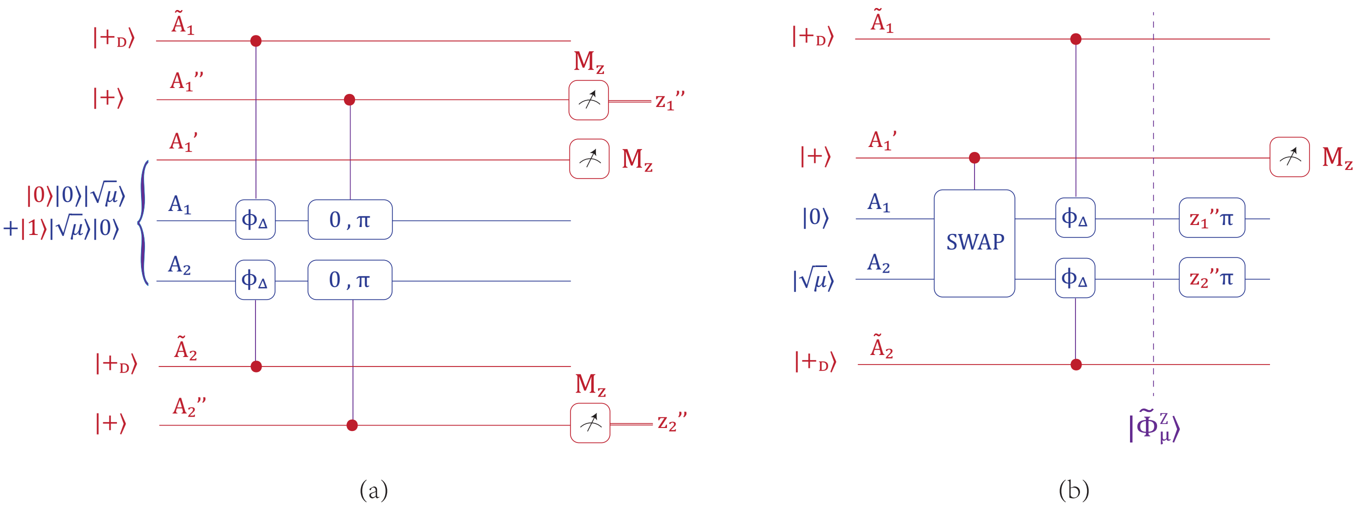

Supplementary Figure 10. Reduction of the encoding state of the MP scheme in Supplementary Figure 9(b) to the Z-basis encoding state $|\tilde{\Phi}_{\mu}^{Z}\rangle$ in Eq. (24) and Supplementary Figure 8(a). After we measure the ancillary qubits A_1'' and A_2'' on Z-basis and obtain the results z_1'' and z_2'', we will project the remaining encoding state to $|\tilde{\Phi}_{\mu}^{Z}\rangle$ with extra phase modulations $z_1''\pi$ and $z_2''\pi$ on the optical modes A_1 and A_2, respectively. The extra π-phase modulation will not affect the security and the performance of the Z-basis coherent-state MDI-QKD scheme.

To reduce the state $|\tilde{\Psi}_{\mu}^{X(\theta)}\rangle \otimes |\tilde{\Psi}_{\mu}^{X(\theta)}\rangle_{\tilde{A}_1 A_1'' A_1;\tilde{A}_2 A_2'' A_2}$ to the $X(\theta)$-basis encoding state $|\tilde{\Phi}_{2\mu}^{X(\theta)}\rangle$ of coherent-state MDI-QKD scheme defined in Eq. (26), Alice applies a CNOT operation from the system A_2'' to A_1'', measures A_2'' on the Z-basis, and records the result ζ^a. As is shown in Supplementary Figure 11, we can equivalently move the Z-basis measurement on A_2'' forward to the beginning. In this way, we can reduce the encoding state to $|\tilde{\Phi}_{2\mu}^{X(\theta)}\rangle$ with a simultaneous π-phase modulation on the optical modes A_1 and A_2. For the $X(\theta)$-basis coherent-state MDI-QKD scheme, such π-phase modulation will not cause any effect.

□

Based on Lemma 3, we introduced the fixed-pairing MP scheme in Box 3, which is also illustrated in Supplementary Figure 12.

Box 3: Fixed-pairing MP scheme

1. State preparation: In each round, Alice prepares the composite state $|\tilde{\Psi}^{Com}\rangle$ defined in Eq. (40) on the optical mode A_i, two ancillary qubits A_i' and A_i'' and an ancillary qudit $\tilde{A}_i$. In a similar manner, Bob prepares $|\tilde{\Psi}^{Com}\rangle$.

2. Measurement: Alice and Bob send modes A_i and B_i to Charlie, who is supposed to perform the single-photon interference measurement. Charlie announces the clicks of the detectors L and/or R.

   Alice and Bob perform the above steps over many rounds. Afterwards, they perform the following data post-processing.

3. Mode Pairing: Based on a pre-set pairing setting $\vec{\chi} \in \mathcal{X}$, Alice and Bob pair their locations (i.e., group each two locations together).

4. Basis Sifting: For each pair on locations i and j, Alice performs the basis-assignment measurement on A_i' and A_j' to determine the basis, as shown in Supplementary Figure 9. If the result $\tau^a = 0$, Alice further measures A_i' on the Z-basis and records the result λ^a. Alice assigns the basis of the pair as Z if $\tau^a = 1$, X if $\tau^a = 0$ and $\lambda^a = 1$ and '0' if $\tau^a = \lambda^a = 0$. Bob assigns the basis in the same way. Alice and Bob announce their bases. If the announced bases are the same, X, X or Z, Z, they keep their signals.

5. Key mapping: For each Z-pair on locations i and j, Alice discards qubits A_i'' and A_j'' and keeps A_i' for later usage. For each X-pair, Alice applies CNOT gate from qubit A_j'' to A_i'', discards qubit A_i'', keeps qubit

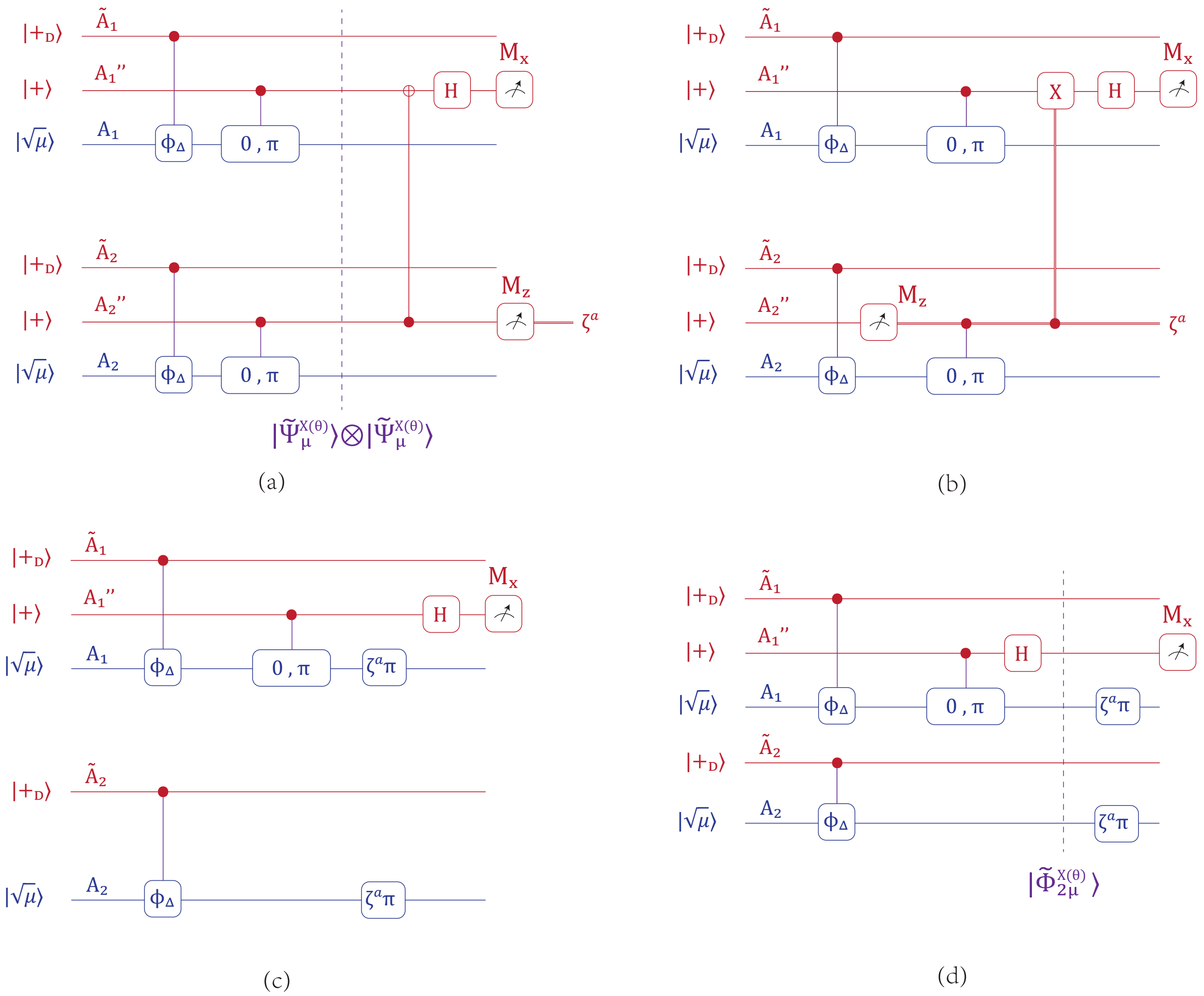

Supplementary Figure 11. Reduction of encoding in Supplementary Figure 9(b) to the $X(\theta)$-basis encoding state $|\tilde{\Phi}_{2\mu}^{X(\theta)}\rangle$ in Eq. (26) and Supplementary Figure 8(b). From (a) to (b), we advance the Z-basis measurement on A_2''. This entails introducing an extra random number ζ^a in the phase modulations of A_1 and A_2, as shown in (c). We remark that, a simultaneous phase modulation will not affect the security and performance of the $X(\theta)$-basis coherent-state MDI-QKD.

A_i''. Alice then performs global measurement $M(k,\theta)$ defined in Eq. (13) on qudits $\tilde{A}_i$ and $\tilde{A}_j$ to obtain the photon number k^a and the relative phase θ^a. Bob operates similarly. For all X pairs, Alice and Bob further announce θ^a and θ^b. If $\theta^a = \theta^b$, they keep the X-pair data; if $\theta^a - \theta^b = \pi$, they keep the X-pair data with Bob applying the Z gate on his left qubit. Moreover, if Charlie's announcement on locations i and j is (L,R) or (R,L), Bob applies Z gate on the qubit system B_i'' for the X-pairs. Alice and Bob then measure the ancillary qubits A'' and B'' of all the X-pairs and the ancillary qubits A' and B' of all the Z-pairs on the X- and Z-bases, respectively, to obtain the raw key bits κ^a and κ^b.

6. Parameter estimation: Alice and Bob estimate the fraction of clicked single-photon signals $q_{(1,1)}$ and the corresponding phase-error rate $e_{(1,1)}^{X(\theta)}$ in the Z-pairs, with Alice and Bob both emitting a single photon at locations i and j.

7. Key distillation: Alice and Bob reconcile the key string to κ^a and perform privacy amplification using universal-

2 hashing matrices. The sizes of hashing matrices are determined by the estimated single-photon yield $q_{(1,1)}$ and the $X(\theta)$-basis error rate $e^{X(\theta)}_{(1,1)}$ according to Eq. (38).

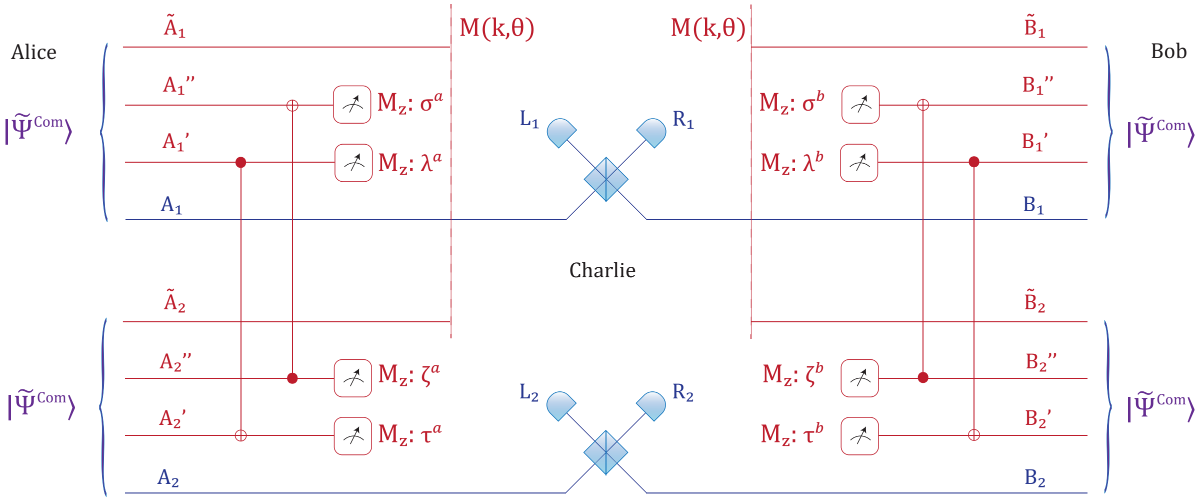

Supplementary Figure 12. Diagram of the entanglement-based MP scheme with a fixed pairing location. Here we take the pair on location 1 and 2 for example. Alice and Bob both generate a composite state $|\tilde{\Psi}^{Com}\rangle$ on an optical mode, two ancillary qubits and a ancillary qudit in each round and send the optical modes to Charlie. After Charlie's detection announcement, they perform control operations on the ancillary qubits at the predetermined paired locations and perform measurements on them. They then perform Z-basis measurements on all the ancillary qubits $A_1'(B_1')$, $A_1''(B_1'')$, $A_2'(B_2')$ and $A_2''(B_2'')$ to obtain the measurement results $\sigma^a(\sigma^b)$, $\lambda^a(\lambda^b)$, $\zeta^a(\zeta^b)$, $\tau^a(\tau^b)$. The usage of these results is listed in Supplementary Figure 18(d).

We introduce encoding redundancies in the coherent-state MDI-QKD scheme, so that the proposed MP scheme owns i.i.d. state preparation process in each round. Due to this i.i.d. encoding property, we can see that the MP scheme can be reduced to the coherent-state scheme based on Lemma 3 *for any given pairing setting* $\vec{\chi}$. We now clarify this lemma.

Lemma 4 (Security of the MP scheme under any fixed pairing setting)**.** *In the MP scheme, for any given pairing setting $\vec{\chi}$, suppose that Alice and Bob perform the mode-pairing operations on the ancillary qubits $\{A_i', A_i'', B_i', B_i''\}_i$ in Lemma 3 and the pairwise measurement $M(k,\theta)$ defined in Eq. (13) on the ancillary qudits $\{\tilde{A}_i, \tilde{B}_i\}_i$. When the discrete phase number $D \to \infty$, we have,*

1. *(Photon-number Poisson statistics) For the (i,j)-location pairs with overall intensities $\mu^a := \mu_i^a + \mu_j^a, \mu^b := \mu_i^b + \mu_j^b$, the photon-number-measurement results (k^a, k^b) of Alice and Bob's pairwise measurement $M(k,\theta)$ follow Poisson distributions,*

$$\Pr(k^a, k^b) = e^{-(\mu^a+\mu^b)} \frac{(\mu^a)^{(k^a)}(\mu^b)^{(k^b)}}{k^a!k^b!}. \tag{45}$$

2. *(Independence of the photon-number states to the intensity) The resultant state after the joint measurement $M(k,\theta)$ is independent of the intensity values μ^a and μ^b.*

3. *(Basis-independence of the single-photon pairs) For the location pairs with the overall photon-number measurement result $k^a = k^b = 1$, the $X(\theta)$-basis error rate can be used as a fair estimation of the Z-basis phase-error rate.*

As a result, with any fixed pairing $\vec{\chi}$, the $\vec{\chi}$-fixed-pairing scheme is able to generate secure key strings with the tagging key-rate formula, Eq. (38).

Proof. The process of a $\vec{\chi}$-fixed-pairing scheme can be sketched in Supplementary Figure 13(a).

In the fixed-pairing scheme, Alice and Bob start with $2n$ rounds of the state $|\tilde{\Psi}^{Com}\rangle$ defined in Eq. (40). Based on pairing strategy $\vec{\chi}$, they first perform the mode-pairing operation described in Lemma 3 to (1) decide the X/Z-basis of

the pairs of rounds; (2) reduce the encoding state to the encoding state of coherent-state-based MDI-QKD. Here, we denote the randomly determined X/Z-basis information of Alice's and Bob's on the location pair (i,j) are stored as two random numbers $\tau^a_{i,j}$ and $\tau^b_{i,j}$, respectively. From Lemma 3, we can see that under any pairing setting $\vec{\chi}$, with the given basis-assignment and post-selection condition, Alice and Bob can generate the encoding states $|\tilde{\Phi}^Z_\mu\rangle$ for $\tau^a_{i,j}=1$ or $|\tilde{\Phi}^{X(\theta)}_{2\mu}\rangle$ for $\tau^a_{i,j}=0$ in the coherent-state MDI-QKD scheme. The value of $\tau^a_{i,j}$ or $\tau^b_{i,j}$ follows a uniform distribution of 0 and 1.

After that, they perform a pairwise measurement $M(k,\theta)$ defined in Eq. (13) on the post-selected state. From Lemma 2, we see that the results (k^a,k^b) of $M(k,\theta)$ defined in Eq. (13) obey the Poisson distribution; after the pairwise measurement $M(k,\theta)$, the post-selected photon-number state is independent of the intensity setting μ^a and μ^b; the post-selected encoding states with $k^a=k^b=1$ will become to the one in the single-photon MDI-QKD scheme, $|\Phi^{Sin}_\theta\rangle$ in Eq. (20). If Alice and Bob post-select the corresponding encoding states, the security of this scheme will be equivalent to the single-photon MDI-QKD scheme, where the encoding state is basis-independent and the security proof can be easily done based on the complementarity.

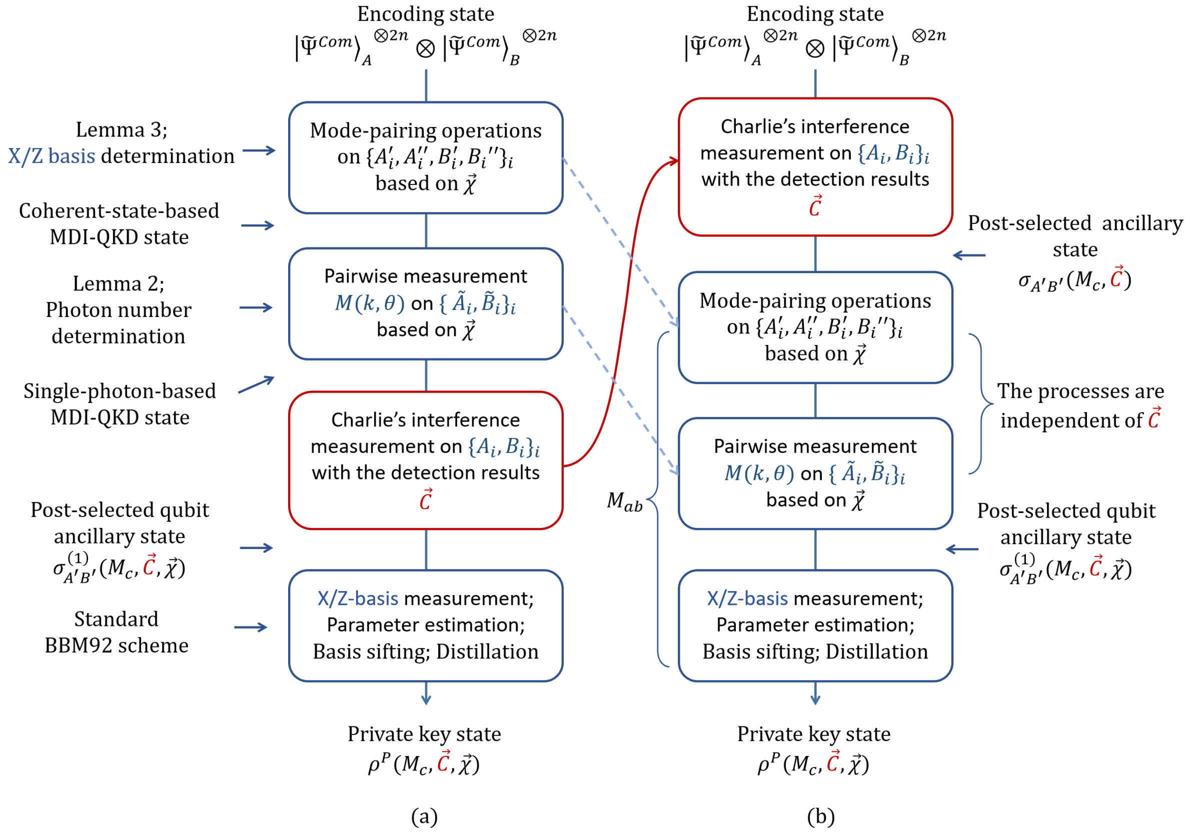

Supplementary Figure 13. Sketch of the proof of the security of $\vec{\chi}$-fixed-pairing scheme. (a) The proof logic in Lemma 4 based on encoding state reduction. (b) Since Charlie's measurement commutes with other operations on the ancillary systems, we can move it to the beginning.

If we set the photon numbers k^a and k^b to be the tag on the MP scheme, the key rate of the MP scheme also follows the traditional tagging key-rate formula in Eq. (38). □

In Supplementary Figure 13(a), after Alice and Bob post-select the rounds with encoding state $|\Phi^{Sin}_\theta\rangle$, they will emit the optical modes $\{A_i,B_i\}$ to Charlie, who will perform some unknown measurement M_c and announce the detection result $\vec{C}$.

In the following security proof, we will consider the security of the post-selected ancillary state conditional on a specific Charlie's announcement $\vec{C}$. In the fixed-pairing scheme in Supplementary Figure 13(a), we denote the normalized post-selected ancillary state at Alice and Bob's hand conditional on Charlie's specific measurement M_c and announcement $\vec{C}$ as $\sigma^{(1)}_{A'B'}(M_c,\vec{C},\vec{\chi})$. Here, the superscript (1) denotes that the state is generated from the

single-photon source state with $k^a = k^b = 1$. Alice and Bob then perform parameter estimation and distil private key state $\rho^P(M_c, \vec{C}, \vec{\chi})$ from $\sigma^{(1)}_{A'B'}(M_c, \vec{C}, \vec{\chi})$. The X/Z-measurement bases are determined by the random numbers $\tau^a_{i,j}$ and $\tau^b_{i,j}$ generated during the mode-pairing operations. Based on Lemma 4, we will have the following proposition.

Proposition 1 (Strong basis-independence of the fixed-pairing scheme)**.** *In a $\vec{\chi}$-fixed-pairing scheme illustrated in Supplementary Figure 13(a), for any Charlie's untrusted quantum measurement M_c and its result $\vec{C}$, Alice and Bob perform mode-pairing operation and photon number measurement based on the pairing setting $\vec{\chi}$, then perform loss sifting based on $\vec{C}$, and keep a normalized conditional state $\sigma^{(1)}_{A'B'}(M_c, \vec{C}, \vec{\chi})$ with $k^a = k^b = 1$. Then, for any location pair (i, j), the X/Z-basis information, $\tau^a_{i,j}$ and $\tau^b_{i,j}$ of the state $\sigma^{(1)}_{A'B'}(M_c, \vec{C}, \vec{\chi})$ generated by the mode-pairing operation on different location pairs follows an i.i.d. uniform distribution, which is independent of M_c and $\vec{C}$.*

Proof. In Lemma 4 we prove that, when $k^a = k^b = 1$, the post-selected encoding state is single-photon state $|\Phi^{Sin}_\theta\rangle$ in Eq. (20). The rotation angle θ is determined by the pairwise measurement $M(k, \theta)$.

Without loss of generality, we focus on the basis choice $\tau^a_{i,j}$ of Alice on a location pair (i, j). We remark that, the single-photon encoding states $|\Phi^{Sin}_\theta\rangle$ are the same for both Z- and $X(\theta)$-bases, corresponding to the case of $\tau^a_{i,j} = 1$ and $\tau^a_{i,j} = 0$, respectively. Moreover, the subsystem state of $|\Phi^{Sin}_\theta\rangle$ on the emitted optical modes A_i and A_j is independent of the alignment angle θ. As a result, Charlie's measurement and post-selection based on the optical mode systems cannot bias the basis choices $\tau^a_{i,j}$ and $\tau^b_{i,j}$. □

As a last step of the security proof of the fixed-pairing scheme, we notice that Charlie's measurement M_c commutes with the mode-pairing operation and the pairwise measurement $M(k, \theta)$ since they act on different quantum systems. Therefore, we can move Charlie's measurement before the operations acted on the ancillary systems, as shown in Supplementary Figure 13(b). The security of the scheme in Supplementary Figure 13(a) is equivalent to the on in Supplementary Figure 13(b). Based on Lemma 3 and Proposition 1, we can prove the security claim of the fixed-pairing scheme below.

Proposition 2 (Security of the fixed-pairing scheme conditional on Charlie's measurement results)**.** *In a fixed-pairing scheme, $\forall\vec{\chi}$ in Definition 1, for any Charlie's untrusted quantum measurement M_c and any measurement announcement $\vec{C}$ in Definition 2, the post-selected normalized ancillary state $\sigma_{A'B'}(M_c, \vec{C})$ in Supplementary Figure13(b) conditional on $\vec{C}$ at Alice and Bob's hand can be used to distil a private key state $\rho^P(M_c, \vec{C}, \vec{\chi})$ with the tagging key-rate formula, Eq. (38).*

Proof. The proof can be easily seen from the equivalence of the fixed-pairing scheme in Supplementary Figure 13(a) and Supplementary Figure 13(b), Lemma 3 and Proposition 1. □

D. Free-pairing MP scheme

The freedom of choosing the pairing setting, $\vec{\chi}$, in the fixed-pairing MP scheme inspires us to consider a more flexible variant, where $\vec{\chi}$ is determined based on Charlie's announcements $\vec{C}$. We call this variant the free-pairing MP scheme, in Definition 5. Note that the pairing setting, $\vec{\chi} \in \mathcal{X}$ in Definition 1, is chosen for all the N emitted signals, even for the locations without successful detection. This is due to the usage of Lemma 4 in the latter discussion.

Definition 4 (Pairing Strategy T)**.** *For N location indexes $i = 1, 2, ..., N$ where N is an even number, a pairing strategy, $T : \mathcal{C} \to \mathcal{X}$, is defined to be a map from Charlie's announcement $\vec{C} \in \mathcal{C}$ in Definition 2 to the pairing setting $\vec{\chi} \in \mathcal{X}$ in Definition 1. The possible pairing strategies form a finite set $\mathcal{T}$.*

Definition 5 (T-Free-Pairing MP)**.** *In a MP MDI-QKD scheme, if pairing setting $\vec{\chi}$ depends on Charlie's announcement, then we call it the free-pairing MP scheme. A free-pairing MP scheme with pairing strategy $T \in \mathcal{T}$ is called the T-free-pairing MP scheme.*

We remark that, the pairing setting $\vec{\chi}$ in Definition 1 and the pairing strategy T in Definition 4 are defined for *all the locations*, even for the ones with unsuccessful detections. In a pairing strategy T, we need to pair all the locations based on Charlie's announcement $\vec{C}$ and do not perform any post-selection. The pairing definition in this way is helpful to build up a connection between the fixed- and free-pairing schemes, so that the loss sifting in the free-pairing schemes will be handled following the same way as the fixed-pairing schemes. For example, suppose there are 4 rounds in the mode-pairing scheme and Charlie announces successful detections on locations 1 and 4, then based on a simple pairing strategy, Alice and Bob pair the locations as follows: $(1, 4)$, $(2, 3)$. The "lost" pair $(2, 3)$ will be used for the parameter estimation.

We also note that, the $\vec{\chi}$-fixed-pairing scheme can be regarded as a special case of the T-free-pairing scheme, where the pairing strategy is a constant function, $T(\vec{C}) \equiv \vec{\chi}$, $\forall \vec{C} \in \mathcal{C}$. Following the fixed-pairing MP scheme in Box 3, we present the procedures of the free-pairing MP scheme with a pairing strategy T in Box 4.

Box 4: Free-pairing MP scheme

1. State preparation: Same as the fixed-pairing MP scheme in Box 3.
2. Measurement: Same as the fixed-pairing MP scheme in Box 3.

   Alice and Bob perform the above steps over many rounds. After that, they perform the following data post-processing steps.
3. Mode Pairing: Charlie announces $\vec{C} \in \mathcal{C}$ of Definition 2, based on which Alice and Bob determine a pairing setting $\vec{\chi} = T(\vec{C}) \in \mathcal{X}$ of Definition 1 using a predetermined pairing strategy $T \in \mathcal{T}$ of Definition 4.
4. Basis Sifting: Same as the fixed-pairing MP scheme in Box 3.
5. Key mapping: Same as the fixed-pairing MP scheme in Box 3.
6. Parameter estimation: Same as the fixed-pairing MP scheme in Box 3.
7. Key distillation: Same as the fixed-pairing MP scheme in Box 3.

In the free-pairing scheme, Charlie announces $\vec{C}$ and then Alice and Bob determine $\vec{\chi}$ based on $\vec{C}$. In a way, Charlie can manipulate the choice of paring settings.

Before we examine the free-pairing scheme, we first briefly review the process of fixed-pairing scheme and revisit what we proved in Lemma 4 and Proposition 2. A simple diagram of $\vec{\chi}$-fixed-pairing scheme is shown in Supplementary Figure 14(a), as a special case of Supplementary Figure 3. Alice and Bob first generate $N = 2n$ rounds of the encoding state $\rho_{ABA'B'}$ as the pure state $|\tilde{\Psi}^{Com}\rangle_{Alice} \otimes |\tilde{\Psi}^{Com}\rangle_{Bob}$ defined in Eq. (40). They send all the optical signals of systems A and B to Charlie, who performs unknown measurement M_c on them and announces the result $\vec{C}$ defined in Definition 2. Note that M_c refers to all Charlie's operations, including measurement, result processing, and announcement strategy.

Based on M_c and $\vec{C}$, Alice and Bob's post-selected ancillary state $\sigma(M_c, \vec{C})$ at hand is supposed to be entangled if Charlie honestly follows the designed measurement. Afterwards, in the $\vec{\chi}$-fixed-pairing scheme, Alice and Bob perform the key-generation operation $M_{ab}(\vec{C}, \vec{\chi})$, including basis-sifting, key mapping, parameter estimation, and key distillation (i.e., Steps 4-7 in Box 3) based on a pre-determined pairing setting $\vec{\chi}$ in Definition 1 and Charlie's announcement $\vec{C}$. Following Lemma 4 and Proposition 2, we have proven that, for *all possible Charlie's measurement operation* M_c *and announcement result* $\vec{C}$, the final state $\rho^P(M_c, \vec{C}, \vec{\chi})$ will be private for every $\vec{\chi} \in \mathcal{X}$.

In what follows, we prove the security of the free-pairing scheme by its *conditional equivalence* to the fixed-pairing scheme and Proposition 2. We now clarify the conditional equivalence argument.

Proposition 3 (Conditional equivalence of two MP schemes)**.** *For two different MP schemes with pairing strategies* T_1 *and* T_2*, respectively, if measurement* M_c *and announcement* $\vec{C}$ *are the same, then post-selected ancillary states* $\sigma_{A'B'}(M_c, \vec{C})$ *for these two schemes will be the same. Moreover, if we have* $T_1(\vec{C}) = T_2(\vec{C})$*, that is, pairing settings* $\vec{\chi}_1$ *and* $\vec{\chi}_2$ *are the same, then the resulting states after key distillation will be the same.*

Proof. To begin with, we list the variable dependence in a T-free-pairing scheme in Supplementary Figure 15. We can see how Charlie's measurement affect the pairing setting $\vec{\chi}$ and the private key state $\rho^P(M_c, \vec{C}, \vec{\chi})$.

We notice that all the MP scheme share the same encoding state $\rho_{ABA'B'}$, that is $|\tilde{\Psi}^{Com}\rangle$ defined in Eq. (40). Then from the variable dependence in Supplementary Figure 15, one can clearly obtain this proposition. □

We remark that, this conditional equivalence argument is weaker than the one we define in Lemma 1. In Proposition 3, we only compare the post-selected ancillary state under a specific Charlie's measurement M_c and announcement $\vec{C}$. However, combined with Proposition 2, this is sufficient for a complete security proof of a T-free-pairing scheme.

Theorem 1 (Security of the free-pairing MP scheme)**.** *For any pairing strategy* $T \in \mathcal{T}$ *in Definition 4, under all Charlie's measurement operations* M_c *and announced results* $\vec{C}$*, in the* T*-free-pairing scheme, Alice and Bob is able to generate private key state* $\rho^P(M_c, \vec{C}, \vec{\chi})$ *with the tagging key-rate formula Eq. (38).*

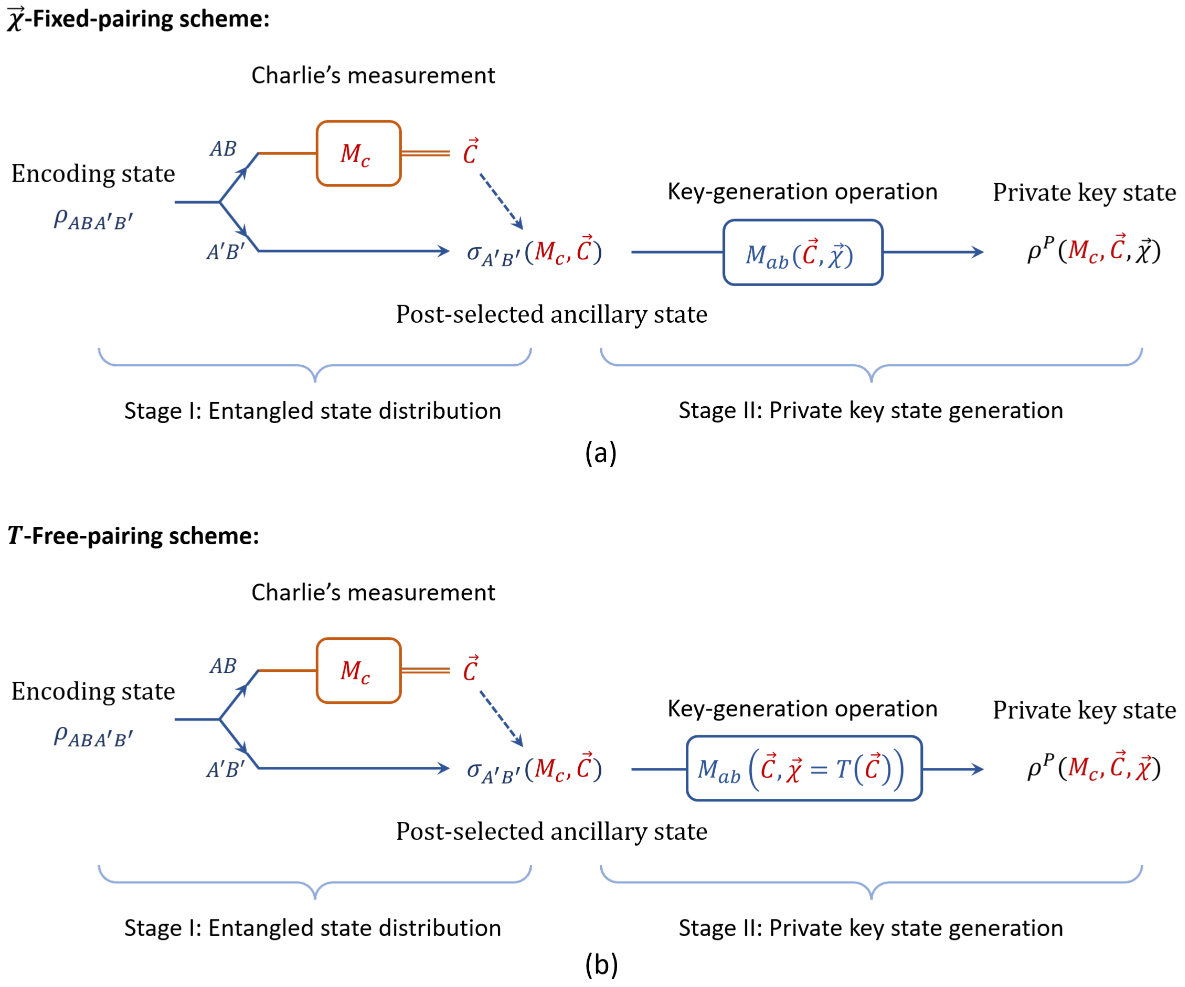

Supplementary Figure 14. Flowcharts of (a) $\vec{\chi}$-fixed-pairing scheme and (b) T-free-pairing scheme. Here, the key-generation operation M_{ab} corresponds to Steps 4-7 in Box 3 and 4. The major difference of the fixed-pairing and free-pairing scheme lies in how the key-generation operation M_{ab} depends on the pairing setting $\vec{\chi}$ and how the pairing setting is determined by Charlie's announcement $\vec{C}$.

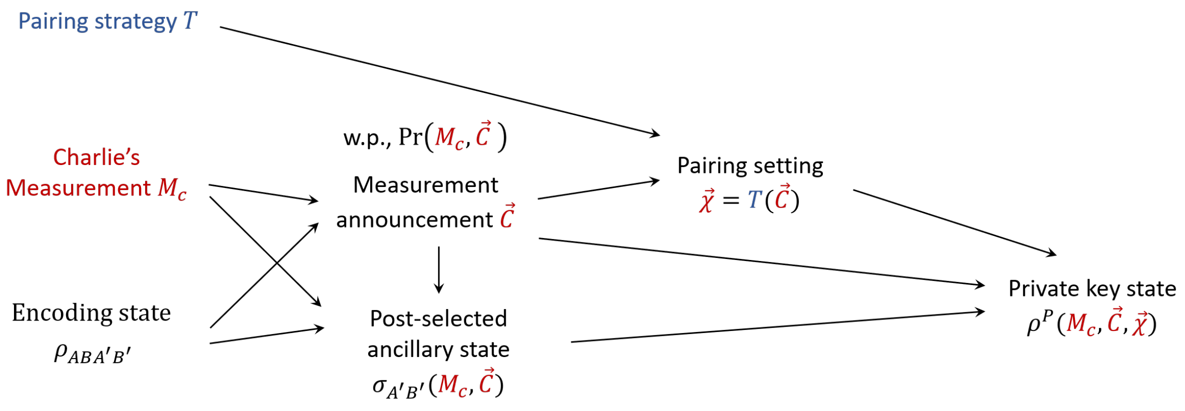

Supplementary Figure 15. Variable dependence in a T-free-pairing scheme. The variables linked by arrows form a Markov chain.

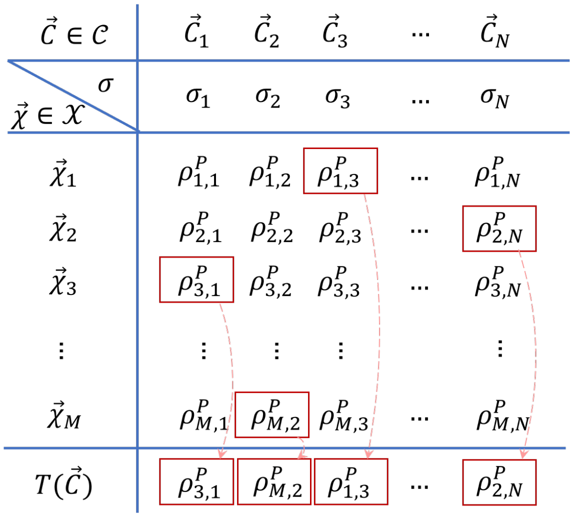

Supplementary Figure 16. Security of the free-pairing MP scheme. Given Charlie's measurement M_c, we list all possible ancillary states $\sigma_{\vec{C}} := \sigma(\vec{C})$ and the private key states $\rho^P_{\vec{\chi},\vec{C}} := \rho^P(\vec{C},\vec{\chi})$ with different announcements $\vec{C}$ and pairing settings $\vec{\chi}$. Adopting a free pairing strategy $T(\vec{C})$ in the key-generation procedure is essentially to pick out a private key state ρ^P for each announcement $\vec{C}$ according to $\vec{\chi} = T(\vec{C})$. For the example shown in the figure, connected by dashed red arrow lines, $T(\vec{C}_1) = \vec{\chi}_3$, $T(\vec{C}_2) = \vec{\chi}_M$, $T(\vec{C}_3) = \vec{\chi}_1$, and $T(\vec{C}_N) = \vec{\chi}_2$.

Proof. We are going to prove the following three statements,

1. In any fixed-pairing scheme where $T(\vec{C}) \equiv \vec{\chi} \in \mathcal{X}$, for any possible Charlie's measurement operation M_c and announcement result $\vec{C}$, after the key-generation operation M_{ab} (i.e., Steps 4-7 in Box 3) , the final state ρ^P is private with the tagging key-rate formula Eq. (38).

2. All possible Charlie's measurements M_c in a T-free-pairing scheme can be realized in every fixed-pairing scheme[1].

3. Given Charlie's measurement M_c, the final state generated by a T-free-pairing scheme is equal to the private state $\rho^P(M_c, \vec{C}, \vec{\chi})$ generated by one of the $\vec{\chi}$-fixed-pairing schemes.

The first statement is a direct result of Lemma 4 and Proposition 2.

To prove the second statement, we notice that the encoding state prepared in both free-pairing and fixed-pairing schemes are the same. Then, Charlie receives exactly the same quantum states in the two schemes. The only difference is that Charlie receives different classical information of the pairing strategies $T \in \mathcal{T}$. Recall that the fixed pairing $\vec{\chi}$ is a special pairing strategy in $\mathcal{T}$. The second statement becomes the following question: for any Charlie's measurements M_c in a T-free-pairing scheme, whether Charlie can perform the same M_c in a fixed-pairing scheme. Since the pairing strategy T is independent of the quantum states sent to Charlie, the answer is yes and hence the second statement holds.

Now, we prove the third statement. For a given Charlie's measurement M_c, we can list the resultant ancillary states $\sigma(\vec{C})$ at Alice and Bob's hand corresponding to different measurement results $\vec{C}$, shown in Supplementary Figure 16. Furthermore, we list all the possible resultant states ρ^P after the key-generation operation M_{ab} correspond to different fixed pairing settings $\vec{\chi} \in \mathcal{X}$ in the fixed-pairing MP schemes, shown in Supplementary Figure 16.

In the T-free-pairing scheme in Definition 5, Alice and Bob choose the pairing setting $\vec{\chi} = T(\vec{C})$ during the key generation process M_{ab}. For a pairing strategy $T \in \mathcal{T}$ in Definition 4, we have $T(\vec{C}) \in \mathcal{X}$. In this case, in a T-free-pairing scheme, the resultant state after the key-generation operation will be the same as a private key state from one of the fixed-pairing schemes based on Proposition 3.

Combining the three statements, we conclude that: under any possible Charlie's measurement operation M_c and announcement $\vec{C}$, a T-free-pairing scheme results in a private key state with the key length determined by the tagging key-rate formula Eq. (38). □

---

[1] That is, the set of possible M_c in the free-pairing scheme is contained in that of fixed-pairing scheme. Strictly speaking, the sets of possible M_c in the two cases are the same, since it only depends on the state Alice and Bob prepare, which is independent of T-free-pairing or χ-fixed-pairing.

Having said that Alice and Bob can choose the pairing strategy T freely even based on Charlie's announcement $\vec{C}$, the pairing setting $\vec{\chi}$ cannot depend on the measurement results of Alice's and Bob's ancillary states. This is due to the fact that in our security proof, the pairing setting $\vec{\chi}$ is determined before any operation on the ancillary states.

E. Security analysis with decoy-state estimation

In the entanglement-based security analysis for the fixed-pairing and free-pairing schemes in Supplementary Note 2 C and Supplementary Note 2 D, we assume that Alice and Bob are able to perform pairwise measurement $M(k, \theta)$ to learn photon number k^a and k^b on each paired signals. In the prepare-and-measure MP scheme that we shall consider below, Alice and Bob do not actually perform such a photon-number measurement. To solve this problem, we introduce the decoy-state method. In this section, we discuss how the proof methods in Supplementary Note 2 C and Supplementary Note 2 D can be naturally extend to the decoy-state analysis.

We first consider the fixed-pairing scheme in Box 3, as illustrated in Supplementary Figure 13. In the decoy-state version of the scheme, instead of performing the pairwise measurement $M(k, \theta)$, Alice and Bob consider the following problem during the parameter estimation.

Problem 1 (Decoy-state estimation)**.** *In the $\vec{\chi}$-fixed-pairing scheme, after Charlie's measurement M_c and announcement $\vec{C}$, Alice and Bob perform mode-pairing operations to obtain the basis and raw key information for each pair.*

1. *Among all the Z-pairs with successful detection results, if Alice and Bob were able to perform pairwise photon-number measurement $M(k, \theta)$, what is the ratio of them with $k^a = k^b = 1$, denoted $q_{(1,1)}$?*

2. *Among all the X-pairing with successful detection and same alignment angle $\theta^a = \theta^b$, if Alice and Bob were able to perform the pairwise photon-number measurement $M(k, \theta)$, what is the bit error rate $e^X_{(1,1)}$ for the raw key information with $k^a = k^b = 1$?*

The answer to this problem, $q_{(1,1)}$ and $e^X_{(1,1)}$, is sufficient for us to perform privacy amplification based on the tagging key rate formula in Eq. (38). To solve Problem 1 in the fixed-pairing scheme, we have the following proposition, as a corollary to Lemma 4.

Proposition 4 (Independence of photon number yields with respect to intensities)**.** *In the fixed-pairing MP scheme in Box 3, for any given pairing setting $\vec{\chi}$, suppose Alice and Bob perform pairwise photon-number measurement $M(k, \theta)$ defined in Eq. (13) on ancillary qudits $\{\tilde{A}_i, \tilde{B}_i\}_i$. Charlie receives the optical modes $\{A_i, B_i\}_i$, performs measurement M_c, and announces the detection results $\vec{C}$. Suppose the discrete phase number is $D \to \infty$. Alice and Bob then classify all the location pairs based on measured results (k^a, k^b). Denote the overall intensity on the (i, j)-location pairs as $\mu^a := \mu^a_i + \mu^a_j, \mu^b := \mu^b_i + \mu^b_j$. For the location pairs with same (k^a, k^b), the probability of the pairs to be successfully detected is independent of the intensities μ^a and μ^b of the emitted signals.*

Proof. According to Lemma 4, if Alice and Bob perform the pairwise measurement $M(k, \theta)$ defined in Eq. (13) on ancillary qudits $\{\tilde{A}_i, \tilde{B}_i\}_i$ before they send optical modes $\{A_i, B_i\}_i$ to Charlie, then the conditional state on the optical modes will be independent of intensity μ^a and μ^b. As a result, Charlie's measurement M_c and announcement $\vec{C}$ cannot depend on intensities μ^a and μ^b. □

Proposition 4 essentially shows a source property of the MP encoding state, irrelevant of Charlie's measurement M_c or announcement $\vec{C}$. Since pairwise photon-number measurement $M(k, \theta)$ and Charlie's measurement M_c act on different systems, they commute with each other. As a result, if measurement $M(k, \theta)$ is performed after Charlie's measurement M_c and announcement $\vec{C}$, Proposition 2 still holds.

In the decoy-state version of entanglement-based fixed-pairing scheme, Alice and Bob introduce extra intensities during the state preparation procedure. They can do it by replacing intensity-encoding ancillary qubits A' and B' to qudits with high dimensions. The mode-pairing operation can then be accordingly modified, based on the scheme introduced in Methods's subsection "Mode-pairing scheme with decoy state". One can follow a similar encoding-state-reduction procedure in Lemma 3 to reduce the encoding states to the ones of coherent-state-based MDI-QKD schemes. Based on Lemma 4 and Proposition 4, Alice and Bob can then characterize $q_{(1,1)}$ and $e^X_{(1,1)}$ by counting the statistics of detections with different overall intensities (μ^a, μ^b). The detailed decoy-state estimation procedure is introduced in Supplementary Note 3.

We compare the original fixed-pairing scheme with pairwise measurement $M(k, \theta)$ and the decoy-state version of fixed-pairing scheme in Supplementary Figure 17. Denote Alice and Bob's new key generation operation with decoy-state estimation as $M'_{ab}(\vec{C}, \vec{\chi})$.

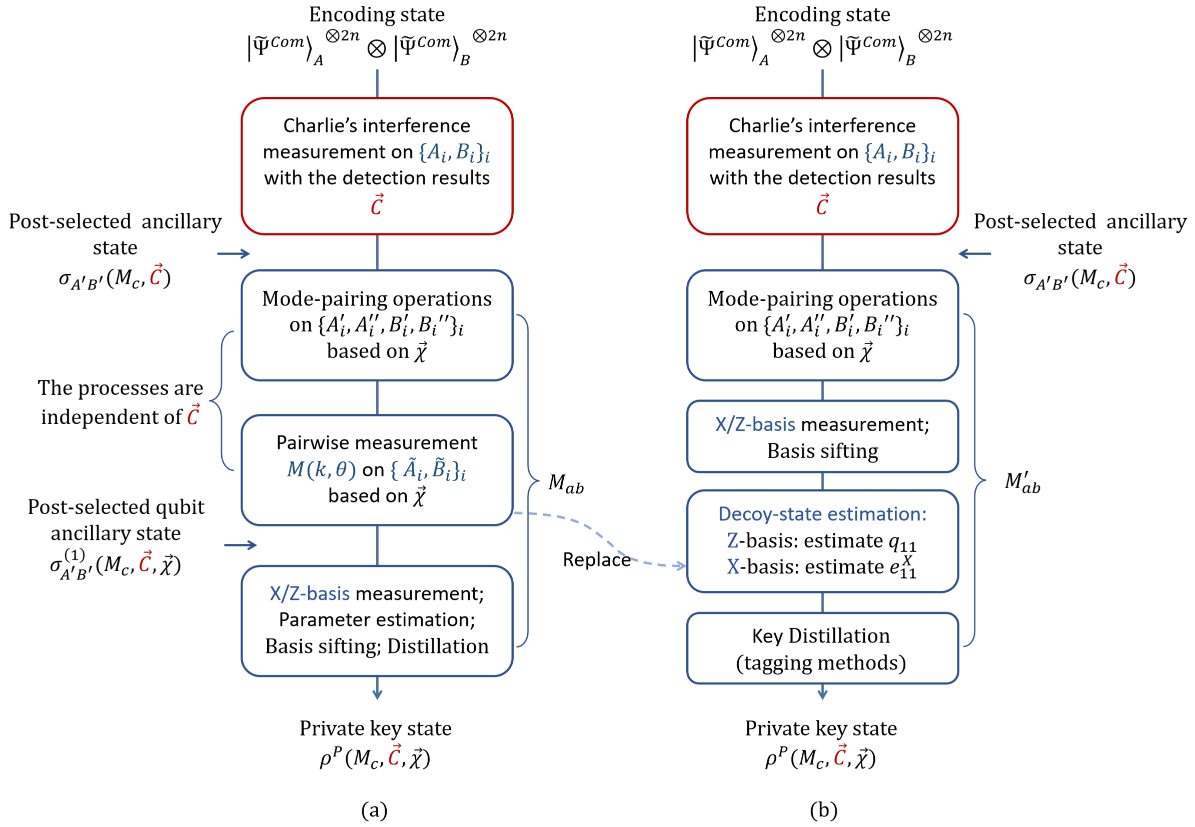

Supplementary Figure 17. (a) Original fixed-pairing scheme with pairwise photon-number measurement $M(k,\theta)$. (b) To remove the pairwise measurement, Alice and Bob can introduce decoy-state estimation to solve Problem 1. The key generation operation is now denoted as M'_{ab}.

Now, let us discuss how to show the feasibility of the decoy-state estimation in the free-pairing schemes by the conditional equivalence argument in Proposition 3.

To introduce the decoy-state estimation in the free-pairing scheme with pairing strategy T, we first suppose that Alice and Bob choose to use the key generation operation $M'_{ab}(\vec{C}, \vec{\chi})$ with $\vec{\chi} = T(\vec{C})$ with decoy-state estimation same as the $\vec{\chi}$-fixed-pairing scheme.

The conditional equivalence arguments in Proposition 3 still holds if we consider all the fixed-pairing and free-pairing schemes, after including the decoy-state estimation into the key generation operation, $M'_{ab}(\vec{C}, \vec{\chi})$. Therefore, we can follow exactly the same argument to prove the security of the free-pairing scheme with decoy-state estimation as we have done in Theorem 1.

F. Prepare-and-measure MP scheme

As the final step, we reduce the entanglement-based free-pairing MP scheme in Box 4 to the prepare-and-measure one presented in the main text. In the following discussions, we focus on the reduction on Alice's side. The reduction of Bob's side follows the same manner. Also, for the simplicity of discussion, we take a paired location 1 and 2 as an example.

First, we remove all the pairwise joint measurements $M(k,\theta)$ defined in Eq. (13) used to read out the global photon number and the relative phase on each pair of optical modes. Instead, we assume Alice and Bob measure the (discrete) phase of each pulse directly. In this way, the relative phase can be obtained by classical post-processing. Then, the global photon number is lost due to the uncertain relationship between phase and photon number measurements. In practice, Alice and Bob can use the decoy-state method to estimate the fraction of the single-photon component. Furthermore, we assume the random phase is measured at the beginning, which is then equivalent to a random phase encoding on the emitted optical pulses. In this way, we remove the two ancillary qudits $\tilde{A}_1$ and $\tilde{A}_2$ in the encoding process.

After removing the phase encoding ancillary systems, we are now going to remove the four ancillary qubits A_1', A_1'', A_2' and A_2''. To do this, we move all the measurements on the ancillary qubits mentioned in Lemma 3 forward. In Supplementary Figure 18(a), we list the complete basis and key-assignment procedure of the entanglement-based MP scheme. The final measurement results $\sigma^a, \lambda^a, \zeta^a$ and τ^a are used in different ways, as shown in Supplementary Figure 18(d). Note that the Z-basis measurements commute with all $CNOT$ gates. Therefore, moving all measurements before the control gates between locations 1 and 2, one can equivalently obtain the measurement results $\sigma^a, \lambda^a, \zeta^a$, and τ^a by classical post-processing, as shown in Supplementary Figure 18(b).

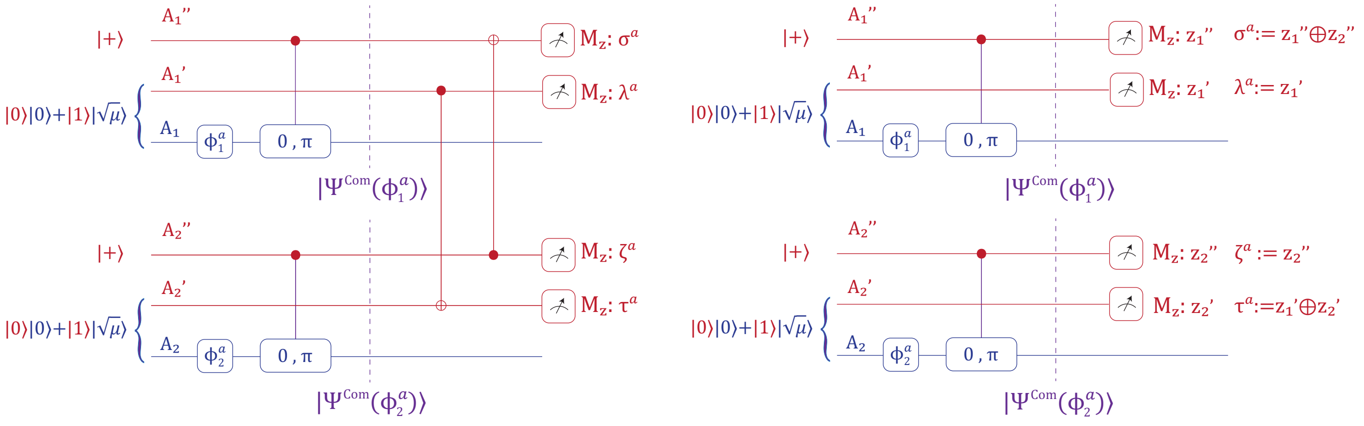

(a) (b)

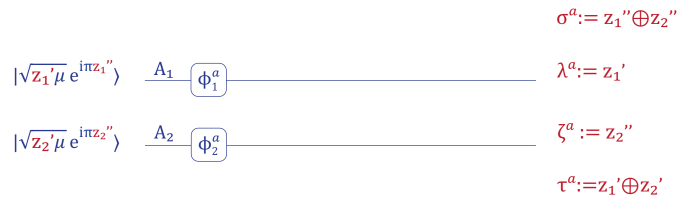

(c)

Variables	Z-basis	X(θ)-basis
$\sigma^a := z_1'' \oplus z_2''$	No use	Key generation
$\lambda^a := z_1'$	Key generation	Post-selection
$\zeta^a := z_2''$	No use	
$\tau^a := z_1' \oplus z_2'$	Basis assignment	

(d)

Supplementary Figure 18. (a) All of the operations that Alice performs on the ancillary qubits A_1'', A_1', A_2'' and A_2' to assign bases and generate raw key bits when the locations 1 and 2 are paired. τ^a is used for the basis assignment, λ^a is used for key generation in the Z-basis and post-selection in the $X(\theta)$-basis and σ^a is used for key generation in the $X(\theta)$-basis. (b) An equivalent process whereby Alice moves all the measurement before the global control gates between locations 1 and 2. The measurement results z_1'' and z_1' on A_1'' and A_1' reveal the encoded phase and the intensity on A_1, respectively. (c) Alice further measures all ancillaries in the beginning. This is equivalent to preparing the coherent states on A_1 and A_2 with modulated intensities and phases. (d) The values and usage of the variables $\sigma^a, \lambda^a, \zeta^a$ and τ^a.

In Supplementary Figure 18(b), the measurement results z_1' and z_1'' on A_1' and A_1'' reveal the encoded intensity and phase on A_1, respectively. Alice further moves the measurements on the ancillary qubits to the beginning. It is then equivalent to preparing the coherent states $|\sqrt{z_1'\mu}e^{i\pi z_1''}\rangle, |\sqrt{z_2'\mu}e^{i\pi z_2''}\rangle$ on A_1 and A_2 with modulated intensities and phases. Thus, we reduce the entanglement-based scheme to a prepare-and-measure version.

Box 5: Prepare-and-measure MP scheme

1. State preparation: In the i-th round, Alice generates two random bits z_i' and z_i'' and a random phase ϕ_i^a uniformly and discretely chosen from $\{\frac{2\pi}{D}j\}_{j\in[D]}$. She generates a coherent state $|\sqrt{z_i'\mu}e^{i(\pi z_i''+\phi_i^a)}\rangle$. Similarly, Bob generates two random bits y_i' and y_i'', ϕ_i^b, and a coherent state $|\sqrt{y_i'\mu}e^{i(\pi y_i''+\phi_i^b)}\rangle$.

2. Measurement: Alice and Bob send the coherent states to Charlie, who is supposed to perform a single-photon interference measurement. Charlie announces the clicks of the L and/or R detectors.

   Alice and Bob perform the above steps over many rounds. Afterwards, they perform the following data

post-processing steps.

3. Mode pairing: Alice and Bob set a strategy to pair the clicked locations, i.e., to group each two detected rounds together based on Charlie's announcement.

4. Basis sifting: For each pair on locations i and j, Alice sets $\tau^a := z_i' \oplus z_j'$, $\lambda^a := z_i'$. Alice sets the basis of the pair to Z if $\tau^a = 1$, X if $\tau^a = 0$ and $\lambda^a = 1$, and '0' if $\tau^a = \lambda^a = 0$. Bob assigns the basis in the same way. Alice and Bob announce the bases. If the announced bases are X, X or Z, Z, they maintain the signals.

5. Key mapping: For each Z-pair on locations i and j, Alice records λ^a as the key bit. For each X-pair, Alice sets $\sigma^a := z_i'' \oplus z_j''$ as the key bit and records $\theta_{i,j}^a := \phi_i^a - \phi_j^a$ for later usage. For all X-pairs, Alice and Bob further announce θ^a and θ^b. If $\theta^a = \theta^b$, they keep the X-pair data; if $\theta^a - \theta^b = \pi$, they keep the X-pair data with Bob flipping the value of σ^b; otherwise, they discard the X-pair data. Moreover, if Charlie's announcement at locations i and j is (L, R) or (R, L), Bob flips the value of σ^b.

6. Parameter estimation: Alice and Bob use decoy-state methods to estimate the fraction of clicked signals $q_{(1,1)}$ and the corresponding phase-error rate $e_{(1,1)}^X$ in the Z-pairs, where Alice and Bob both emit a single photon at locations i and j. They also estimate the quantum bit-error rate $E_{(\mu,\mu)}^Z$ of the Z-pairs.

7. Key distillation: Alice and Bob reconcile the key string to κ^a and perform privacy amplification using universal-2 hashing matrices. The sizes of hashing matrices are determined by the estimated single-photon yield $q_{(1,1)}$ and the $X(\theta)$-basis error rate $e_{(1,1)}^{X(\theta)}$ according to Eq. (38).

To reduce the scheme to the MP scheme in the main text, we further simplify the announcement of the random phase in the X basis. Note that the encoding phase in the i-th round is $\phi_i^a + \pi z_i''$, with $\phi_i^a \in [0, 2\pi)$. The phase modulation of z_i'' is redundant in the experiment. We now try to absorb it into ϕ_i^a.

In the following discussion, without loss of generality, we first assume that θ^a and θ^b are equal to 0 or π. In this case, Alice and Bob's random phases on locations i and j are either 0 or π. We denote them as

$$\begin{aligned} \phi_i^a &= \pi\hat{\phi}_i^a, \quad \phi_j^a = \pi\hat{\phi}_j^a, \\ \phi_i^b &= \pi\hat{\phi}_i^b, \quad \phi_j^b = \pi\hat{\phi}_j^b, \end{aligned} \tag{46}$$

where $\hat{\phi}_i^a, \hat{\phi}_j^a, \hat{\phi}_i^b$ and $\hat{\phi}_j^b$ are four binary variables.

During the X-basis key mapping, Alice and Bob's raw key and random phase at locations i and j are

$$\begin{aligned} \sigma^a &:= z_i'' \oplus z_j'', \quad \theta^a := \pi(\hat{\phi}_i^a \oplus \hat{\phi}_j^a), \\ \sigma^b &:= y_i'' \oplus y_j'' \oplus \delta_\theta \oplus \delta_d, \quad \theta^b := \pi(\hat{\phi}_i^b \oplus \hat{\phi}_j^b). \end{aligned} \tag{47}$$

Here, the binary variable $\delta_\theta := (\hat{\phi}_i^a \oplus \hat{\phi}_j^a \oplus \hat{\phi}_i^b \oplus \hat{\phi}_j^b)$ indicates whether the announcements of θ^a and θ^b are the same or different. δ_θ describes the detection announcement in i and j: if the announcements are (L, L) and (R, R), then $\delta_d = 0$; if the announcements are (L, R) and (R, L), $\delta_d = 1$.

To simplify the key-mapping strategy, Alice and Bob absorb the random phase $z_i'', z_j''; y_i'', y_j''$ into $\phi_i^a, \phi_j^a; \phi_i^a, \phi_j^b$. They revise the raw key announcement as follows:

$$\begin{aligned} \bar{\sigma}^a &:= (\hat{\phi}_i^a \oplus z_i'') \oplus (\hat{\phi}_j^a \oplus z_j''), \\ \bar{\sigma}^b &:= (\hat{\phi}_i^b \oplus y_i'') \oplus (\hat{\phi}_j^b \oplus y_j''). \end{aligned} \tag{48}$$

Meanwhile, they set the announced basis angle to be $\bar{\theta}^a = \bar{\theta}^b = 0$. In this case, the announced information is less than the original scheme. Hence, the privacy of Alice's raw key $\bar{\sigma}^a$ will not worsen. The new raw key bit is related to the original raw key bit as

$$\begin{aligned} \bar{\sigma}^a &= \sigma^a \oplus (\hat{\phi}_i^a \oplus \hat{\phi}_j^a), \\ \bar{\sigma}^b &= \sigma^b \oplus (\hat{\phi}_i^b \oplus \hat{\phi}_j^b). \end{aligned} \tag{49}$$

Therefore, $\bar{\sigma}^a = \bar{\sigma}^b$ iff $\sigma^a = \sigma^b$. Up to a random phase difference, the new raw key bits are equivalent to the original ones.

In general, θ^a and θ^b may not be 0 or π. In this case, we revise the random phase announcement. Let

$$\theta_r^a := (\phi_i^a + \pi z_i'') - (\phi_j^a + \pi z_j'') = (\phi_i^a - \phi_j^a) + \pi(z_i'' - z_j'') = \theta^a + \pi(z_i'' \oplus z_j''). \tag{50}$$

Now, we further split θ_r^a into two parts,

$$\theta_r^a = \theta_0^a + \pi\kappa^a, \tag{51}$$

where $\theta_0^a := \theta_r^a \bmod \pi \in [0, \pi)$ denotes half of θ_r^a while $\kappa^a := (\theta_r^a/\pi) \bmod 2$. Using a similar method, one can show that $\kappa^a = \kappa^b$ iff $\sigma^a = \sigma^b$.

Therefore, in an equivalent but simplified $X(\theta)$-basis key mapping procedure, Alice (Bob) regards the phase $(\phi_i^a + z_i'')((\phi_i^b + y_i''))$ as a whole. They calculate the basis angle as Eq. (50) and (51). The corresponding revised scheme can now be reduced to the MP scheme in the main text.

Supplementary Note 3. DECOY-STATE ESTIMATION

Here, we apply decoy-state analysis [11] to the mode-pairing scheme, with the typical three-intensity settings — vacuum (zero intensity), weak (intensity ν) and signal (intensity μ) states. The discussion below can naturally be extended to the decoy-state method with an arbitrary number of intensities. In the mode-pairing scheme, Alice and Bob each independently generate coherent states with intensities μ_i^a and μ_i^b, respectively, for the i-th round. Suppose the state intensities are randomly chosen from the set $\{0, \nu, \mu\}$ with probabilities s_0, s_ν and s_μ, respectively, where, $s_0 + s_\nu + s_\mu = 1$.

We consider the pairing setting $\vec{\chi}$ in Definition 1, which defined on all the locations, including the locations with unsuccessful clicks. In the practical mode-pairing scheme, Alice and Bob decide the pairing setting based on Charlie's announcement $\vec{C}$, which is a $2N$-bit string. On each location, Charlie announces two-bit information (L_i, R_i), indicating the detection results of the two detectors L and R. The pairing strategy we mentioned in the main text is an algorithm to choose a specific pairing setting $\vec{\chi}$ based on Charlie's announcement $\vec{C}$. We simply pair all the unpaired locations (including the locations with unsuccessful detections and the ones discarded during the pairing process) in Algorithm 1 to construct a complete pairing setting $\vec{\chi}$.

In the following discussion, we consider the case that Alice and Bob choose a specific fixed pairing setting $\vec{\chi}$. Since the following decoy-state estimation methods holds for all pairing settings $\vec{\chi}$ from $\mathbf{X}$, it can also be applied to the pairing setting $\vec{\chi}$ chosen by the pairing strategy based on $\vec{C}$. A detailed discussion on the security of mode-pairing scheme with a pairing setting $\vec{\chi}$ chosen based on Charlie's announcement $\vec{C}$ can be found in Supplementary Note 2 D.

Let us first study the source property under a pairing setting $\vec{\chi}$. Alice and Bob pair two locations, i and j, based on $\vec{\chi}$ as one round of QKD. The intensity vector of the (i, j) pair is denoted as

$$\vec{\mu} = (\mu_i^a + \mu_j^a, \mu_i^b + \mu_j^b), \tag{52}$$

where $\mu_i^a, \mu_j^a, \mu_i^b, \mu_j^b \in \{0, \nu, \mu\}$ and hence $\mu_i^a + \mu_j^a, \mu_i^b + \mu_j^b \in \{0, \nu, \mu, 2\nu, \mu + \nu, 2\mu\}$. The probability of Alice and Bob sending out intensities $\vec{\mu}$ for the (i, j) pair can be derived from the single-round probabilities as

$$q^{\vec{\mu}} = \sum_{(\mu_i^a + \mu_j^a, \mu_i^b + \mu_j^b) = \vec{\mu}} s_{\mu_i^a} s_{\mu_j^a} s_{\mu_i^b} s_{\mu_j^b}, \tag{53}$$

which is independent of the pairing strategy.

In the entanglement-based mode-pairing scheme presented in Supplementary Note 2, for each pair of locations (i, j), Alice is able to perform the overall-photon-number measurement on the systems $\tilde{A}_i$ and $\tilde{A}_j$, respectively. We denote the photon-number measurement result to be k^a. Likewise, Bob can also perform the photon-number measurement on $\tilde{B}_i$ and $\tilde{B}_j$ and obtain k^b. Let $\vec{k}$ denote the photon numbers (k^a, k^b) on the (i, j) pair. Given the intensity setting $\vec{\mu}$ on the (i, j) pair, the probability for Alice and Bob's photon-number measurement result to be $\vec{k} = (k^a, k^b)$ is given by

$$\Pr(\vec{k}|\vec{\mu}) = e^{-(\mu_i^a + \mu_j^a) - (\mu_i^b + \mu_j^b)} \frac{(\mu_i^a + \mu_j^a)^{k^a} (\mu_i^b + \mu_j^b)^{k^b}}{k^a! k^b!}, \tag{54}$$

which is a product of two Poisson distributions, since the intensity settings of Alice and Bob are independent.

In the above description, Alice and Bob first choose the intensity setting $\vec{\mu}$ with probability $q^{\vec{\mu}}$ and then measure the ancillary systems to obtain the photon number $\vec{k}$ with probability $\Pr(\vec{k}|\vec{\mu})$. From another viewpoint, the *conditional*

probability of the intensity setting being $\vec{\mu}$ if Alice and Bob measure the photon number on (i,j)-pair and obtain the result $\vec{k}$ is given by

$$\Pr(\vec{\mu}|\vec{k}) = \frac{q^{\vec{\mu}}\Pr(\vec{k}|\vec{\mu})}{\sum_{\vec{\mu}'} q^{\vec{\mu}'}\Pr(\vec{k}|\vec{\mu}')}, \tag{55}$$

where the summation of $\vec{\mu}' = (\mu^a, \mu^b)$ takes over all possible source-intensity settings. The conditional probability $\Pr(\vec{\mu}|\vec{k})$ reflects an intrinsic property of the source, which is independent of the specific pairing setting $\vec{\chi}$. We remark that, the probabilities $\Pr(\vec{k}|\vec{\mu}), q^{\vec{\mu}}$ and $\Pr(\vec{\mu}|\vec{k})$ in Eq. (54) and (55) are *a prior* probability distributions without the post-selection of the emitted pulses based on Charlie's announcement $\vec{C}$.

Now, let us investigate the detection side. For an (i,j) pair, Alice and Bob determine the bases and raw key bits based on the encoded intensities and phases. If the intensity vector $\vec{\mu}$ satisfies

$$\mu_i^a\mu_j^a = \mu_i^b\mu_j^b = 0, \mu_i^a + \mu_j^a + \mu_i^b + \mu_j^b \neq 0, \tag{56}$$

then it is a Z-pair. Below, we mainly consider decoy-state estimation in the Z basis, but the analysis for X-basis estimation is similar.

After mode pairing and sifting, Alice and Bob obtain M rounds of Z-pairs with successful detections; of these, E rounds are erroneous. Let $M^{\vec{\mu}}$ denote the number of rounds detected with intensity setting $\vec{\mu}$, among which $E^{\vec{\mu}}$ rounds are wrongly detected. Let the total and erroneous numbers of rounds with photon numbers $\vec{k}$ be denoted by $M_{\vec{k}}$ and $E_{\vec{k}}$, respectively, among which $M_{\vec{k}}^{\vec{\mu}}$ and $E_{\vec{k}}^{\vec{\mu}}$ are the rounds with intensity setting $\vec{\mu}$. We have the following relations,

$$\begin{aligned} M &= \sum_{\vec{\mu}} M^{\vec{\mu}} = \sum_{\vec{k}} M_{\vec{k}} = \sum_{\vec{\mu}}\sum_{\vec{k}} M_{\vec{k}}^{\vec{\mu}} \\ E &= \sum_{\vec{\mu}} E^{\vec{\mu}} = \sum_{\vec{k}} E_{\vec{k}} = \sum_{\vec{\mu}}\sum_{\vec{k}} E_{\vec{k}}^{\vec{\mu}}. \end{aligned} \tag{57}$$

In the entire QKD process, the values $\{M^{\vec{\mu}}, E^{\vec{\mu}}\}_{\vec{\mu}}$ are known to Alice and Bob. The values $\{M_{\vec{k}}$ and $E_{\vec{k}}\}_{\vec{k}}$, however, are unknown to them but are fixed after Charlie's announcement.

We remark that, no matter what the intensity setting $\vec{\mu}$ is, if Alice and Bob measures the photon number on the ancillary systems and obtain the result $\vec{k}$, the post-selected state is independent of the intensity setting. As a result, *conditional on Alice and Bob's specific photon-number measurement result $\vec{k}$, Charlie's announcement strategy is independent of the intensity setting $\vec{\mu}$.* This is an alternative description of the basic assumption that the original decoy-state method makes in its security proof [10].

Based on this property, in all the generated Z-pairs with the photon-number measurement result to be $\vec{k}$, the expected ratio of different intensity settings should be the same as the ratio of the emitted states,

$$\frac{M_{\vec{k}}^{\vec{\mu}}}{M_{\vec{k}}^{\vec{\mu}'}} \approx \frac{\Pr(\vec{\mu},\vec{k})}{\Pr(\vec{\mu}',\vec{k})} = \frac{q^{\vec{\mu}}\Pr(\vec{k}|\vec{\mu})}{q^{\vec{\mu}'}\Pr(\vec{k}|\vec{\mu}')}, \tag{58}$$

where the approximation is due to statistical fluctuation. In the limit of infinite data size, the approximation becomes an equality.

In our experiment, we assume that Alice and Bob randomly determine the intensity $\vec{\mu}$ at each location by measuring an ancillary system. Measurement results are used to classically control the intensity of the signals to Charlie. In practice, Alice and Bob first measure the overall photon number and obtain $\vec{k}$. In this case, the intensity choice is determined only by $\vec{k}$, independent of Charlie's announcement. Among the $M_{\vec{k}}$ pairs with photon number $\vec{k}$, the number of pairs with an intensity setting $\vec{\mu}$ is a variable $\mathcal{M}_{\vec{k}}^{\vec{\mu}}$ that is determined by Alice and Bob's ancillary systems. The expected ratio of the intensity setting $\vec{\mu}$ is given by

$$\mathbb{E}\left(\frac{\mathcal{M}_{\vec{k}}^{\vec{\mu}}}{M_{\vec{k}}}\right) = \Pr(\vec{\mu}|\vec{k}) = \frac{q^{\vec{\mu}}\Pr(\vec{k}|\vec{\mu})}{\sum_{\vec{\mu}'} q^{\vec{\mu}'}\Pr(\vec{k}|\vec{\mu}')}, \tag{59}$$

where the expectation value is taken for the random variable $\mathcal{M}_{\vec{k}}^{\vec{\mu}}$ characterising Alice and Bob's intensity settings. Similarly, for erroneous detection among the $E_{\vec{k}}$ pairs with $\vec{k}$ photon numbers, $\mathcal{E}_{\vec{k}}^{\vec{\mu}}$ is the variable for describing the

number of pairs with intensity setting $\vec{\mu}$,

$$\mathbb{E}\left(\frac{\mathcal{E}_{\vec{k}}^{\vec{\mu}}}{E_{\vec{k}}}\right) = \Pr(\vec{\mu}|\vec{k}) = \frac{q^{\vec{\mu}}\Pr(\vec{k}|\vec{\mu})}{\sum_{\vec{\mu}'} q^{\vec{\mu}'}\Pr(\vec{k}|\vec{\mu}')}. \tag{60}$$

From Eqs. (59) and (60), we have,

$$\begin{aligned}\mathbb{E}[\mathcal{M}_{\vec{k}}^{\vec{\mu}}] &= \Pr(\vec{\mu}|\vec{k})M_{\vec{k}},\\ \mathbb{E}[\mathcal{E}_{\vec{k}}^{\vec{\mu}}] &= \Pr(\vec{\mu}|\vec{k})E_{\vec{k}}.\end{aligned} \tag{61}$$

Let the composite variables $\mathcal{M}^{\vec{\mu}}$ and $\mathcal{E}^{\vec{\mu}}$ denote the overall numbers of pairs with intensity settings $\vec{\mu}$,

$$\begin{aligned}\mathcal{M}^{\vec{\mu}} &= \sum_k \mathcal{M}_{\vec{k}}^{\vec{\mu}},\\ \mathcal{E}^{\vec{\mu}} &= \sum_k \mathcal{E}_{\vec{k}}^{\vec{\mu}}.\end{aligned} \tag{62}$$

From Eqs. (61) and (62), we have

$$\begin{aligned}\mathbb{E}[\mathcal{M}^{\vec{\mu}}] &= \sum_{\vec{k}} \Pr(\vec{\mu}|\vec{k})M_{\vec{k}}\\ \mathbb{E}[\mathcal{E}^{\vec{\mu}}] &= \sum_{\vec{k}} \Pr(\vec{\mu}|\vec{k})E_{\vec{k}}.\end{aligned} \tag{63}$$

To summarise, decoy-state analysis can be formulated as follows. *For unknown $\{M_k, E_k\}_k$ and known $\{\Pr(\vec{\mu}|\vec{k})\}_{\vec{\mu},\vec{k}}$, the lower bound of $\mathcal{M}_{(1,1)}^{(\mu,\mu)}$ and the upper bound of $\mathcal{E}_{(1,1)}^{(\mu,\mu)}$ are evaluated given the observed constraints of the detected pair numbers $\{M^{\vec{\mu}}, E^{\vec{\mu}}\}_{\vec{\mu}}$.* This problem can be solved through the following steps.

1. Record the number of clicked rounds $\{M^{\vec{\mu}}, E^{\vec{\mu}}\}_{\vec{\mu}}$.

2. Evaluate the upper and lower bounds of the expectation values $\left\{\mathbb{E}\left[\mathcal{M}^{\vec{\mu}}\right], \mathbb{E}\left[\mathcal{E}^{\vec{\mu}}\right]\right\}_{\vec{\mu}}$ using the (inverse) Chernoff bound for independent variables.

3. Solve the following linear-programming problems:

$$\begin{aligned}\min \quad & M_{(1,1)}\\ \text{s.t.} \quad & \mathbb{E}^L\left[\mathcal{M}^{\vec{\mu}}\right] \le \sum_{\vec{k}} \Pr(\vec{\mu}|\vec{k})M_{\vec{k}} \le \mathbb{E}^U\left[\mathcal{M}^{\vec{\mu}}\right], \quad \forall\vec{\mu},\\ & 0 \le M_{\vec{k}} \le M,\end{aligned} \tag{64}$$

$$\begin{aligned}\max \quad & E_{(1,1)}\\ \text{s.t.} \quad & \mathbb{E}^L\left[\mathcal{E}^{\vec{\mu}}\right] \le \sum_{\vec{k}} \Pr(\vec{\mu}|\vec{k})E_{\vec{k}} \le \mathbb{E}^U\left[\mathcal{E}^{\vec{\mu}}\right], \quad \forall\vec{\mu},\\ & 0 \le E_{\vec{k}} \le E,\end{aligned} \tag{65}$$

where $\vec{\mu} \in \{0, \nu, \mu\}^{\otimes 2}$, $\vec{k} \in \mathbb{Z}_+^{\otimes 2}$ and $\mathbb{Z}_+ = \{0, 1, 2, ...\}$ is the set of natural numbers. Denote the results for these two programmes as $M_{(1,1)}^L$ and $E_{(1,1)}^U$, respectively.

4. To evaluate the upper bound of $M_{(1,1)}^{(\mu,\mu)}$ and the lower bound of $E_{(1,1)}^{(\mu,\mu)}$ using the values of $M_{(1,1)}^L$ and $E_{(1,1)}^U$ with Eq. (61), one can apply the Chernoff bound again for independent variables.

Here, we explain why Alice and Bob can use the Chernoff bound for independent variables in Step 2 and 4. As we mentioned before Eq. (58), when the photon number $\vec{k}$ on the pair of location is given, the intensity $\vec{\mu}$ for each pair of locations is determined merely by the conditional probability $\Pr(\vec{\mu}|\vec{k})$ in Eq. (61). As a result, the intensity choices

in different pair of locations are independent when the photon number $\vec{k}$ of the pairs are given. Moreover, when the emitted state in each round is identical, the intensity choices in different pairs of locations are also identical. To be more clear, we consider all the $M_{\vec{k}}$ clicked pairs for the pairs with specific measured photon number $\vec{k}$. If Alice and Bob check the intensity label $\vec{\mu}$ on these pairs, and treat these intensity labels as random variables, then these varibles are i.i.d.,

$$\hat{\vec{\mu}}_i = \vec{\mu}, \text{ with probability } \Pr(\vec{\mu}|\vec{k}). \quad (i = 1, 2, ..., M_{\vec{k}}) \tag{66}$$

Here, the value of the conditional probability $\Pr(\vec{\mu}|\vec{k})$ only depends on the property of the source. In this case, Alice and Bob can evaluate the real number of pairs $M_{\vec{k}}^{\vec{\mu}}$ from its expectation value $M_{\vec{k}} \Pr(\vec{\mu}|\vec{k})$ using Chernoff bound of independent variables (Step 4 in the decoy estimation procedure above, cf. Eq. (33) in Ref. [11]); on the other hand, if Alice and Bob were awared of the photon number measurement results on the pairs, they can infer the expectation value from the real number of pairs $M_{\vec{k}}^{\vec{\mu}}$ by the inverse Chernoff bound (Step 2 in the decoy estimation procedure above, cf. Eq. (30) in Ref. [11]).

In the practical Step 2, however, Alice and Bob do not have the photon numer measurement results on the pairs. As an alternative way, instead of solving the inverse Chernoff problem for each photon number setting $\vec{k}$, they can combine the estimation problems together: to infer the expectation value of $\mathcal{M}^{\vec{\mu}} := \sum_{\vec{k}} \mathcal{M}_{\vec{k}}^{\vec{\mu}}$ based on the observed value of $M^{\vec{\mu}}$. When the underlying values the photon numbers $\vec{k}$ for each pair of locations are determined, the intensity variables $\hat{\vec{\mu}}_i$ are independent, following Eq. (66), regardless of the photon number $\vec{k}$. For different $\vec{k}$, however, the variables $\hat{\vec{\mu}}_i$ are not identical anymore, as the conditional probabilities $\Pr(\vec{\mu}|\vec{k})$ differ. In this case, we can still apply the inverse Chernoff bound for the independent variables.

We have introduced the decoy-state procedure for the Z-basis. Likewise, Alice and Bob can perform decoy-state analysis for the sifted X-basis obtain the single-photon-pair numbers $M_{(1,1)}^{X,L}$ and $E_{(1,1)}^{X,U}$ for successful and erroneous detection, respectively. Let $M_{(1,1)}^{(\mu,\mu),Z,L}$ denote the estimated lower bound on the single-photon-pair number with intensity $(\mu^a, \mu^b) = (\mu, \mu)$ in the case where Alice and Bob emit Z-basis data. The finite key-length formula of the mode-pairing scheme is given by

$$\begin{aligned} M_R &= M^{(\mu,\mu),Z} \left\{ q_{(1,1)}^{(\mu,\mu),Z,L} \left[1 - H\left(e_{(1,1),ph}^{(\mu,\mu),Z,U} \right) \right] - fH(E_{\mu\mu}^Z) \right\}, \\ &= M_{(1,1)}^{(\mu,\mu),Z,L} \left[1 - H\left(\frac{E_{(1,1),ph}^{(\mu,\mu),Z,U}}{M_{(1,1)}^{(\mu,\mu),Z,L}} \right) \right] - fM^{(\mu,\mu),Z} H(E_{\mu\mu}^Z), \end{aligned} \tag{67}$$

where $E_{(1,1),ph}^{(\mu,\mu),Z,U}$ is the upper bound of the phase-error number of the single-photon pair with the intensity $(\mu^a, \mu^b) = (\mu, \mu)$ when Alice and Bob emit Z-basis data. $E_{(1,1),ph}^{(\mu,\mu),Z,U}$ can be directly estimated from $M_{(1,1)}^{(\mu,\mu),Z,L}$, $M_{(1,1)}^{X,L}$ and $E_{(1,1)}^{X,U}$ based on the statistics of random sampling without a replacement problem [12]. The number of (μ, μ)-pairs in the Z-basis $M^{(\mu,\mu,Z)}$ and the quantum bit-error number $E_{\mu\mu}^Z$ can be directly obtained from the experimental results. The lower bound of the fraction $q_{(1,1)}^{(\mu,\mu),Z,L}$ and upper bound of the phase-error rate $e_{(1,1),ph}^{(\mu,\mu),Z,U}$ are defined as

$$\begin{aligned} q_{(1,1)}^{(\mu,\mu),Z,L} &:= \frac{M_{(1,1)}^{(\mu,\mu),Z,L}}{M^{(\mu,\mu),Z}}, \\ e_{(1,1),ph}^{(\mu,\mu),Z,U} &:= \frac{E_{(1,1),ph}^{(\mu,\mu),Z,U}}{M_{(1,1)}^{(\mu,\mu),Z,L}}, \end{aligned} \tag{68}$$

In the asymptotic case, $q_{(1,1)}^{(\mu,\mu),Z,L}$ and $e_{(1,1),ph}^{(\mu,\mu),Z,U}$ become q_{11} and e_{11}^X, respectively, defined in the main text.

In practice, the linear-programming problems in Eq. (64) and (65) can be analytically solved by converting the detection number $M^{\vec{\mu}}, E^{\vec{\mu}}, M_{\vec{k}}$ and $E_{\vec{k}}$ into traditional ratio values (i.e. the gain and yield). To do this, we denote the expected total pair number to be $N_p = \lfloor N/2 \rfloor$. Furthermore, we define $N^{\vec{\mu}} := q^{\vec{\mu}} N_p$ to be the expected number of rounds with intensity setting $\vec{\mu}$. We define $N_{\vec{k}}^{\infty} := \sum_{\vec{\mu}} \Pr(\vec{k}|\vec{\mu}) N^{\vec{\mu}}$ to be the expected number of rounds where the source sends out $\vec{k}$-photon states. Then the 'gain' and 'yield' are defined as

$$\begin{aligned} \mathcal{Q}^{\vec{\mu}} &:= \frac{\mathcal{M}^{\vec{\mu}}}{N^{\vec{\mu}}}, \quad \mathcal{Q}_E^{\vec{\mu}} := \frac{\mathcal{E}^{\vec{\mu}}}{N^{\vec{\mu}}}, \\ Y_{\vec{k}}^* &:= \frac{M_{\vec{k}}}{N_{\vec{k}}^{\infty}}, \quad (Y_E)_{\vec{k}}^* := \frac{E_{\vec{k}}}{N_{\vec{k}}^{\infty}}. \end{aligned} \tag{69}$$

The relationship between $\mathcal{Q}^{\vec{\mu}}$ and $Y^*_{\vec{k}}$ is,

$$\begin{aligned}
\mathbb{E}[\mathcal{Q}^{\vec{\mu}}] &= \mathbb{E}\left[\frac{\mathcal{M}^{\vec{\mu}}}{N^{\vec{\mu}}}\right] = \frac{\mathbb{E}[\mathcal{M}^{\vec{\mu}}]}{N^{\vec{\mu}}} \\
&= \sum_{\vec{k}} \Pr(\vec{\mu}|\vec{k}) \frac{M_{\vec{k}}}{q^{\vec{\mu}} N_p} \\
&= \sum_{\vec{k}} \frac{q^{\vec{\mu}} \Pr(\vec{k}|\vec{\mu})}{\sum_{\vec{\mu}'} q^{\vec{\mu}'} \Pr(\vec{k}|\vec{\mu}')} \frac{M_{\vec{k}}}{q^{\vec{\mu}} N_p} \\
&= \sum_{\vec{k}} \Pr(\vec{k}|\vec{\mu}) \frac{M_{\vec{k}}}{\sum_{\vec{\mu}'} (N_p q^{\vec{\mu}'}) \Pr(\vec{k}|\vec{\mu}')} \\
&= \sum_{\vec{k}} \Pr(\vec{k}|\vec{\mu}) Y^*_{\vec{k}}, \\
\mathbb{E}[\mathcal{Q}^{\vec{\mu}}_E] &= \sum_{\vec{k}} \Pr(\vec{k}|\vec{\mu}) (Y_E)^*_{\vec{k}},
\end{aligned} \tag{70}$$

where $\Pr(\vec{k}|\vec{\mu})$ is the product of two Poisson distributions, given by Eq. (54). In this way, one can directly apply the traditional decoy-state formulas for MDI-QKD as a solution to the linear-programming problems [13, 14]. Note that the value of N_p will not affect the correctness of Eq. (70). If we redefine N_p as $N_p' = cN_p$, then the defined gain and yield will become $\frac{1}{c}\mathcal{Q}^{\vec{\mu}}$ and $\frac{1}{c}Y^*_{\vec{k}}$, respectively. From Eq. (69), we can see that the final estimated value $M_{\vec{k}}$ is invariant only if the experimental data $\{M^{\vec{\mu}}\}$ are unchanged.

Supplementary Note 4. SIMULATION FORMULAS

Here, we list the formulas used for key-rate simulation of different QKD schemes. In Supplementary Note 4 A, we derive the simulation formula for the free-round MP scheme with maximal pairing length l. In a similar fashion, we derive the simulation formula for the time-bin MDI-QKD scheme in Supplementary Note 4 B. Finally, in Supplementary Note 4 C, we list the simulation formulas used in the main text for the decoy-BB84 and PM-QKD schemes.

A. Mode-pairing scheme

The key rate of the MP scheme can be calculated using Eqs. (71), (72), (74), (79), (83), (84) and (85) below.

In the MP scheme, Alice and Bob use the Z-basis to generate keys. In the asymptotic case, we assume that Alice and Bob choose to randomly emit signals with intensity $\{0, \mu\}$ with a probability of nearly $1/2$ and a decoy intensity ν with negligible probability. Let us express the coherent pulse emitted by Alice in the i-th round as $|\sqrt{z_i^a \mu} \exp(i\phi_i^a)\rangle$, with $z_i^a \in \{0, 1\}$ being a random variable indicating the intensity and ϕ_i^a being a random phase. Similarly, Bob emits $|\sqrt{z_i^b \mu} \exp(i\phi_i^b)\rangle$ in the i-th round. The i-th round intensity setting is then denoted by a 2-bit vector $z_i := [z_i^a, z_i^b]$.

In the simulation, Alice and Bob are assumed to emit pulses to Charlie through a typical symmetric-loss channel. The single-side transmittance of the channel (with included detection efficiency η_d included) is η_s, and the dark count of each detector is p_d. The simulation data is provided in Supplementary Figure 4 in the main text. In this case, the channel is i.i.d. for each round. Alice and Bob then pair the clicked pulses and set their bases. For the (i, j)-th pulses to be paired, let $\tau_{i,j} = [\tau^a_{i,j}, \tau^b_{i,j}] := [z_i^a \oplus z_j^a, z_i^a \oplus z_j^a]$, where $\oplus$ is the bit-wise addition modulo 2. When $\tau_{i,j} = [1, 1]$, the (i, j)-pair is then set to be a signal pair.

The key rate of the MP scheme is expressed as

$$R = r_p(p, l)\, r_s \left\{ \bar{q}_{11}[1 - H(e^X_{(1,1)})] - fH(E^Z_{(\mu,\mu)}) \right\}, \tag{71}$$

where l is the maximal pairing gap, $r_p(p, l)$ is the expected pair number generated during each round, and r_s is the probability that a clicked pulse pair is a signal pair, $\bar{q}_{11}$ and $e^X_{(1,1)}$ are, respectively, the expected single-photon pair ratio in all signal pairs and the phase error of the single-photon pairs, which are estimated via decoy-state methods. f is the error-correction efficiency, $E^Z_{(\mu,\mu)}$ is the bit-error rate of the signal pairs and p is the probability of the i-th

signal to be successfully clicked (as defined in Eq. (74)). The value of $r_p(p,l)$ has already been given in the Methods as

$$r_p(p,l) = \left[\frac{1}{p[1-(1-p)^l]} + \frac{1}{p}\right]^{-1}. \tag{72}$$

Here, we first consider the calculation of $r_s, \bar{q}_{11}$ and $E^Z_{(\mu,\mu)}$. In the i-th round, we denote the click events of the left and right detectors at the i-th turn by two variables (L_i, R_i). The successful click variable is $C_i = L_i \oplus R_i$. Only when $C_i = 1$ does an successful click occur. The detection probability $\Pr(C_i = 1|z_i)$ is given by

$$\Pr(C_i = 1|z_i) \approx 1 - (1-2p_d)\exp\left[-\eta_s\mu\left(z_i^a + z_i^b\right)\right]. \tag{73}$$

The expected click probability, i.e., the total transmittance of each round, is

$$p := \Pr(C_i = 1) = \sum_{z_i} \Pr(C_i = 1|z_i)\Pr(z_i) = \frac{1}{4}\sum_{z_i} \Pr(C_i = 1|z_i). \tag{74}$$

The pairing rate $r_p(p,l)$ can then be calculated using Eq. (72).

The phase-randomised coherent states emitted in the i-th round can also be regarded as the mixture of photon-number states. $\Pr(C_i = 1|n_i)$ denotes the detection probability when Alice and Bob emit photon-number states $|n_i^a\rangle$ and $|n_i^b\rangle$, respectively; it is given by

$$\Pr(C_i = 1|n_i) \approx 1 - (1-2p_d)(1-\eta_s)^{(n_i^a + n_i^b)}. \tag{75}$$

Now, let us calculate the signal-pair ratio r_s. Without loss of generality, we consider the i-th and j-th turns as a paired group. For a general turn, the probability of a click caused by the intensity setting z is

$$\Pr(z|C=1) = \frac{\Pr(z, C=1)}{\Pr(C=1)} = \frac{\Pr(C=1|z)}{\sum_{z'}\Pr(C=1|z')}. \tag{76}$$

Note that the subscript is omitted, since the detections in all rounds are identical and independently distributed in our simulation.

In the MP scheme, an successful click happens when $\tau_{i,j} = [1,1]$. Therefore, four possible configurations of z_i and z_j (which generate signal pairs) are

$$[z_i, z_j] \in \{[00,11],[01,10],[10,01],[11,00]\}; \tag{77}$$

of these, $Err := \{[00,11],[11,00]\}$ are the two configurations that cause bit errors. To simplify our notations, we introduce several events,

$$\begin{aligned}
\Pr(C) &= \Pr(\textit{Pair Clicked}) := \Pr(C_i = C_j = 1) = p^2,\\
\Pr(E) &= \Pr(\textit{Pair Effective}) := \Pr(z_i \oplus z_j = 11),\\
\Pr(Err) &= \Pr(\textit{Pair Erroneous}) := \Pr([z_i, z_j] \in Err),\\
\Pr(S) &= \Pr(\textit{Single-photon Pair}) := \Pr(n_i \oplus n_j = 11).
\end{aligned} \tag{78}$$

The signal-pair ratio r_s is then

$$\begin{aligned}
r_s =& \Pr(E|C) = \Pr(z_i \oplus z_j = 11 | C_i = 1, C_j = 1)\\
=& \sum_{z_i \oplus z_j = 11} \Pr(z_i|C_i = 1)\Pr(z_j|C_j = 1)\\
=& \sum_{z_i \oplus z_j = 11} \frac{\Pr(C_i = 1|z_i)\Pr(z_i)}{\Pr(C_i = 1)}\frac{\Pr(C_j = 1|z_j)\Pr(z_j)}{\Pr(C_j = 1)}\\
=& \frac{1}{16}\frac{1}{p^2}\sum_{z_i \oplus z_j = 11} \Pr(C_i = 1|z_i)\Pr(C_j = 1|z_j),
\end{aligned} \tag{79}$$

We remark that, when $\eta_s\mu \ll 1$, the signal-pair ratio r_s is approximately

$$\begin{aligned}
r_s &\approx \frac{1}{16}\frac{1}{p^2}[2(\eta_s\mu)^2]\\
&= \frac{1}{16}\frac{1}{(\eta_s\mu)^2}[2(\eta_s\mu)^2] = \frac{1}{8},
\end{aligned} \tag{80}$$

which is nearly a constant independent of η_s and μ.

The expected error rate $E^Z_{(\mu,\mu)}$ of the (i,j)-pair is

$$\begin{aligned} E^Z_{(\mu,\mu)} &= \Pr\left(Err|E,C\right) \\ &= \frac{\Pr\left(Err,E|C\right)}{\Pr\left(E|C\right)} = \frac{\Pr\left(Err|C\right)}{\Pr\left(E|C\right)} \\ &= r_s^{-1}\Pr\left(Err|C\right). \end{aligned} \tag{81}$$

Note that the erroneous pair condition is contained in the effective pair condition. The erroneous probability is given by

$$\begin{aligned} &\Pr\left(Err|C\right) = \Pr\left([z_i,z_j]\in Err|C_i = C_j = 1\right) \\ &= \sum_{[z_i,z_j]\in Err} \Pr(z_i|C_i=1)\Pr(z_j|C_j=1) \\ &= \sum_{[z_i,z_j]\in Err} \frac{\Pr(C_i=1|z_i)\Pr(z_i)}{\Pr(C_i=1)}\frac{\Pr(C_j=1|z_j)\Pr(z_j)}{\Pr(C_j=1)} \\ &= \frac{1}{16}\frac{1}{p^2}\sum_{[z_i,z_j]\in Err}\Pr(C_i=1|z_i)\Pr(C_j=1|z_j). \end{aligned} \tag{82}$$

Therefore,

$$E^Z_{(\mu,\mu)} = \frac{1}{16}\frac{1}{r_s p^2}\sum_{[z_i,z_j]\in Err}\Pr(C_i=1|z_i)\Pr(C_j=1|z_j). \tag{83}$$

Then, we calculate the expected single-photon pair ratio $\bar{q}_{11}$ in the effective signal-pairs:

$$\begin{aligned} \bar{q}_{11} &= \Pr(S|E,C) = \frac{\Pr(S,E,C)}{\Pr(E,C)} \\ &= \frac{1}{r_s p^2}\Pr(S,E,C) \\ &= \frac{1}{r_s p^2}\sum_{z_i,z_j}\Pr(S,E,C|z_i,z_j)\Pr(z_i,z_j) \\ &= \frac{1}{16}\frac{1}{r_s p^2}\sum_{z_i\oplus z_j=11}\Pr(S,C|z_i,z_j) \\ &= \frac{1}{16}\frac{1}{r_s p^2}\sum_{z_i\oplus z_j=11}\Pr(C|S,z_i,z_j)\Pr(S|z_i,z_j) \\ &= \frac{1}{16}\frac{P_\mu(1)^2}{r_s p^2}\sum_{z_i\oplus z_j=11}\Pr(C_i=1|n_i=z_i)\Pr(C_j=1|n_j=z_j), \end{aligned} \tag{84}$$

where $P_\mu(k) = \exp(-\mu)\frac{\mu^k}{k!}$ is the Poisson distribution and $\Pr(C_i = 1|n_i)$ is defined in Eq. (75).

The phase-error rate formula of the MP scheme cannot be calculated for single-pulse-based methods. In the MP scheme, if the decoy-state estimation is perfect, then the phase-error rate should be the same as that of the original two-mode MDI-QKD scheme. Here, we apply the formulas (A9) and (A11) in Ref. [6]:

$$\begin{aligned} Y_{11} &= (1-p_d)^2\left[\frac{\eta_a\eta_b}{2} + (2\eta_a+2\eta_b-3\eta_a\eta_b)p_d + 4(1-\eta_a)(1-\eta_b)p_d^2\right], \\ e^X_{11}Y_{11} &= e_0 Y_{11} - (e_0-e_d)(1-p_d^2)\frac{\eta_a\eta_b}{2}. \end{aligned} \tag{85}$$

Here, $e_0 = 1/2$ is the error caused by vacuum signals, e_d is the pre-set misalignment error, and $\eta_a(\eta_b)$ are the transmittances from Alice (Bob) to Charlie. In our case, $\eta_a = \eta_b = \eta_s$.

In the MP scheme and the normal two-mode MDI-QKD schemes, since there is no common phase reference between Alice and Bob, the X-basis detection results for coherent states will have an intrinsic 25% error rate in $E^X_{\mu\mu}$, as is shown in Eq. (B15) in Ref. [6]. Fortunately, if we can perform an accurate decoy-state estimation, we can estimate single-photon error rate e^X_{11} accurately without this 25% effect.

B. Time-bin MDI-QKD

The key rate of time-bin MDI-QKD is calculated as Eqs. (86), (91), (92), (93), and (94) below.

The time-bin MDI-QKD scheme can be regarded as a special case of the MP scheme with fixed-pairing setting and a predetermined effective-intensity setting $[z_i, z_j]$ at locations i and j. That is, if the pairs are clicked, then the pair is effective with $z_i \oplus z_j = 11$. The derivation of the simulation formulas for time-bin MDI-QKD is similar to that of the MP scheme in Supplementary Note 4 A.

In the time-bin MDI-QKD scheme, Alice and Bob use the Z-basis to generate keys. In the asymptotic case, we assume that they choose to emit the X-basis state with a negligible probability, such that the basis-sifting factor is almost 1. The key rate of the time-bin MDI-QKD scheme is

$$R_{MDI} = \frac{1}{2} Q_{\mu\mu} \{ \bar{q}_{11} [1 - H(e^X_{(1,1)})] - f H(E^Z_{(\mu,\mu)}) \}, \tag{86}$$

where the factor $\frac{1}{2}$ is for a comparison with the MP scheme, since the key-rate formula of R_{MDI} is defined on a pair of rounds while the key-rate formula Eq. (71) for the MP scheme is defined on each round. $Q_{\mu\mu}$ is the probability of the pair with successful detection. The parameters $\bar{q}_{11}, e^X_{(1,1)}$ and $E^Z_{(\mu,\mu)}$ are similar to those in the MP scheme.

Similar to Supplementary Note 4 A, we also introduce $z_i = [\zeta_i^a, z_i^b]$ and C_i to denote the intensity setting of the i-th location and detection results, respectively. The single-location detection probability of a given z_i is the same as that in the MP scheme,

$$\Pr(C_i = 1 | z_i) \approx 1 - (1 - 2p_d) \exp\left[-\eta_s \mu \left(z_i^a + z_i^b\right)\right]. \tag{87}$$

Meanwhile, we introduce $n_i = [n_i^a, n_i^b]$ to denote the emitted photon number at the i-th location. The conditional detection probability $\Pr(C_i = 1|n_i)$ is still given by

$$\Pr(C_i = 1 | n_i) \approx 1 - (1 - 2p_d)(1 - \eta_s)^{(n_i^a + n_i^b)}. \tag{88}$$

In time-bin MDI-QKD and for a fixed pair of locations i and j, the configurations of z_i and z_j are predetermined to be chosen in the set

$$[z_i, z_j] \in \{[00, 11], [01, 10], [10, 01], [11, 00]\}. \tag{89}$$

Among these $Err := \{[00, 11], [11, 00]\}$ are the two configurations causing bit errors. To simplify our notation, we also introduce several events,

$$\begin{aligned} \Pr(C) &= \Pr(\textit{Pair Clicked}) := Q_{\mu\mu}, \\ \Pr(Err) &= \Pr(\textit{Pair Erroneous}) := \Pr([z_i, z_j] \in Err), \\ \Pr(S) &= \Pr(\textit{Single-photon Pair}) := \Pr(n_i \oplus n_j = 11). \end{aligned} \tag{90}$$

By definition we have

$$\begin{aligned} Q_{\mu\mu} = \Pr(C) &= \sum_{z_i \oplus z_j = 11} \Pr(z_i, z_j) \Pr(C_i = 1|z_i) \Pr(C_j = 1|z_j) \\ &= \frac{1}{4} \sum_{z_i \oplus z_j = 11} \Pr(C_i = 1|z_i) \Pr(C_j = 1|z_j). \end{aligned} \tag{91}$$

The quantum bit-error rate $E^Z_{(\mu,\mu)}$, is

$$\begin{aligned} E^Z_{(\mu,\mu)} = \Pr(Err|C) &= \frac{\Pr(Err, C)}{\Pr(C)} \\ &= \frac{1}{Q_{\mu\mu}} \sum_{[z_i, z_j] \in Err} \Pr(z_i, z_j) \Pr(C_i = 1|z_i) \Pr(C_j = 1|z_j) \\ &= \frac{1}{4} \frac{1}{Q_{\mu\mu}} \sum_{[z_i, z_j] \in Err} \Pr(C_i = 1|z_i) \Pr(C_j = 1|z_j). \end{aligned} \tag{92}$$

The ratio of the single-photon pairs in the signals with successful detection $\bar{q}_{11}$ is

$$\begin{aligned}
\bar{q}_{11} &= \Pr(S|C) = \frac{\Pr(S,C)}{\Pr(C)} \\
&= \frac{1}{Q_{\mu\mu}} \sum_{z_i \oplus z_j = 11} \Pr(z_i, z_j) \Pr(S, C|z_i, z_j) \\
&= \frac{1}{4} \frac{1}{Q_{\mu\mu}} \sum_{z_i \oplus z_j = 11} \Pr(C|S, z_i, z_j) \Pr(S|z_i, z_j) \\
&= \frac{1}{4} \frac{P_\mu(1)^2}{Q_{\mu\mu}} \sum_{z_i \oplus z_j = 11} \Pr(C_i = 1|n_i = z_i) \Pr(C_j = 1|n_j = z_j),
\end{aligned} \tag{93}$$

where $P_\mu(k) = \exp(-\mu)\frac{\mu^k}{k!}$ is the Poisson distribution and $\Pr(C_i = 1|n_i)$ is defined in Eq. (88).

The phase-error-rate formula of time-bin MDI-QKD is given by the formulas (A9) and (A11) in Ref. [6],

$$\begin{aligned}
Y_{11} &= (1-p_d)^2 \left[\frac{\eta_a \eta_b}{2} + (2\eta_a + 2\eta_b - 3\eta_a\eta_b)p_d + 4(1-\eta_a)(1-\eta_b)p_d^2 \right], \\
e_{11}^X &= e_0 Y_{11} - (e_0 - e_d)(1-p_d^2)\frac{\eta_a\eta_b}{2}.
\end{aligned} \tag{94}$$

Here, $e_0 = 1/2$ is the error caused by vacuum signals, e_d is the pre-set misalignment error, and $\eta_a(\eta_b)$ are the transmittances from Alice (Bob) to Charlie. In our case, $\eta_a = \eta_b = \eta_s$.

C. Other QKD schemes

Here, we list the simulation formulas for decoy-state BB84 and PM-QKD which are used in the key rate simulation programme. We also list the repeaterless rate-transmittance bound drawn in the main text.

The key rate of the decoy-state BB84 scheme is [10]

$$R_{BB84} = \frac{1}{2} Q_\mu \{-fH(E_\mu^Z) + q_1[1 - H(e_1^X)]\}. \tag{95}$$

Here, for a fair comparison with the time-bin MDI-QKD and the MP scheme, we consider a time-bin encoding BB84 scheme, where Alice's Z-basis encoding is undertaken by choosing to emit a phase-randomised coherent state in one of two time-bin optical modes. In this case, the Z-basis bit error is merely caused by the dark counts, regardless of the misalignment error. We now consider an efficient basis choice with a sifting factor of 1. For a fair comparison, we multiply the original key rate of decoy-state BB84 by a factor of 1/2. The reason is that we are considering the averaged key rate generated by each pair of optical modes, A_i and B_i, while the key rate for the original decoy-state BB84 is found for each round in which Alice (and Bob) emit two optical modes.

In the simulation, the yield and error rates of the k-photon component in the X-basis are [15]

$$\begin{aligned}
Y_k &= 1 - (1 - Y_0)(1 - \eta)^k, \\
e_k &= e_d + \frac{(e_0 - e_d)Y_0}{Y_k},
\end{aligned} \tag{96}$$

where e_d is the intrinsic misalignment-error rate caused by a phase-reference mismatch. The gain and QBER of the Z-basis pulse are given by

$$\begin{aligned}
Q_\mu &= \sum_{k=0}^{\infty} \frac{\mu^k e^{-\mu}}{k!} Y_k, \\
&= 1 - (1 - Y_0)e^{-\eta\mu}, \\
E_\mu &= \frac{e_d Y_0}{Q_\mu},
\end{aligned} \tag{97}$$

where $Y_0 = 2p_d$ and $e_0 = 1/2$.

For PM-QKD, the key-rate formula is [2, 16],

$$R_{PM} = \frac{2Q_\mu}{D}\sum_j \left[1 - H(E_\mu^X) - fH(E_{\mu,j}^Z)\right], \tag{98}$$

where $D = 16$ is the number of slices. The gain, yield and error rates are

$$\begin{aligned} Q_\mu &= 1 - (1-2p_d)e^{-\eta\mu}, \\ Y_k &= 1 - (1-2p_d)(1-\eta)^k, \\ q_k &= Y_k \frac{e^{-\mu}\mu^k/k!}{Q_\mu}, \\ E_\mu^X &= 1 - \sum_k q_{2k+1} < 1 - q_1 - q_3 - q_5, \\ E_{\mu,j}^Z &= [p_d + \eta\mu(e_{\delta,j} + e_d^{PM})]\frac{e^{-\eta\mu}}{Q_\mu}, \\ e_{\delta,j} &= \sin^2\left[\frac{\pi}{4} - \left|\frac{\pi}{4} - \frac{\pi j}{D}\right|\right], \end{aligned} \tag{99}$$

where e_d^{PM} is the intrinsic misalignment error of the PM-QKD. We take these formulas from Eqs. (8)-(12) and (D1)-(D4) in Ref. [2].

For SNS-TF-QKD, the key-rate formula is [17, 18],

$$R_{SNS} = 2p_{z0}(1-p_{z0})\mu_z e^{-\mu_z} s_1 \left[1 - H(e_1^{ph})\right] - fS_z H(E^Z), \tag{100}$$

where p_{z0} is Alice's (or Bob's) probability of sending vaccum state, μ_z is the light intensity for Alice or Bob's signal pulses, s_1 is the detecting probability of single-photon signals, e_1^{ph} is the single-photon phase error rate, S_z is the detecting probability of signal lights and E^Z is the quantum bit error rate. p_{z0} and μ_z need to be optimized. We use the following simulation formula, matching the results in Ref. [18],

$$\begin{aligned} s_1 &= (1-\eta)2p_d(1-p_d) + \eta(1-p_d), \\ e_1^{ph} &= (1-e_d^{SNS})\frac{(1-\eta)p_d(1-p_d)}{s_1} + e_d^{SNS}\left[1 - \frac{(1-\eta)p_d(1-p_d)}{s_1}\right], \\ S_z &= p_{z0}^2 S_{00} + (1-p_{z0})^2 S_{22} + 2p_{z0}(1-p_{z0})S_{02}, \\ E^Z &= \left[p_{z0}^2 S_{00} + (1-p_{z0})^2 S_{22}\right]/S_z. \end{aligned} \tag{101}$$

Here, e_d^{SNS} is the misalignment error. S_{00}, S_{02}, and S_{22} indicate the detecting probabilities of the signals when Alice and Bob emit light with intensity $(0,0)$, $(0,\mu_z)$, and (μ_z,μ_z), respectively, which are given by the following formulas,

$$\begin{aligned} S_{00} &= 2p_d(1-p_d), \\ S_{02} &= 2\left[(1-p_d)e^{-\eta\mu_z/2} - (1-p_d)^2 e^{-\eta\mu_z}\right], \\ S_{22} &= 2\left[(1-p_d)e^{-\eta\mu_z} I_0(\eta\mu_z) - (1-p_d)^2 e^{-2\eta\mu_z}\right], \end{aligned} \tag{102}$$

where $I_0(x)$ is the modified Bessel function of the first kind.

The repeaterless rate-transmittance bound of the repeaterless point-to-point QKD scheme is the PLOB bound [19]

$$R_{PLOB} = -\log_2(1-\eta). \tag{103}$$

Supplementary Note 5. ADDITIONAL NUMERICAL RESULTS

To further understand the key rate property of the MP scheme, we simulate this scheme's optimal intensity settings, as well as those of the other schemes mentioned in the main text. We choose exactly the same simulation parameters as used in Fig. 4 in the main text. Supplementary Figure 20 illustrates the optimal intensities of different QKD

schemes; Supplementary Figure 19 illustrates those corresponding to different communication distances and pairing lengths l.

We first note that, for the time-bin-encoding version of BB84, the two-mode MDI-QKD and the MP scheme, the Z-basis bit error is almost 0 and is irrelevant to the intensity μ. In this case, the error-correction-related term has a negligible contribution in the key rate. For BB84, the optimal intensity satisfies (cf. Eq. (12) in Ref. [20])

$$(1-\mu)e^{-\mu} = \frac{fH(E_{\mu}^{Z})}{1-H(e_{1}^{X})}. \tag{104}$$

When $fH(E_{\mu}^{Z}) \to 0$, we have $(1-\mu)e^{-\mu} \to 0$; hence, $\mu \to 1$. The optimal μ for MDI-QKD can be derived in a similar fashion. As shown in Supplementary Figure 20, before the error rates caused by the dark-count events come to dominate the bit-error rate E^{Z}, the optimal μ values for BB84 and MDI-QKD are always 1.

The optimal μ for the MP scheme, however, cannot be directly derived from the key-rate formula due to an additional dependence of the key rate R on the pairing rate r_p. If we set $p_d \to 0$ and assume that $\eta_s\mu \ll 1$, the key rate of the MP scheme in Eq. (71) is approximately

$$\begin{aligned} R &= r_p(p,l) r_s \{q_{(1,1)}[1-H(e_{(1,1)}^{X})] - fH(E_{(\mu,\mu)}^{Z})\} \\ &\approx p\left[\frac{1}{1-(1-p)^{l}}\right]^{-1} \frac{1}{8}\{e^{-2\mu}[1-H(e_{(1,1)}^{X})] - fH(E_{(\mu,\mu)}^{Z})\}. \end{aligned} \tag{105}$$

Here,

$$\begin{aligned} r_s &\approx \frac{1}{16}\frac{1}{p^2}[2(\eta_s\mu)^2] = \frac{1}{8}, \\ q_{(1,1)} &\approx \frac{1}{16}\frac{(\mu e^{-\mu})^2}{r_s(\eta_s\mu)^2} 2\eta_s^2 = e^{-2\mu}. \end{aligned} \tag{106}$$

Now, we consider two extreme cases: 1) l is small, such that $pl \ll 1$; 2) l is large, such that $pl \gg 1$. Note that, we always assume that $1 \gg p \gg p_d$.

When $pl \ll 1$, we have $(1-p)^l \approx 1 - lp$. The key rate can be simplified to be

$$\begin{aligned} R &\approx \frac{1}{8} lp^2 \{e^{-2\mu}[1-H(e_{(1,1)}^{X})] - fH(E_{(\mu,\mu)}^{Z})\}, \\ &= \frac{1}{8} l(\eta_s\mu)^2 \{e^{-2\mu}[1-H(e_{(1,1)}^{X})] - fH(E_{(\mu,\mu)}^{Z})\}. \end{aligned} \tag{107}$$

Taking the derivative of R with respective to μ, we obtain the following condition for optimal μ:

$$(1-\mu)e^{-2\mu} = \frac{fH(E_{(\mu,\mu)}^{Z})}{1-H(e_{(1,1)}^{X})}. \tag{108}$$

Suppose that $E_{(\mu,\mu)}^{Z} \to 0$; then $\mu \to 1$. In the long-distance regime, $E_{(\mu,\mu)}^{Z}$ gets larger, resulting in a smaller μ.

When $pl \gg 1$, we have $(1-p)^l \approx 0$. The key rate can be simplified as

$$\begin{aligned} R &\approx \frac{1}{16} p \{e^{-2\mu}[1-H(e_{(1,1)}^{X})] - fH(E_{(\mu,\mu)}^{Z})\}, \\ &= \frac{1}{16} \eta_s\mu \{e^{-2\mu}[1-H(e_{(1,1)}^{X})] - fH(E_{(\mu,\mu)}^{Z})\}. \end{aligned} \tag{109}$$

Taking the derivative of R with respective to μ, we obtain the following condition for optimal μ:

$$(1-2\mu)e^{-2\mu} = \frac{fH(E_{(\mu,\mu)}^{Z})}{1-H(e_{(1,1)}^{X})}. \tag{110}$$

Suppose $E_{(\mu,\mu)}^{Z} \to 0$; then $\mu \to 1/2$. In the long-distance regime, $E_{(\mu,\mu)}^{Z}$ increases, resulting in a smaller μ.

In the general case, for a fixed l, the value of pl grows smaller as the communication distance becomes longer. The optimal value of μ may first be close to 0.5 and then increased to nearly 1.

To demonstrate the robustness of the key rate of the MP scheme against the misalignment error, we further simulate the key rate of the MP scheme under different misalignment error rates 5%, 10%, and 15%. The results show that the MP scheme is highly robust against the misalignment error. Even with a high misalignment error 15%, it can surpass the PLOB bound with a maximum pairing interval of $l = 2000$.

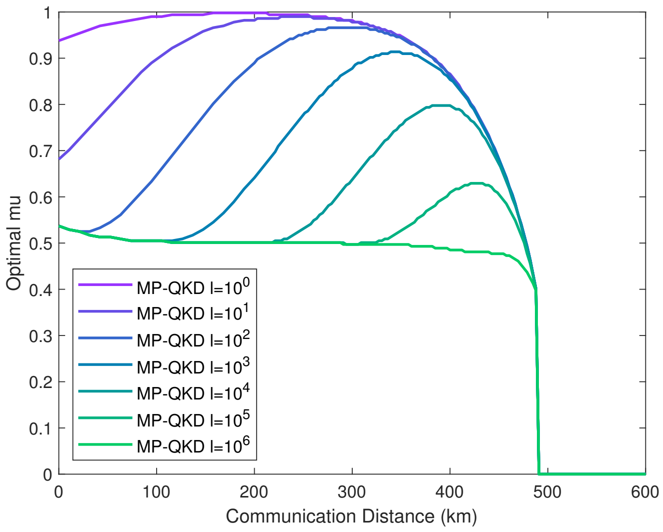

Supplementary Figure 19. Optimal intensity setting of the MP scheme with different pairing lengths l. Here, all of the simulation parameters are chosen to be the same as the ones in Fig. 4 in the main text.

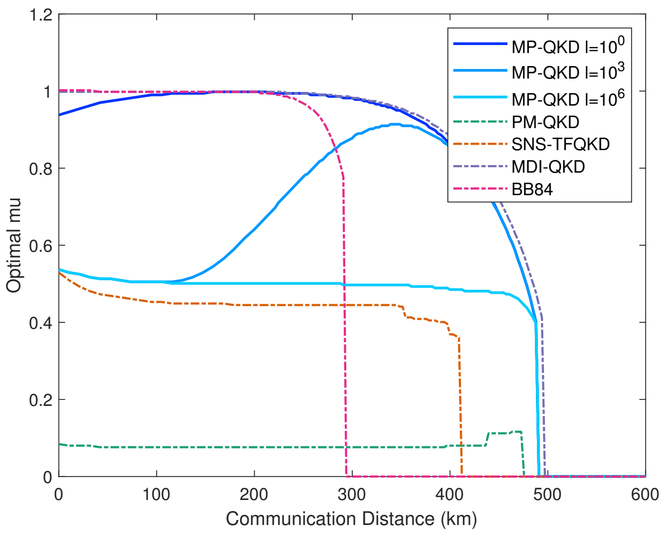

Supplementary Figure 20. Comparison of the optimal intensity settings of different QKD schemes. Here, all the simulation parameters are chosen to be the same as those in Fig. 4 in the main text. Here, the optimal μ for the BB84 scheme is defined to be the overall intensity of two emitted modes in each round. The optimal μ value for (two-mode) MDI-QKD is defined as the overall intensity of Alice's (or Bob's) two emitted modes in each round. The optimal μ values for MP-QKD, PM-QKD, and SNS-TFQKD are defined on each single round.

Supplementary Note 6. PROOF-OF PRINCIPLE EXPERIMENTAL DEMONSTRATION

In the mode-pairing scheme, like other phase-encoding QKD schemes, certain "local phase stabilization" is still required. In this section, we want to clarify the difference between this local phase stabilization and the "global phase locking" over two remote independent lasers required for the one-mode MDI-QKD schemes.

Suppose Alice and Bob hold two independent lasers emitting the optical pulses independently. One can track the phases of these coherent state pulses. For the pulses emitted by Alice and Bob at the time t, denote the global phases of the pulses as $\phi^a(t)$ and $\phi^b(t)$, respectively. Generally, the circular frequencies of Alice's and Bob's lasers, $\omega_a(t)$ and

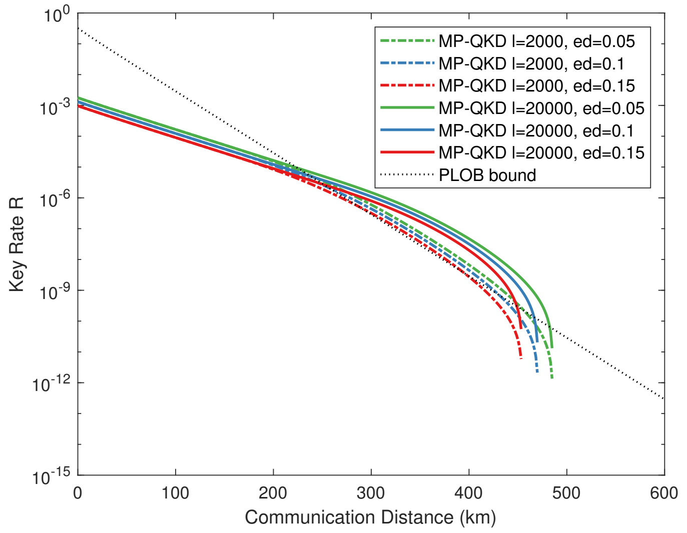

Supplementary Figure 21. Comparison of the key-rate performance of the mode-pairing scheme under different misalignment error.

$\omega_b(t)$, are changing over time. We model the phases $\phi^a(t)$ and $\phi^b(t)$ as follows,

$$\begin{aligned} \phi^a(t) &= \int_0^t \omega_a(t)dt + \psi_a(t), \\ \phi^b(t) &= \int_0^t \omega_b(t)dt + \psi_b(t), \end{aligned} \tag{111}$$

where $\psi_a(t)$ and $\psi_b(t)$ are two randomly fluctuated noise terms with a rate much slower than the one caused by circular frequencies and independent of ω_a and ω_b. Here, the phase fluctuation caused by fibres is normally slow, based on the former experimental result. When the fibre length is around 500 km, the phase drift velocity is less than 10 rad/ms [21]. Then, we define global phase locking and local phase stabilization as following.

Definition 6 (Global phase locking)**.** *After Alice and Bob perform global phase locking, they can achieve either of the following two tasks during the whole process of the experiment.*

- *They keep the relative phase between the two emitted pulses $\phi^b(t) - \phi^a(t)$ to be a constant, independent of the time t.*
- *They are able to estimate the phase difference $\phi^b(t) - \phi^a(t)$ for every moment t accurately.*

If we want to perform global phase locking, we have to stabilize the phase difference $\phi^b(t) - \phi^a(t)$ for each moment t or accurately estimate it. Due to the large frequency difference $\omega_b(t) - \omega_a(t)$ of two independent lasers, the value of $\phi^b(t) - \phi^a(t)$ changes rapidly with respect to t, making the stabilization or accurate estimation of $\phi^b(t) - \phi^a(t)$ challenging.

Definition 7 (Local phase stabilization)**.** *After Alice and Bob perform local phase locking, they can achieve either of the following two tasks for any two moments t_1 and t_2, satisfying $|t_1 - t_2| \leq \Delta t$,*

- *Alice keeps the relative phase between her two emitted pulses $\phi^a(t_2) - \phi^a(t_1)$ to be a constant, independent of t_1 and t_2. Bob does the same thing for $\phi^b(t_2) - \phi^b(t_1)$.*
- *They are able to estimate the phase difference $\theta^\delta(t_1, t_2) := \phi^b(t_2) - \phi^b(t_1) - \phi^a(t_2) + \phi^a(t_1)$ accurately.*

We remark that, the core difference of the local phase stabilization from the global phase locking is to estimate the *difference* of the phase $\phi^b(t) - \phi^a(t)$ between two moments t_1 and t_2 instead of estimating the phase itself. We now discuss why this task will be easier than the global phase locking for a reasonable time period $\Delta t = t_2 - t_1$.

In the task of local phase stabilization, the users try to stabilize

$$\theta^\delta(t_1,t_2) = [\phi^b(t_2)-\phi^b(t_1)] - [\phi^a(t_2)-\phi^a(t_1)] = \int_{t_1}^{t_2}[\omega_b(t)-\omega_a(t)]dt + [\psi_b(t_2)-\psi_b(t_1)] - [\psi_a(t_2)-\psi_a(t_1)] \quad (112)$$

within time $\Delta t = t_2 - t_1$. For a short time period Δt, $\psi_a(t_2)-\psi_a(t_1)$ and $\psi_b(t_2)-\psi_b(t_1)$ will be relatively small. To estimate the value of $\theta^\delta(t_1,t_2)$ in a short period Δt, Alice and Bob only need to track the frequency difference $\omega_b(t)-\omega_a(t)$, which is easier than directly measure the phase difference $\phi^b(t)-\phi^a(t)$. Then, they use the integration $\int_{t_1}^{t_2}[\omega_b(t)-\omega_a(t)]dt$ to estimate the phase difference $\theta^\delta(t_1,t_2)$. On the other hand, when the time period Δt gets longer, the accuracy of integration $\int_{t_1}^{t_2}[\omega_b(t)-\omega_a(t)]dt$ will be affected, and the slow phase fluctuation $\psi_a(t_2)-\psi_a(t_1)$ and $\psi_b(t_2)-\psi_b(t_1)$ would come into play, which would affect the final results of local phase stabilization. The hardness of local phase stabilization requirement depends on the time interval Δt. We now consider the following two extreme cases.

1. When $\Delta t = t_2 - t_1 \ll 1/\Delta f$, where Δf is the laser linwidth. For example, in the regular phase-encoding MDI-QKD, the Δt of local phase stabilization requirement is the time between two adjacent pulses, which is usually in this scenario. In this case, the values of $\omega_b(t)-\omega_a(t)$ is stable, and the value of $\psi_a(t_2)-\psi_a(t_1)$ and $\psi_b(t_2)-\psi_b(t_1)$ will be close to zero. As a result, the local phase stabilization is easy to realize.

2. When $\Delta t = t_2 - t_1 \gg 1/\Delta f$. Then, the frequency difference $\omega_b(t)-\omega_a(t)$ fluctuates randomly, and the values of $\psi_a(t_2)-\psi_a(t_1)$ and $\psi_b(t_2)-\psi_b(t_1)$ become large. To fulfill the requirements in Definition 7, we have to estimate both the phases $\phi^b(t_1)-\phi^a(t_1)$ and $\phi^b(t_2)-\phi^a(t_2)$ accurately. In this case, the local phase stabilization approaches the case of global phase locking.

In order to show the feasibility of realizing the local phase stabilization in Definition 7 with a reasonable large time period Δt, we perform an experimental demonstration using two independent lasers. This is the essentially the same setup as the phase-matching experiment [21], except for the removal of the strong master laser for global phase-locking. Alice and Bob each use a laser with the central frequency of $\omega_a \approx \omega_b \approx 193.533$ THz. The linewidth Δf of the lasers is 2 kHz. The frequency difference of two lasers is about 30 MHz, which is much larger than the linewidth. The system frequency is 625 MHz.

In the experiment, Alice and Bob both send laser pulses of intensity μ to a measurement site in the middle. Depending on the transmission distance, they will choose a proper μ such that they will get a suitable number of detection results. Besides, they do not perform any phase modulation on these pulses for simplicity. The coherent state prepared by Alice and Bob can be denoted as $|\sqrt{\mu}e^{i\phi^a(t)}\rangle$ and $|\sqrt{\mu}e^{i\phi^b(t)}\rangle$, respectively. The two pulses are interfered at the measurement site and then are detected by single-photon detectors. The detection result is "L" if one of the detectors clicks, and "R" if the other detector clicks.

Now, they do not apply any phase-locking techniques in the demonstration, it is very challenging to track the global phases and predict the measurement result in single round. However, in mode-pairing scheme, we only care about whether the measurement results of the two paired locations are the same or not. Which is determined by the phase difference betweem two paired locations i and j,

$$\theta^\delta = (\phi_j^b - \phi_i^b) - (\phi_j^a - \phi_i^a) = \int_{t_i}^{t_j}\omega_\delta(t)dt, \quad (113)$$

where $\omega_\delta(t) := \omega_b(t)-\omega_a(t)$ is the frequency difference between Alice's and Bob's laser pulses. For a short period of time, we can assume that $\omega_\delta(t)$ drifts with a linear model,

$$\omega_\delta(t) = kt + \omega_\delta(0). \quad (114)$$

After accumulating enough detection results, Alice and Bob can use them to estimate the frequency difference of their lasers during data post-processing. First, they pair the locations where the successful detection occurs. For a pair, there are four possible detection results, (L,R), (R,L), (L,L) and (R,R). One click on location i and the other on j. With the paired locations and corresponding detection results, Alice and Bob can estimate $\omega_\delta(t)$ using a probabilistic model corresponding to this optical setting. Then they can calculate the phase difference θ^δ using Eq. (113).

To test the accuracy of the estimation of $\omega_\delta(t)$ in Eq. (114), we further use the estimated θ^δ to predict the detection results. When the estimated value of θ^δ of a pair is in the region $[-\Delta/2, +\Delta/2)$ or $[\pi-\Delta/2, \pi+\Delta/2)$, this data pair can be further used for testing. Here, $\Delta = 2\pi/32$ is the width of the phase slice. In the former case, Alice and Bob assign the detection (L,R) or (R,L) to be error and (L,L) or (R,R) to be correct; while in the latter case, they assign the detection (L,L) or (R,R) to be error and (L,R) or (R,L) to be correct.

Below we draw the error rate of the paired strong pulses with respect to different pairing length $l = j - i$. We group the paired locations by different pairing length, as shown in Supplementary Figure 22. This error rate can reflect the X-basis error rate in mode-pairing QKD. Similar to the phase-encoding MDI-QKD, the X-basis error has a 25% intrinsic error rate caused by the multi-photon components in the coherent states. From the demonstration we can see that, apart from the intrinsic error, our method only introduces a reasonable additional error rate. One can further reduce the error rate by post-selecting the good signals in post-processing of reference pulses. If we use the decoy method to estimate the phase error in a single photon pair, the error rate will be a reasonable value that we can obtain a positive final key rate. Therefore, the mode-pairing scheme is feasible without global phase locking.

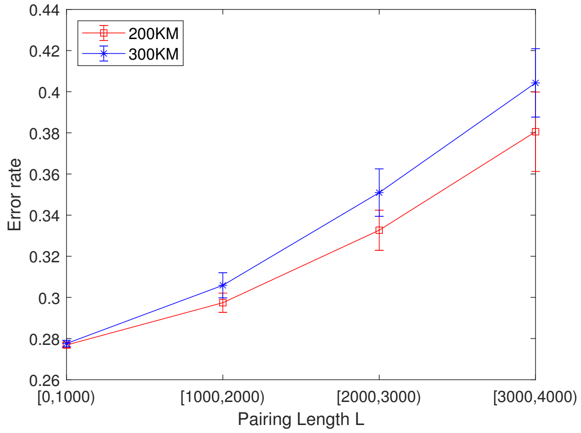

Supplementary Figure 22. Error rates of the paired laser pulses under different pairing intervals and different transmission distances. The intensities of laser pulses are chosen such that the arrived signals on the measurement site are strong enough to provide enough clicks. Given a pairing length interval, say $l \in [1000, 2000)$, we pair all detection clicks such that $1000 \leq j - i < 2000$.

From Supplementary Figure 22, we can see that the error rate is stable when the pairing length is up to $3000 \sim 4000$. If we further enhance the system frequency to 4 GHz [22], we are able to achieve a maximal pairing length of 20000. In this way, the performance of the mode-pairing scheme can surpass the repeaterless key-rate bound. We leave a full demonstration of the mode-pairing scheme for future works.

[1] Z. Cao, Z. Zhang, H.-K. Lo, and X. Ma, Discrete-phase-randomized coherent state source and its application in quantum key distribution, New J. Phys. **17**, 053014 (2015).

[2] P. Zeng, W. Wu, and X. Ma, Symmetry-protected privacy: Beating the rate-distance linear bound over a noisy channel, Phys. Rev. Applied **13**, 064013 (2020).

[3] H. K. Lo and H. F. Chau, Unconditional security of quantum key distribution over arbitrarily long distances, Science **283**, 2050 (1999).

[4] P. W. Shor and J. Preskill, Simple proof of security of the bb84 quantum key distribution protocol, Phys. Rev. Lett. **85**, 441 (2000).

[5] H.-K. Lo, M. Curty, and B. Qi, Measurement-device-independent quantum key distribution, Phys. Rev. Lett. **108**, 130503 (2012).

[6] X. Ma and M. Razavi, Alternative schemes for measurement-device-independent quantum key distribution, Phys. Rev. A **86**, 062319 (2012).

[7] C. H. Bennett, G. Brassard, and N. D. Mermin, Quantum cryptography without bell's theorem, Phys. Rev. Lett. **68**, 557 (1992).

[8] M. Koashi, Simple security proof of quantum key distribution based on complementarity, New Journal of Physics **11**, 045018 (2009).

[9] D. Gottesman, H.-K. Lo, N. Lütkenhaus, and J. Preskill, Security of quantum key distribution with imperfect devices, Quantum Info. Comput. **4**, 325 (2004).

[10] H.-K. Lo, X. Ma, and K. Chen, Decoy state quantum key distribution, Phys. Rev. Lett. **94**, 230504 (2005).

[11] Z. Zhang, Q. Zhao, M. Razavi, and X. Ma, Improved key-rate bounds for practical decoy-state quantum-key-distribution systems, Phys. Rev. A **95**, 012333 (2017).

[12] C.-H. F. Fung, X. Ma, and H. F. Chau, Practical issues in quantum-key-distribution postprocessing, Phys. Rev. A **81**, 012318 (2010).

[13] X. Ma, C.-H. F. Fung, and M. Razavi, Statistical fluctuation analysis for measurement-device-independent quantum key distribution, Phys. Rev. A **86**, 052305 (2012).
[14] M. Curty, F. Xu, W. Cui, C. C. W. Lim, K. Tamaki, and H.-K. Lo, Finite-key analysis for measurement-device-independent quantum key distribution, Nature communications **5**, 3732 (2014).
[15] X. Ma, *Quantum cryptography: from theory to practice*, Ph.D. thesis, University of Toronto (2008), also available in arXiv:0808.1385.
[16] X. Ma, P. Zeng, and H. Zhou, Phase-matching quantum key distribution, Phys. Rev. X **8**, 031043 (2018).
[17] X.-B. Wang, Z.-W. Yu, and X.-L. Hu, Twin-field quantum key distribution with large misalignment error, Phys. Rev. A **98**, 062323 (2018).
[18] C. Jiang, Z.-W. Yu, X.-L. Hu, and X.-B. Wang, Unconditional security of sending or not sending twin-field quantum key distribution with finite pulses, Phys. Rev. Applied **12**, 024061 (2019).
[19] S. Pirandola, R. Laurenza, C. Ottaviani, and L. Banchi, Fundamental limits of repeaterless quantum communications, Nat. Commun. **8**, 15043 (2017).
[20] X. Ma, B. Qi, Y. Zhao, and H.-K. Lo, Practical decoy state for quantum key distribution, Phys. Rev. A **72**, 012326 (2005).
[21] X.-T. Fang, P. Zeng, H. Liu, M. Zou, W. Wu, Y.-L. Tang, Y.-J. Sheng, Y. Xiang, W. Zhang, H. Li, *et al.*, Implementation of quantum key distribution surpassing the linear rate-transmittance bound, Nature Photonics , 1 (2020).
[22] S. Wang, Z.-Q. Yin, D.-Y. He, W. Chen, R.-Q. Wang, P. Ye, Y. Zhou, G.-J. Fan-Yuan, F.-X. Wang, Y.-G. Zhu, P. V. Morozov, A. V. Divochiy, Z. Zhou, G.-C. Guo, and Z.-F. Han, Twin-field quantum key distribution over 830-km fibre, Nature Photonics 10.1038/s41566-021-00928-2 (2022).
